# Distinct patterns of complex rearrangements and a mutational signature of microhomeology are frequently observed in *PLP1* copy number gain structural variants

**DOI:** 10.1186/s13073-019-0676-0

**Published:** 2019-12-09

**Authors:** Vahid Bahrambeigi, Xiaofei Song, Karen Sperle, Christine R. Beck, Hadia Hijazi, Christopher M. Grochowski, Shen Gu, Pavel Seeman, Karen J. Woodward, Claudia M. B. Carvalho, Grace M. Hobson, James R. Lupski

**Affiliations:** 1 Graduate Program in Diagnostic Genetics, School of Health Professions, The University of Texas MD Anderson Cancer Center, Houston, TX USA; 2 Present address: Graduate School of Biomedical Sciences, The University of Texas MD Anderson Cancer Center UTHealth, Houston, TX USA; 3 Department of Molecular and Human Genetics, Baylor College of Medicine, One Baylor Plaza, Room 604B, Houston, TX USA; 4 Nemours Biomedical Research, Nemours/Alfred I. DuPont Hospital for Children, 1600 Rockland Road, RC1, Wilmington, DE USA; 5 Present address: Department of Genetics and Genome Sciences, University of Connecticut Health Center and the Jackson Laboratory for Genomic Medicine, Farmington, CT USA; 6 School of Biomedical Sciences, Faculty of Medicine, The Chinese University of Hong Kong, Shatin, NT Hong Kong SAR; 7 DNA Laboratory, Department of Pediatric Neurology, 2nd Faculty of Medicine, Charles University in Prague and University Hospital Motol, 150 06 Prague, Czech Republic; 8 Clinical and Molecular Genetics Unit, Institute of Child Health, London, UK; 9 Present address: Diagnostic Genomics, PathWest Laboratory Medicine, Perth, WA Australia; 10 School of Biomedical Sciences, University of Western Australia, Perth, WA Australia; 11 Jefferson Medical College, Thomas Jefferson University, Philadelphia, PA USA; 12 Department of Biological Sciences, University of Delaware, Newark, DE USA; 13 Department of Pediatrics, Baylor College of Medicine, Houston, TX USA; 14 Human Genome Sequencing Center, Baylor College of Medicine, Houston, TX USA; 15 Texas Children’s Hospital, Houston, TX USA

**Keywords:** PMD, Genomic rearrangements, Genome instability, Duplication, LCR, RBM, HR, BIR, MMBIR, Microhomeology

## Abstract

**Background:**

We investigated the features of the genomic rearrangements in a cohort of 50 male individuals with *proteolipid protein 1* (*PLP1*) copy number gain events who were ascertained with Pelizaeus-Merzbacher disease (PMD; MIM: 312080). We then compared our new data to previous structural variant mutagenesis studies involving the Xq22 region of the human genome. The aggregate data from 159 sequenced join-points (discontinuous sequences in the reference genome that are joined during the rearrangement process) were studied. Analysis of these data from 150 individuals enabled the spectrum and relative distribution of the underlying genomic mutational signatures to be delineated.

**Methods:**

Genomic rearrangements in PMD individuals with *PLP1* copy number gain events were investigated by high-density customized array or clinical chromosomal microarray analysis and breakpoint junction sequence analysis.

**Results:**

High-density customized array showed that the majority of cases (33/50; ~ 66%) present with single duplications, although complex genomic rearrangements (CGRs) are also frequent (17/50; ~ 34%). Breakpoint mapping to nucleotide resolution revealed further previously unknown structural and sequence complexities, even in single duplications. Meta-analysis of all studied rearrangements that occur at the *PLP1* locus showed that single duplications were found in ~ 54% of individuals and that, among all CGR cases, triplication flanked by duplications is the most frequent CGR array CGH pattern observed. Importantly, in ~ 32% of join-points, there is evidence for a mutational signature of microhomeology (highly similar yet imperfect sequence matches).

**Conclusions:**

These data reveal a high frequency of CGRs at the *PLP1* locus and support the assertion that replication-based mechanisms are prominent contributors to the formation of CGRs at Xq22. We propose that microhomeology can facilitate template switching, by stabilizing strand annealing of the primer using W-C base complementarity, and is a mutational signature for replicative repair.

## Background

Architectural features of the human genome, such as low copy repeats (LCRs) or segmental duplications (SegDup), are associated with genome instability and large-scale genomic changes [1–3]. Copy number gain events associated with LCRs at chromosome X, region Xq22.2, are the most frequent cause of neurological genomic disorders including Pelizaeus-Merzbacher disease (PMD; MIM: 312080) [3]. PMD is a rare hypomyelinating leukodystrophy, predominantly arising from mutations involving the dosage-sensitive proteolipid protein 1 gene (*PLP1*, MIM 300401) [4, 5].

At the *PLP1* locus, nucleotide substitutions and copy number gain events are associated with PMD [6–11] with *PLP1* duplications accounting for ~ 60–70% of PMD cases [12, 13]. Genomic rearrangements in the *PLP1* locus are nonrecurrent, i.e., unrelated individuals carry CNVs with breakpoint junctions and genomic content that vary while sharing a region of overlap including the dosage-sensitive gene [14, 15]. In contrast, rearrangements in the majority of well-defined genomic disorders such as Charcot-Marie-Tooth disease type 1A (CMT1A; MIM: 118220) are recurrent [16], arising from non-allelic homologous recombination (NAHR) between the paralogous genomic segments of the LCR [2, 16–18]. The role of repetitive features, such as LCRs, short interspersed nuclear elements (SINEs; particularly *Alu* elements), and long interspersed nuclear elements (LINEs), in nonrecurrent rearrangements is less well-defined.

Mutagenesis mechanisms that underlie structural variation in nonrecurrent rearrangements include non-homologous end joining (NHEJ), microhomology-mediated end joining (MMEJ), break-induced replication (BIR), and Fork Stalling and Template Switching (FoSTeS)/microhomology-mediated break-induced replication (MMBIR) [19]. Repetitive sequences have been proposed to facilitate the formation of nonrecurrent genomic rearrangements in PMD [14, 15, 20]. In addition, complex genomic rearrangements (CGR), i.e., rearrangements consisting of more than one breakpoint junction and often more than one genomic interval of copy number change, can be observed at loci with susceptibility to nonrecurrent rearrangements [21]. Replication-based mechanisms such as FoSTeS/MMBIR have been proposed to underlie the formation of CGR as a result of iterative template switches (TSs) during replicative repair of a single-ended, double-stranded DNA break (seDSB) [22]. The *PLP1* locus has been reported to have an excess of CGR in association with PMD; some CGR such as complex duplication-triplication-duplication (DUP-TRP-DUP) can cause a more severe PMD phenotype when *PLP1* maps to the triplicated interval [23–26].

**Fig. 1:**
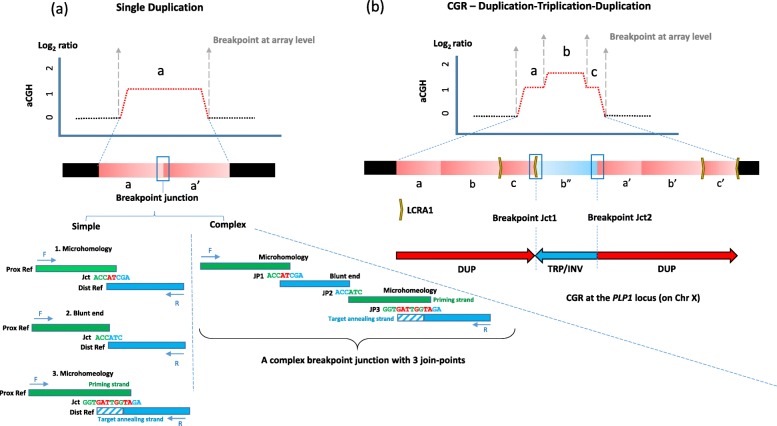
Genomic rearrangements with different levels of complexity. At the array-resolution level, genomic rearrangements with the *PLP1* gain can be apparently simple as **a** a single duplication or **b** a CGR. In aCGH figures, transitions of copy number alterations from copy neutral regions (black dots) to copy number gains (red dots) are demonstrated by gray vertical dashed arrows (breakpoints). At the nucleotide sequence level as shown in **a**, in the simplest case scenario, a single duplication has a breakpoint junction with only one join-point (**a**—left), a product of one TS by NHEJ (for blunt end), or microhomology and/or microhomeology-mediated rearrangement. Or, a breakpoint junction can contain several join-points (**a**—right). Such breakpoint junctions are products of iterative TS by different rearrangement mechanisms such as NHEJ or MMBIR. Bases indicated in red are in both the proximal and distal reference sequences. Rectangle with diagonal lines indicates a region of imperfect match between proximal and distal reference sequences. In addition to the iterative TS that lead to the appearance of complex breakpoints, iterative TS can result in copy number transitions of large genomic segments and formation of more complex genomic structures. **b** As a representative of such complex genomic structures, a schematic figure of a CGR with DUP-TRP/INV-DUP pattern resulted from two TSs creating breakpoint junctions Jct1 and Jct2, as shown. The horizontal bar below the aCGH depicts the rearrangement product. Duplications are represented in red and triplication in blue; yellow arrows represent inverted low copy repeats that mediate the TS in Jct1. Positions of the genomic segments are denoted as a, b, and c, duplicated segments as a′, b′, and c′, and the triplicated segment as b″. The TS between low copy repeats forming Jct1 switched the direction of replication resulting in an inversion of the TRP segment, and the second TS forming Jct2 switched the direction of the replication again resulting in directly oriented DUP segments. The *Y*-axis on the aCGH plots represents expected log_2_ ratios in male using a gender-matched control and that *PLP1* maps to chromosome X. Jct: junction; JP: join-point

Key to the delineation of structural variant mutagenesis mechanisms has been the determination of copy number states at a given locus that deviate from a control diploid genome and the delineation of breakpoint junctions. Breakpoint junctions are the end-products of recombination between substrate pairs in which the individual substrate sequences map to two different positions on the haploid reference genome (Fig. 1a). Breakpoint junctions seen on array comparative genomic hybridization (aCGH) are signified by a transition state from normal copy number to gain or loss of genomic segments. At the nucleotide sequence level, the breakpoint junction may reveal specific “signature sequences” that can include microhomology, blunt-end fusion of DNA substrate sequences, or the relatively newly recognized microhomeology (Fig. 1a). Microhomology refers to sequence identity (usually 2–9 bp) found at the recombinant junction and represented in both sequences of the substrate pair, but reduced from 2 to 1 copy at the junction. It has been proposed that microhomology facilitates TS and is consistent with non-homologous recombination because the extent of homology is far below the minimal efficient processing segment for homologous recombination (HR) [22, 23, 27, 28]. By comparison, microhomeology refers to highly similar (cutoff at 70% homology) yet imperfect sequence matches or alignments of 5 bp or more, a signature that was recently observed in individuals carrying multiple de novo CNVs on multiple autosomes and genomic-disorder-associated rearrangements at 17p11.2 [29, 30].

Iterative TS can result in complexities at breakpoint junctions with multiple join-points (Fig. 1a) wherein discontinuous sequences in the haploid reference are apparently “stitched” together in a template-driven directional way (i.e., priming strand versus target annealing strand) [29]. Single duplications show one prominent copy number gain by aCGH (Fig. 1a) and most are tandem. CGRs can result from TS separated by large DNA distances, kilobase or even megabases (e.g., a DUP-TRP-DUP pattern, Fig. 1b) [26, 31].

Due to the relative rarity of PMD and the limited genomic resolution of clinical testing, the frequency of each particular type of CGR and the mutational signature(s) accompanying mutagenesis remain elusive. Investigating the complexities of genomic architecture and rearrangements at the *PLP1* locus provides insights into the underlying mechanisms of genomic rearrangements in PMD. In addition, understanding architectural features of the genome potentially rendering susceptibility to genomic instability may help to predict loci with inherent genome instability [32, 33]. To further investigate mutational mechanisms involved in genomic rearrangements associated with PMD, we studied a cohort of 50 unrelated individuals who were previously identified with increased *PLP1* copy number by clinical testing. We subsequently performed breakpoint junction mapping to uncover potential genomic complexities and to further delineate potential mutational signatures of genomic disorders. Here, we describe the distribution of different types of rearrangements, and for the first time, we provide robust experimental evidence for microhomeology as a mutational signature at breakpoint junctions at the *PLP1* locus, and discuss potential mechanisms for strand invasion and primer annealing facilitating TS. Finally, we perform a meta-analysis of genomic rearrangements at the *PLP1* locus and summarize findings from a combined data set of 150 individuals harboring *PLP1* copy number gains, including our current study and 6 previous investigations [14, 20, 23, 31, 34, 35]. This larger perspective allows us to derive insight into mutational signatures accompanying genomic rearrangements at the *PLP1* locus.

## Methods

### Human subjects

A total number of 50 male individuals with PMD were identified with an increased *PLP1* gene copy number. Before performing customized high-resolution aCGH, most cases had been tested by either Affymetrix whole-genome microarray or NimbleGen X chromosome array and all cases had been tested by multiplex quantitative PCR throughout duplicated regions as described [14]. Extent of duplicated region but not breakpoint junctions were reported previously for BAB8920 through BAB8933, and BAB3259 as P130, P149, P215, P227, P234, P288, P290, P307, P356, P379, P389, P447, P513, P541, P561, and P119, respectively [14]. Extent of the two duplicated regions and the junction of the distal duplication were reported previously for BAB8962 as P015 [14].

### Targeted array CGH analyses

To fine map the genomic rearrangements to genome-level resolution, we used a custom-designed, high-density oligonucleotide array from Agilent. The array comprises approximately 44,000 interrogating oligonucleotides spanning chrX: 98,028,855-113,513,744 (NCBI build 37/hg19) with an average genome resolution of 386 bp between probes (chrX: 97,915,511-113,400,000 in NCBI build 36/hg18 was converted to GRCh37/hg19 using UCSC Genome Browser; https://genome.ucsc.edu/cgi-bin/hgLiftOver). The experimental procedures were performed according to the manufacturer’s protocol (Agilent Oligonucleotide Array-Based CGH for Genomic DNA Analysis, Version 7.2, Agilent Technologies) with some modifications as described [26, 36]. Gender-matched control DNA from Coriell repository (male individual NA10851) was used for hybridization. Agilent Feature Extraction software and Agilent Genomic Workbench (version 7.0.4.0) were used to process scanned array images (version10) and analyze extracted files, respectively.

### Whole-genome aCGH analysis

A whole-genome Cytogenetics 2.7M array (Affymetrix) was performed at the Coriell Institute Sequencing and Microarray Center to determine copy number changes on chromosome Yq of individual BAB8921. The array had an average marker spacing of 1086 bases between probes. The NCBI build 36/hg18 coordinates were converted to GRCh37/hg19 by using the Lift Genome Annotations tool at https://genome.ucsc.edu/cgi-bin/hgLiftOver.

### Chromosomal microarray analysis

Rearrangements in individual BAB8934 exceeded the coverage of our custom-designed high-density aCGH. A custom-designed oligoarray, BCM V11.2, was performed for this individual as described [37]. The chromosomal microarray analysis (CMA) array was designed using the Agilent Technologies platform to detect copy number changes in clinically significant regions of the entire genome. It comprises approximately 400,000 oligonucleotides and targets over 4200 genes at the exon level (based on GRCh37/hg19 assembly). Gender-matched controls were used for hybridization. The experimental procedures and data analysis were performed as described for targeted aCGH analysis.

### Single nucleotide polymorphism genotyping

Sample BAB8959 was genotyped using an Agilent Infinium CoreExome-24 version 1.3 genome-wide single nucleotide polymorphism (SNP) array at the human genome sequencing center (HGSC) at Baylor College of Medicine in Houston, TX. Of the 240,000 SNPs present on the array, 60 were located within the duplication of this sample for which the genotype was individually assessed.

### FISH analysis

A lymphoblastoid cell line was cultured from patient BAB8921 according to standard protocols. Metaphase chromosomes and interphase nuclei were prepared from the cell line and FISH was performed as described using a cosmid DNA probe containing the *PLP1* gene (cU125A1) and an X-centromeric probe [38].

### Breakpoint junction sequencing

Genomic positions of putative breakpoint junctions for CNVs were identified using the coordinates of interrogating oligonucleotides mapped to the upstream and downstream ends of each CNV. For both array-based single duplications as well as CGRs, outward primers were designed inside the duplication and close to predicted breakpoints. PCR was performed assuming the duplicated sequences are in a tandem orientation for single duplications or using a combination of outward primers (designed inside duplications) for CGRs. For deletions, inward primers were designed outside of the deleted regions. Breakpoint junctions were obtained by long-range PCR using TaKaRa LA Taq according to the manufacturer’s protocol (TaKaRa Bio Company, Cat.No.RR002). The experimental procedures were performed as described [31]. Patient-specific PCR products were purified with Zymoclean Gel DNA Recovery Kit (Zymo Research, Cat. No. D4001). Purified PCR products were then sequenced by Sanger dideoxy sequencing (BCM Sequencing Core, Houston, TX, USA). If necessary, internal primers were designed to “genomically walk” through the product and delineate the junction point. Sequence analysis was conducted using the Lasergene9 DNA analysis software suite. To map breakpoint junctions at the nucleotide level, DNA sequences resulting from Sanger sequencing of breakpoint spanning amplification products were aligned to the reference genome sequence (UCSC genome browser, GRCh37/hg19).

### Characterization of microhomology and microhomeology

We aligned the breakpoint junction sequence with the proximal and distal ends of each breakpoint using the reference genome. Shared 100% nucleotide identity between the 5′ and 3′ reference strands at the join-point was considered microhomology [3]. Imperfect matches at the join-points (cutoff of 70% identity for a stringent threshold with a maximum 2-nt gap) involving ≥ 5 bp were also determined. In this study, such imperfect matches or microhomeology, varying from 71 to 92% identity at the junctions, were recently reported as a feature associated with individuals carrying multiple de novo CNVs that originated from a replication-based mechanism [29]. We further required ≥ 2-bp matched sequences following a two-nucleotide gap to lower the impact of spurious match and apparent microhomeology due to random events. Repetitive sequence-mediated rearrangement events that resulted from *Alu-Alu* or LINE-LINE recombination (chimeric *Alu* or LINE elements) or homologous recombination between two highly similar non-allelic DNA sequences (NAHR) were not included in the meta-analysis when calculating microhomology or microhomeology at breakpoint junctions.

### Breakpoint junction sequence similarity analysis

We analyzed the similarity of DNA sequences that are surrounding breakpoints using the R programming language [39]. We first obtained the 300-bp reference sequences at the breakpoints. We then manually aligned the junctions to reach 100% shared identity (microhomology) or imperfect identity (microhomeology). The sequences flanking each breakpoint junction were then aligned with each microhomology/microhomeology in the center using the Needleman-Wunsch algorithm, Biostrings package (http://bioconductor.org/packages/Biostrings). We then calculated the sequence similarity within a 20-bp moving window as the percentage of aligned bases over the total count of non-gap sequences, for which orientation relies on the alignment with DNA sequence across the breakpoint junctions. We further show this similarity pattern by plotting a heat map for each event. In addition, we compared the similarity patterns among four groups of reference sequence alignments: both sides of blunt junctions, both sides of junctions with a microhomology only, the priming sides or the target annealing sides of junctions having a microhomeology, which could contain a microhomeology only or include both a microhomology and a microhomeology. For each group and every base pair within 150 bp from the breakpoint junctions (edges of a microhomology or microhomeology), we summarized the similarity levels by calculating mean values. We presented the change of the averaged similarity level along an increase in the distance to the break junctions by plotting a dot plot with a smooth regression line.

## Results

### Single genomic duplications and CGRs were detected by aCGH at the *PLP1* locus

**Fig. 2:**
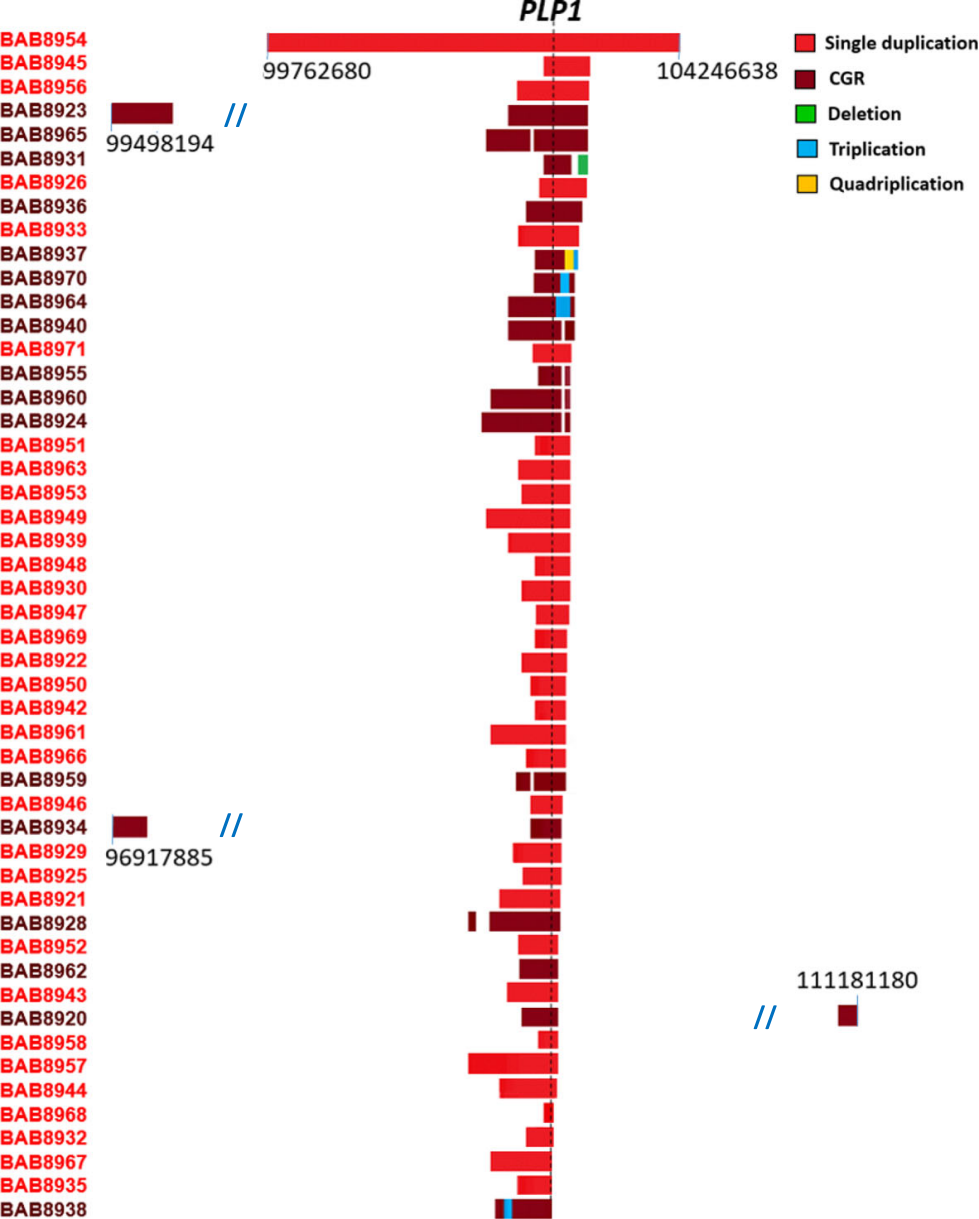
An overview of genomic rearrangements as seen on aCGH in 50 individuals with PMD. Genomic rearrangements at Xq22 vary in size and genomic positions. The largest duplication (~ 4.5 Mb) is found in individual BAB8954. Three individuals show additional duplications distant from the duplicated *PLP1* locus (individuals BAB8920, BAB8923, and BAB8934). The black numbers refer to genomic coordinates on chromosome X. The left column lists the 50 subjects studied. Slash lines indicate a break in numbering for genomic coordinates. The location of *PLP1* is indicated by a black vertical broken line

We performed custom-designed aCGH to better understand the full spectrum of copy number alterations at the *PLP1* locus. Results showed that rearrangement products were nonrecurrent (Fig. 2). Single duplications varying from ~ 122 kb to ~ 4.5 Mb were seen in 66% of cases (33/50) (Additional file 1: Figures S1-S4 and Table 1, and Additional file 2: Table S1). The smallest region of overlap (122 kb), which included genes *GLRA4*, *TMEM31* (embedded within *GLRA4*), and *PLP1*, is represented by the duplication in individual BAB8968 (Additional file 1: Figure S1–6). The largest duplication was found in individual BAB8954 and spanned ~ 4.5 Mb including 62 genes (ChrX: 99,762,680-104,246,638, GRCh37/hg19) (Additional file 1: Figure S1–4).

**Table 1:** Genomic rearrangement pattern at the *PLP1* locus in this study

Rearrangement product pattern	Frequency (*N* = 50)
Single duplication	66% (33/50)
DUP-NML-DUP	18% (9/50)
DUP-TRP-DUP	6% (3/50)
Other CGR	10% (5/50)

**Fig. 3:**
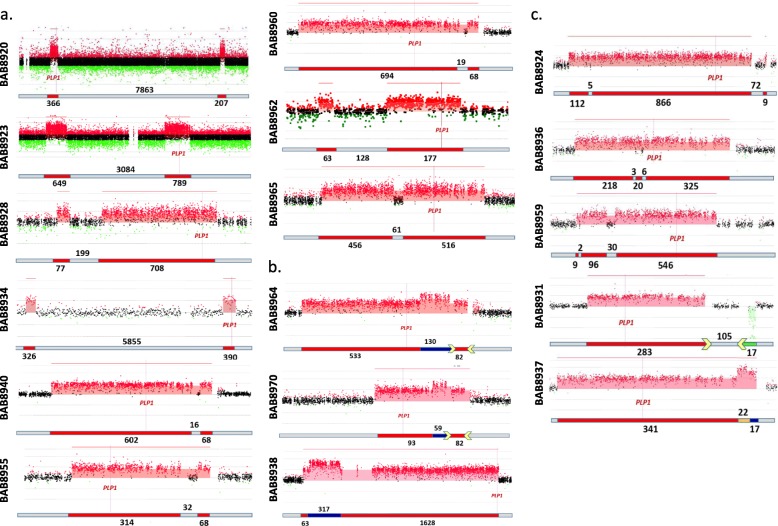
CGRs detected by aCGH at the *PLP1* locus. **a** Two duplications separated by CNRs were detected on aCGH in 9 individuals with PMD. The distance between the two duplications differs among these individuals, ranging from 16 to 7863 kb. In the schematic figure below each array, duplications are depicted in red and CNRs in gray. Three cases (BAB8940, BAB8955, and BAB8960) could be single duplications on the H2 inversion haplotype or could be two duplications with one TS involving reversal of the direction of replication between IRs LCRA1a and LCRA1b (Additional file 1: Figure S9); three (BAB8923, BAB8928, and BAB8965) have directly oriented DUP-NML-DUP structures (Additional file 1: Figures S6–1, S6–2 and S6–3); one has two tandem head to tail duplications (BAB8962; Additional file 1: Figure S6–4); and two (BAB8920, BAB8934) have DUP-NML-INV/DUP structures (Additional file 1: Figure S7). **b** A DUP-TRP-DUP pattern of rearrangement was detected on aCGH in three individuals with PMD (Additional file 1: Figure S10). Breakpoint junction analyses indicated that one of these individuals (BAB8964) probably has the previously reported DUP-TRP/INV-DUP pattern of rearrangement with inversion mediated by a TS between inverted repeats LCRA1a and LCRA1b. Based on aCGH data, BAB8970 probably has the same structure, although breakpoint junctions were not resolved (Additional file 1: Figures S10–1 and S10–2). Breakpoint junction analysis indicates that BAB8939 also carries a DUP-TRP/INV-DUP, but the inversion was not mediated by LCRA1a and LCRA1b (Additional file 1: Figure S10–3). Duplications are indicated in red, triplications in blue, and LCR blocks (LCRA1a and LCRA1b) in yellow. **c** Additional CGR patterns at the *PLP1* locus were identified on aCGH. DUP-NML-DUP-NML-DUP rearrangement pattern in which duplications are separated by short CNRs (BAB8924, BAB8936, and BAB8959). In BAB8924, based on the sequenced breakpoint junction, this case may have two tandem head to tail duplications on the H2 haplotype that has an inversion within LCRA1a and LCRA1b (Additional file 1: Figure S12–1a) or may have three duplications with one TS between LCRA1a and LCRA1b resulting in an inversion (not shown)**.** We were not able to resolve any breakpoint junctions in BAB8936 (Additional file 1: Figure S12–1b). Breakpoint junction sequencing in BAB8959 showed that the CGR based on aCGH may not have occurred during the same cell division (Additional file 1: Figures S12–2). One individual, BAB8931, exhibited DUP-NML-DEL pattern of rearrangement with a ~ 283-kb duplication (breakpoint junction in LCRA1a) followed by ~ 106 kb of CNR and then a ~ 16-kb deletion (breakpoint junction in LCRA1b). The most complex rearrangement in this study was observed in individual BAB8937 with a DUP-QUAD-TRP rearrangement pattern. In this case, duplication is followed by a quadruplication and then a triplication. The possible mechanism for such rearrangements is shown in Additional file 1: Figure S11. Duplications are indicated in red, CNRs in gray, deletion in green, triplication in blue, quadruplication in orange, and LCR blocks in yellow in the horizontal bar under each array

We detected CGRs in 17 individuals (34%) (Table 1 and Additional file 2: Table S2). Nine had an aCGH pattern of interspersed duplications separated by a copy neutral region (CNR), a pattern previously described as DUP-NML-DUP (Fig. 3a) [3, 14, 37]. In addition, we identified triplication flanked by duplications (DUP-TRP-DUP) in three individuals, 6% of this cohort, a pattern reported previously in PMD cohorts (Fig. 3b) [26, 31]. Rearrangements with other complexities were detected in five individuals (Fig. 3c). A DUP-NML-DUP-NML-DUP pattern was seen in three (BAB8924, BAB8936, and BAB8959); a duplication followed by a CNR and then a deletion, DUP-NML-DEL, was seen in another, BAB8931; and a duplication followed by a distal quadruplication and triplication, DUP-QUAD-TRP, was seen in BAB8937 (Fig. 3c). A quadruplication-containing CGR has been described at the *PLP1* locus [31].

In this cohort, 28 samples (56% of all individuals) have breakpoints that map to a 186-kb genomic interval distal to *PLP1* that contains both direct and inverted LCRs (Additional file 1: Figure S5) [14, 15]. This region consists of repeated segments, e.g., LCRC, LCRA1a, LCR2, LCR3, LCRA1b, and LCRD varying in size from 18.5 to 27.3 kb (ChrX: 103,172,645-103,324,337, GRCh37/hg19 assembly) [14, 15]. The inverted repeat (IR) pair, LCRA1a and LCRA1b, ~ 20 kb in size and of 98.9% nucleotide sequence identity, is the major IR involved with the formation of the triplications at the *PLP1* locus [26, 31]. Out of the 28 cases with breakpoints in this distal interval, 14 of them contain at least one of the breakpoints mapping to LCRA1a or LCRA1b (Additional file 1: Figure S5). The implication of this pair of LCRs is more prominent within CGR events (10 out of 17, ~ 59% of CGR) than within single duplication events (4 out of 33, ~ 12%) (Additional file 1: Figure S1).

### Breakpoint junction analysis of the single duplications reveals complexities

We were able to resolve the breakpoint junctions at nucleotide-level resolution in 27 of the 33 individuals with a single duplication based on aCGH (one breakpoint junction per case with one or more join-points). In 26 out of 27, the breakpoint junction indicated that the rearrangement product was in a head-to-tail orientation (Additional file 2: Table S2, Additional file 1: Figures S1-S3). Most were single join-points with microhomology or microhomeology, and a few had insertion of one or more bases. The breakpoint junction in BAB8949 was an 861-bp insertion that originated from two flanking regions of the proximal (centromeric) end of the duplication, likely resulted from three TS, i.e., FoSTeS X3, one of which was *Alu*Y*/Alu*Y-mediated (Additional file 1: Figure S2) [23]. Because of iterative TSs in this case, the breakpoint junction can be further resolved into three join-points. One other individual, BAB8950, had a templated insertion of 11 bp resulting from two TS (Additional file 1: Figure S1–4). Further, a 7-bp insertion at the breakpoint junction and three small flanking deletions that were absent from the dbSNP database (build 151) were observed in sample BAB8929 (Additional file 1: Figure S3). Replication errors at breakpoint junctions and/or flanking regions, including small deletions, insertions, and single nucleotide variants (SNVs), were also noted in an additional 10 individuals with single duplication (BAB8933, BAB8935, BAB8942, BAB8946, BAB8949, BAB8951, BAB8952, BAB8963, BAB8966, and BAB8969; Additional file 1: Figures S1-S3). Furthermore, in individual BAB8921 with a single duplication, fluorescent in situ hybridization (FISH) indicated that there was an insertional translocation of the *PLP1* locus into a position on chromosome Yq (Additional file 1: Figure S4) [40]. This individual also had two duplicated regions at Yq on whole-genome aCGH in addition to the duplication at the *PLP1* locus. Using the hypothesis that the duplicated *PLP1* locus was inserted between the two copies of a duplication found on chromosome Y, we were able to resolve one of the two breakpoint junctions (Additional file 1: Figure S4) [40]. The other breakpoint junction was not resolved, perhaps due to the highly repetitive sequence at the duplicated region on the Y chromosome.

### Breakpoint junction analysis in individuals with the most common CGR aCGH pattern, DUP-NML-DUP

Breakpoint junction analysis of four of the nine individuals with a DUP-NML-DUP pattern on aCGH (Fig. 3a) revealed that they had two directly oriented duplications with a CNR, i.e., a genomic interval with normal copy located between the duplicated segments (Additional file 1: Figure S6). BAB8923, BAB8928, and BAB8965 each had one breakpoint junction formed by a TS between the distal end of one duplicated segment and the proximal end of another, resulting in the CNR between the two duplications (Additional file 1: Figures S6–1, S6–2, and S6–3, respectively). The second TS was between the distal end of the distal duplication and the proximal end of the proximal duplication, resulting in the duplication of both segments in direct orientation. In the fourth individual with a DUP-NML-DUP pattern, BAB8962, TSs between the proximal and distal ends of each duplication created two separate duplications (Additional file 1: Figure S6–4). Junction sequencing in individual BAB8923 revealed that the first TS (Jct1) was mediated by directly oriented *Alu*s with 90% identity (Additional file 1: Figure S6–1). In Jct2, we found a 3-bp insertion that could be the result of a replication error. In individual BAB8928, both junctions had microhomologies (Additional file 1: Figure S6–2). Junction sequencing of BAB8965 revealed a 38-bp insertion at Jct1 and a 182-bp insertion at Jct2 templated from four different discontinuous genomic segments resulting from six iterative TS events as evidenced by distinguishable join-points (Additional file 1: Figure S6–3). The breakpoint junction sequencing of BAB8962 revealed an insertion of 170 bp templated from two genomic regions, one of which is located in the region of the second duplication, suggesting the possibility that both duplications may have occurred during the replication event of one cell division (Additional file 1: Figure S6–4).

In the remaining five individuals with DUP-NML-DUP aCGH patterns, breakpoint junction analysis indicated that an inversion had occurred. Individuals BAB8920 (Additional file 1: Figure S7–1) and BAB8934 (Additional file 1: Figure S7–2) had a DUP-NML-INV/DUP structure. The TS at one breakpoint junction occurred between the distal ends of the two duplicated segments and the TS at the other was between the proximal ends, giving rise to an inverted duplicated segment (Additional file 1: Figure S7). There are three potential rearrangement structures that satisfy the two breakpoint junction sequences found in these individuals (Additional file 1: Figure S8). In addition to the rearrangement structure in which a distal duplicated segment was inverted between two directly oriented copies of the proximal duplicated segments (Additional file 1: Figure S8a), the proximal duplicated segment could be inverted between two directly oriented copies of the distal duplicated segments (Additional file 1: Figure S8b), or both proximal and distal duplicated segments and the CNR between them could be inverted (Additional file 1: Figure S8c). Distinguishing among these rearrangement structures for each individual with DUP-NML-INV/DUP would require additional studies [41]. In individual BAB8920, opposite-oriented LINEs, L1PA5 and L1PA3, with 93% identity mediated one TS (Jct 1) and the second TS was microhomology-mediated (Additional file 1: Figure S7–1). In individual BAB8934, two TS were mediated by microhomeology (2 join-points in Jct1) and a third one (Jct2) was mediated by opposite-oriented *Alu-Alu* (both from *AluSX1* family, 89% identity) (Additional file 1: Figure S7–2).

In three of the five individuals whose breakpoint junction indicated inversion, BAB8940, BAB8955, and BAB8960, the distal duplication maps within IRs LCRA1a to LCRA1b (Additional file 1: Figure S9). At least two structural haplotypes at this locus exist in the human population, the H1 allele with ~ 58% frequency and the H2 inverted allele with ~ 42% frequency (resulting from a recombination event between LCRA1a and LCRA1b). If the LCRA1a/LCRA1b region on the arrays of individuals BAB8940, BAB8955, and BAB8960 is inverted to represent the H2 haplotype, the CNVs are seen to be single duplications, so the aCGH pattern of DUP-NML-DUP may be due to displaying the data of an individual with the H2 inversion haplotype on an array designed using the H1 haploid reference genome (Additional file 1: Figure S9) [31]. The sequenced breakpoint junctions in two of these individuals, BAB8940 and BAB8955, and the ~ 42% population frequency of the H2 haplotype support this hypothesis. Another potential explanation for generation of CNVs in these individuals requires a replicative mechanism with two TS, one facilitated by LCRA1a and LCRA1b that results in an inversion [37, 42]. Detection of the H2 allele in such cases by Southern blot hybridization would help to distinguish the mechanism for CGR formation [31]. Breakpoint junction analysis showed that the duplications of BAB8940 and BAB8955 had microhomeology at their sequenced breakpoint junction (Additional file 1: Figure S9) [30].

Interestingly, directly oriented *Alu*s mediated the DUP-NML-DUP pattern of rearrangement (Additional file 1: Figure S6–1), while oppositely oriented LINEs or *Alu*s mediated the DUP-NML-INV/DUP rearrangement pattern (Additional file 1: Figure S7). Further, in individuals BAB8920, BAB8923, and BAB8934 with relatively large CNR ranging from 3084 to 7863 kb between duplications, *Alu-Alu-* or LINE-LINE-mediated rearrangements are involved in facilitating the long-distance TS events, resulting in a chimeric LINE or *Alu* element at one breakpoint junction (Additional file 1: Figures S6–1 and S7) [29, 37, 43–45].

### Triplication and quadruplication copy number gains at Xq22

In this study, we report three individuals with DUP-TRP-DUP on aCGH (Fig. 3b and Additional file 1: Figure S10). We previously reported that individuals with this aCGH pattern at the *MECP2* and *PLP1* loci had an inversion, and we proposed a mechanism of TS between IRs for formation of the DUP-TRP/INV-DUP structure [24, 35]. We also provided evidence that two IRs, LCRA1a and LCRA1b (~ 20 kb each), mediate those events at the *PLP1* locus [20, 31], analogous to rearrangements at the *MECP2* locus [26]. Breakpoint junction analysis in BAB8964 showed that the breakpoint junction is characteristic of this DUP-TRP/INV-DUP pattern, i.e., Jct1 joining the distal end of the distal duplicated region with the distal end of the triplicated region forming a chimeric LCR (LCRA1a/LCRA1b), which is at the same location in each patient, and Jct2 joining the proximal end of the triplicated region with the proximal end of the proximal duplicated region, which varies in location from patient to patient (Additional file 1: Figures S10–1). Analogous to the *Alu*- and LINE-mediated events in DUP-NML-INV/DUP individuals (Additional file 1: Figure S7), the LCR-mediated events in DUP-TRP/INV-DUP individuals result in the formation of an LCRA1a/LCRA1b chimeric element by NAHR along with inversion of the triplicated region, since LCRA1a and LCRA1b are in inverted orientations with respect to each other in the reference genome. We were not able to resolve breakpoint junctions in another individual with a DUP-TRP-DUP pattern on aCGH involving IRs LCRA1a and LCRA1b, BAB8970, but the rearrangement could be DUP-TRP/INV-DUP, as in those previously reported and in BAB8964 in this report (Additional file 1: Figure S10–2).

In the rearrangement of the third individual with a DUP-TRP-DUP structure, BAB8938, the triplication did not border the LCRs and was in a different region from that in the other two patients with the DUP-TRP-DUP structure in this report and in previously published individuals with triplication (Additional file 1: Figure S10–3) [31]. Rather, it was situated 1612 kb proximal to that of *PLP1*. We obtained Jct1 in which it can be surmised that a TS occurred between the distal end of the triplicated region and the distal end of the distal duplicated region in an inverted orientation, i.e., this individual also has a DUP-TRP/INV-DUP structure, but it does not involve LCRA1a and LCR1b as in the previously reported DUP-TRP/INV-DUP individuals and in BAB8964 and BAB8970 (Additional file 1: Figures S10–1 and S10–2) [31]. The sequence across this breakpoint junction has an interesting templated insert structure of three direct repeats (indicated by pink, blue, and yellow curved arrows) and a short IR of 10 bases (indicated by curved green arrow). The IR could be indicative of a TS that inverts the direction of replication at this breakpoint junction. We were not able to resolve a second breakpoint junction for this individual, but the proposed Jct2 is shown (Additional file 1: Figure S10–3).

The most complex rearrangement in this study was observed in individual BAB8937 who carries a duplication followed by a quadruplication and a triplication (Additional file 1: Figure S11). Previously, breakpoint junction analysis in another individual with this pattern of rearrangement revealed three breakpoint junctions of which two (Jct1 and Jct2) were identical and the third was likely due to a TS between the proximal end of the quadruplicated genomic interval and the distal end of duplication [31]. The rearrangement in BAB8937 is potentially characterized by the same pattern but only Jct3 could be sequenced despite our numerous attempts to obtain Jct1 and 2 (Additional file 1: Figure S11). Based on the sequenced junction (Jct3), there is a TS between the distal end of quadruplication and the proximal end of duplication, so the rearrangement observed in this patient is in reverse orientation from the previously reported one [31]. The position of Jct1 and Jct2 at LCR2 and LCRA1b, respectively, and the 88% homology between the two LCRs suggest that multiple TS events between these two repeats could have been involved in the formation of this CGR.

### CGRs in individuals with multiple CNRs or deletion(s)

Our high-resolution aCGH platform could detect altered CNRs as small as 2 kb represented by 9 to 11 interrogating probes, allowing us to detect a complex DUP-NML-DUP-NML-DUP pattern in three individuals, BAB8924, BAB8936, and BAB8959 (Fig. 3c and Additional file 1: Figure S12). In individual BAB8924, a ~ 987-kb duplication, a small CNR of ~ 5 kb, and a larger CNR of ~ 72 kb were observed (Fig. 3c). In individual BAB8936, two small CNRs of ~ 3 kb and ~ 6 kb (Fig. 3c), and for individual BAB8959 a small CNR of ~ 2 kb and a relatively large CNR of ~ 30 kb were detected within CGRs (Fig. 3c).

In individual BAB8924, the 72-kb CNR maps within IRs LCRA1a to LCRA1b (Additional file 1: Figure S12–1a), like CNRs in DUP-NML-DUP individuals BAB8940, BAB8955, and BAB8960 (Additional file 1: Figure S9). As in those individuals, the resolved breakpoint junction indicated inversion, and the rearrangement in BAB8924 may have occurred on the H2 haplotype (Additional file 1: Figure S12–1a) [31]. Thus, although we were not able to resolve a second breakpoint junction, it is possible that BAB8924, like BAB8962 (Additional file 1: Figure S6–4), has two separate tandem head to tail duplications, with a small CNR between them. Alternatively, BAB8924 could have three duplications with one of the junctions involving TS between LCRA1a and LCRA1b resulting in inversion (not shown). At the breakpoint junction of DUP2 in BAB8924, we identified an insertion with two flanking microhomeologies, likely join-points as a product of iterative TS. Therefore, there is a small insertion (27 bp) between first and second copies of the second duplication (Additional file 1: Figure S12–1a). We were not able to amplify breakpoint junctions in BAB8936 (Additional file 1: Figure S12–1b).

Individual BAB8959 had breakpoint junctions for two deletions and a duplication (Additional file 1: Figure S12–2). Jct1, the duplication breakpoint junction, was indicative of a tandem head-to-tail duplication encompassing the duplicated region on aCGH, and the other two, Jct2 and Jct3, were indicative of deletions in one copy of the duplicated region. We checked the database of genomic variants (DGV) to determine whether a CNV polymorphism could explain either of the CNRs. There are three CNVs in the DGV that colocalize with the 30 bp deletion in Jct3 of our patient, one of which, esv2672539, has the same bases deleted as our patient (Additional file 1: Figure S12–2). This deletion was seen in 26 DNAs from 1092 human genomes (population frequency of 2.4%) [46]. The self-chain track in the UCSC Genome Browser revealed the presence of two ~ 700 bp highly identical directly oriented self-chain blocks (90% identity) in the reference genome (chrX + 102,757 K, block 7/22, chrX: 102,778,586–102,779,195 [609 bp] and chrX + 102,757 K, block 7/22, chrX: 102,808,754-102,809,494 [740 bp], GRCh37/hg19) that could have mediated the deletion TS by NAHR (Additional file 1: Figure S12–2). In addition to this deletion, there is a small microhomeology-mediated deletion close to the proximal end of duplication (Jct2). In order to determine whether the duplication in BAB8959 arose at the same time with deletions in an intrachromosomal event or occurred as an ancestral event by an interchromosomal TS between two homologous chromosomes, we used an Illumina Human Core Exome Array to evaluate SNPs within the duplicated region. Of the 60 SNPs within this region, none were dimorphic, providing evidence that deletions and the duplication were likely formed during an intrachromosomal event (Additional file 1: Figure S12–2).

Interestingly, individual BAB8931 exhibited a DUP-NML-DEL pattern of rearrangement on aCGH that consists of an ~ 283-kb duplication with distal breakpoint mapped to the proximal end of LCRA1a, followed by ~ 106 kb of CNR and then an interstitial ~ 16-kb deletion whose proximal breakpoint maps to the distal end of LCRA1b (Additional file 1: Figure S13). The rearrangement could be a result of two independent TSs in which the first TS leading to a gain at the *PLP1* locus is facilitated by NAHR between LCRA1a and LCRA1b that reverses the direction of replication, and the second TS that creates the deletion and resolves the direction of replication (Additional file 1: Figure S13). Alternatively, the presence of such a deletion in the ancestral chromosome that underwent an intrachromosomal duplication event may explain the generation of such apparent copy number complexities (Additional file 1: Figure S13). We were not able to resolve breakpoint junctions in BAB8931, and we were not able to further test the second hypothesis, as neither parental nor grandparental samples were available for molecular studies.

### Microhomeology as a mutational signature of replicative repair

**Fig. 4:**
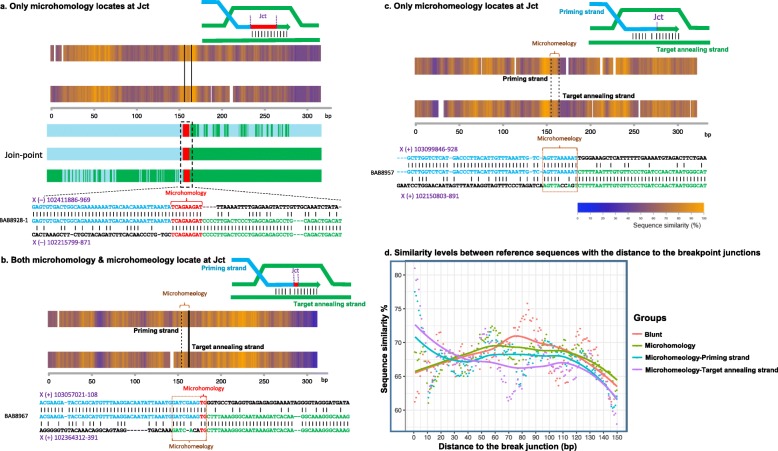
Representative similarity plots (heat maps) between reference sequences surrounding CNV breakpoint junctions containing **a** only microhomology (> 2 bp of nucleotide similarity) flanked by solid vertical lines), **b** both microhomeology and microhomology, and **c** only microhomeology. We present here an example for each type of the observed junctional sequences using heat map (top) and the sequence alignment at a nucleotide level (bottom). Reference sequences were aligned using the Needleman-Wunsch algorithm, as described in the “Methods” section. The 5′ reference sequence is indicated in blue color and 3′ reference sequence is indicated in green. In the upper panel of heat map plot, the 5′ reference sequence was plotted as a rectangle on the top while the 3′ was on the bottom. The heat map shading indicates the sequence similarity level of a 20-bp moving window: orange-high similarity, blue-low similarity, and white-gap. Schematic figures in **b** and **c** indicate microhomeology-mediated priming strand (blue) invasion to the target annealing strand (green). Microhomology is shown in red. **d** An aggregative plot showing the change of similarity levels between reference sequences along an increase in the distance to the breakpoint junctions. We compared such patterns among four junction categories: blunt junctions (red), junctions containing a microhomology only (green), and the priming sides (blue) and target annealing sides (purple) of junctions containing a microhomeology

Microhomology refers to short stretches (2–9 bp) of nucleotide identity between the two substrate reference sequences at breakpoint junctions of genomic rearrangements that facilitate TS and represents one mutational signature of replicative repair including FoSTeS/MMBIR [3, 23] (Fig. 4a). By comparison, when observing base pairs of microhomeology at join-points, these base pairs often show similarity exclusively to one of the two substrate reference sequences; an observation consistent with MMBIR wherein the end of the breakpoint with perfect sequence match to the junction acts as the priming site for TS and the end with imperfect matches serves as the target annealing site of TS invasion (Fig. 4b, c) [29]. In the current cohort (50 cases), 40 samples yielded PCR amplification and sequencing results for at least one breakpoint junction. We found microhomology in 15 out of 57 (~ 26%) sequenced join-points that ranged in size from 2 to 9 bp; evidence for microhomeology was observed in 19 out of 57 join-points (~ 33%); the latter interpreted as reflecting TS facilitated by short segments (≥ 5 bp) with at least 70% identity (Table 2 and Additional file 2: Table S4). The size of the microhomeology ranged from 7 to 14 bp with nucleotide identity ranging from 70 to 90% (Additional file 2: Table S4).

**Table 2:** Sequence characteristics of join-points in the breakpoint junctions from this study and meta-analysis of aggregate data^1^

Product of rearrangement join-point	Frequency (~%, count/sum)	
	This study	Aggregate data^1^
Join-points with 1 bp match	5.3% (3/57)	6.3% (10/159)
Microhomology > 2 bp	26.3% (15/57)	22% (35/159)
Microhomeology^2^	33.3% (19/57)	32.1% (51/159)
*Alu-Alu*	5.3% (3/57)	7.5% (12/159)
LINE-LINE	1.75% (1/57)	1.9% (3/159)
Blunt	3.5% (2/57)	5.7% (9/159)
Insertion^3^	22.8% (13/57)	23.9% (38/159)
Others^4^	1.75% (1/57)	0.6% (1/159)

^1^The aggregate dataset consists of 148 sequenced breakpoint junctions from this study and previously published SV mutagenesis studies involving copy number gains at the Xq22 locus

^2^Re-interpreted data according to new definition of microhomeology. We re-analyzed breakpoint junction data from previous published studies [15, 21, 24, 32, 33]. Although in some cases both a microhomology and a microhomeology occur, microhomologies were not counted when we found microhomeologies at join-points

^3^Small insertion (1 to 20 bp) or larger insertion with unknown origin (> 20 bp)

^4^Rearrangement mediated by two highly identical directly oriented sequences (LCRs or self-chains)

We also found chimeric LINE-LINE or *Alu/Alu* potentially resulted from TS in ~ 7% (4/57) of rearrangements including both single duplications and CGRs (Additional file 2: Table S5). The join-points with small insertions (1–8 bp) contributing to breakpoint junction complexity were observed in 11/57 join-points and large insertions with unknown origin in 2/57 (Additional file 2: Table S5). Join-points with one base pair match or blunt end were less frequently observed (5/57) while one join-point was the result of NAHR mediated by a pair of paralogous repeats identified in the self-chain track (1/57) of the UCSC browser (Additional file 2: Table S5).

We next computationally examined the nucleotide similarity between two substrate reference sequences surrounding each breakpoint junction with microhomology (2 bp or more, 100% match) and/or microhomeology. For this study, we obtained 300 bp of reference sequence with the join-point in the middle for each side of each join-point. Since we noticed that some of the join-points with microhomeology also had microhomology (see “Methods”), the join-points were grouped into three categories: microhomology only, both microhomology and microhomeology, and microhomeology only. One example for each characteristic group is shown in Fig. 2; the computational output for all junctions from this study are summarized in Additional file 1: Figure S14. For each event, 300 bases were examined for sequence similarity between the proximal and distal references such that the reference sequence derived from 150-base extensions of the proximal reference on either side of a join-point was used as the base for alignment on the top plots while that from the distal reference was used as the base for alignment on the bottom plots. The heat map shading indicates the sequence similarity level of a 20-bp moving window, in which orange indicates high similarity, blue indicates low similarity, and white represents gaps in the alignment.

The join-points are mostly in a local region of higher similarity (i.e., more orange) in comparison to its surrounding region (more blue and sometimes containing gaps), indicating that the sequence similarity is not limited to the breakpoint junction and suggesting that TS events might frequently occur in association with such microhomeology blocks in the genome (Additional file 1: Figure S14). We found that in the join-points with both microhomeology and microhomology, in most cases the microhomology locates to one end of the microhomeology or to overlapping microhomologies, one on either end of the microhomeology, supporting the donor-acceptor hypothesis, wherein microhomology facilitates W-C base pair complementarity and strand annealing to prime DNA replication during TSs (e.g., BAB8967 in Fig. 4b, Additional file 1: Figure S14) [29]. However, we also found some cases with microhomology in the middle of microhomeology in which we were unable to define the target annealing and priming strands (e.g., BAB8944 in Additional file 1: Figures S1 and S14). To reveal whether the reference sequences surrounding different categories of junctions would require distinct levels of similarity, we further aggregated the sequence alignments according to the junction category and calculated the averaged similarity level for each base pair that is within 150 bp from the breakpoint. We observed that reference sequences that are at a distance of < 30 bp to a microhomeology could better align with each other than those surrounding a microhomology or a blunt junction, and the target annealing sides overall align better than the priming sides. For reference sequences surrounding a microhomeology, the sequence similarity levels decrease along an increase of the distance to the breakpoint junctions. This could be explained by a better sequence alignment at the priming side that may potentially stabilize the strand annealing of a primer and thus facilitate a template switch (Fig. 4d).

### Meta-analysis of DNA rearrangements and breakpoint junction characteristics at the *PLP1* locus

**Fig. 5:**
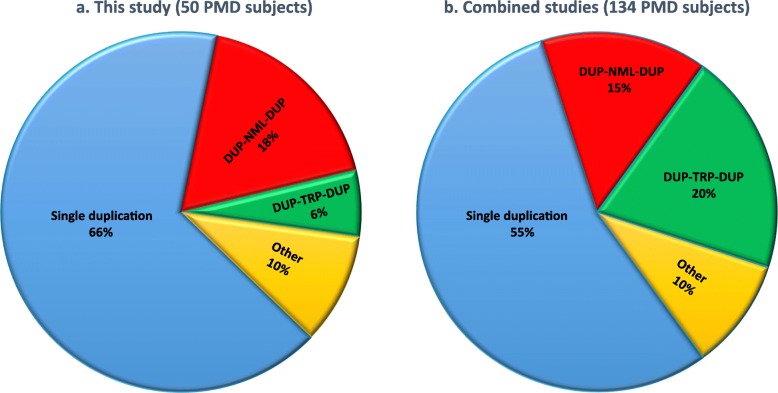
An overview of genomic rearrangements with gain at the *PLP1* locus. **a** Genomic rearrangements in the present cohort with 50 PMD individuals (Table 1). **b** Meta-analysis of combined results from six previously published studies (Additional file 2: Table S3a). Genomic rearrangements involving triplications are the most frequent CGRs at the *PLP1* locus

In aggregate, 159 join-points from 124 unrelated patients with PMD are available for breakpoint junction data meta-analysis at this *PLP1* locus; 61 individuals, i.e., almost half, had a CGR with more than one CNV and showed evidence that multiple copy number variant states were generated in the same structural-variation event, potentially due to iterative TS [14, 20, 23, 31, 34, 35]. The aggregate data were analyzed for general features and characteristics at breakpoint junctions and compared to the human genome reference sequence to identify mutational signatures (Fig. 5 and Table 2).

We re-analyzed breakpoint junction data from previous studies using additional computational analyses described in the “Methods” section; results (including the current cohort) revealed that microhomology is present in ~ 22% (35/159) of join-points, whereas 19/159 (~ 12%) of join-points have ≤ 1 bp match (including join-points with blunt ends) (Table 1). Microhomeology was observed in 51/159 (~ 32%) of reported join-points (Table 1, Additional file 2: Tables S4 and S6). Heat map similarity analyses between the reference sequences surrounding each breakpoint junction with microhomology (2 bp or more, 100% match) and/or microhomeology (> = 70% similar) from other studies [14, 20, 23, 31, 35] are shown in Additional file 1: Figure S15.

Based on junction sequencing results, ~ 9% of breakpoints coincided with LCRs/SegDups; PMD-LCRs were observed at ~ 7% of breakpoints, including LCRA1a (~ 1%), LCRA1b (~ 0.6%), LCRC (~ 3%), LCRD (~ 1%), LCR2 (~ 1%), and LCR3 (0.3%), while SegDups were observed at ~ 2% of breakpoints (Additional file 2: Table S3C). Additionally, ~ 2% of join-points mapped within a haploid reference genome “self-chain” region signifying an IR (Additional file 2: Table S3-C). Altogether, ~ 11% of sequenced *PLP1* breakpoints coincide with paralogous repeats. Nevertheless, this number may be an underestimate considering the high similarity of LCRs, in particular LCRA1a and LCRA1b, and the experimental limitation of obtaining sequence of the breakpoint junctions that coincide with them. Based on aCGH results, 37 breakpoints mapped to, and were likely mediated by, LCRA1a/LCRA1b (Additional file 2: Table S3-D).

Although LINE elements were present at 19% of join-points, LINE-LINE-mediated rearrangements (forming chimeric LINEs) are responsible for only ~ 2% (3/159) of join-points while evidence for *Alu*-*Alu*-mediated rearrangement (forming chimeric *Alus*) was found at ~ 8% (12/159) of join-points; the structure of different *Alu* family members can be conceptually considered as an ~ 300-bp track of microhomeology [29, 45]. In this study, we have not counted microhomology or microhomeology at join-points resulting from chimeric events between repetitive elements.

## Discussion

PMD is a rare X-linked disorder of the CNS with an estimated incidence of 1.9 per 100,000 male live births in the USA [47]. Genomic rearrangements leading to copy number gain of *PLP1* are the major cause of PMD, but the contribution of CGRs specifically in PMD is not well-established. Here we investigated genomic rearrangements in PMD in 50 male patients by high-resolution oligonucleotide-based aCGH or clinical chromosomal microarray analysis (CMA) and breakpoint junction sequence analysis. Among 50 unrelated individuals manifesting the PMD phenotype, 33 individuals (66%) were found to have single duplications within the Xq22 region, one of which was known to be an insertional translocation of the *PLP1* duplicated locus into chromosome Y [40]. By comparison, evidence for CGRs was observed in 17 individuals (34%).

Non-random grouping of the distal breakpoints into the LCR cluster was observed in 28/50 (56%) of individuals (Additional file 1: Figure S5), implicating a role for repeated sequences in genomic instability and generation of nonrecurrent genomic rearrangements, potentially by facilitating TS [26, 48–50]. In particular, the presence of highly identical LCRs, LCRA1a and LCRA1b mapping at the majority (59%) of the distal breakpoints in CGRs, further emphasizes the role of IRs in mediating or stimulating replication-based mechanisms (RBMs), especially in CGRs with higher-order amplifications [31]. Similar observation has been reported for the *MECP2* duplication syndrome at Xq28; e.g., 77% of the distal breakpoints group within a 215-kb genomic interval involving several LCRs/IR [50]. In another study involving individuals with the Yuan-Harel Lupski *PMP22-RAI1* contiguous gene duplication syndrome [YUHAL; MIM: 616652], proximal breakpoints in 33.33% of individuals were located within an LCR cluster [51].

In our study, LINEs were present in ~ 19% of breakpoints at the *PLP1* locus, but only one chimeric LINE was identified (BAB8920). In a recent study, 17,005 directly oriented LINE pairs (> 4 kb length and > 95% similarity) with the distance of less than 10 Mb have been identified, putting ~ 82.8% of the human genome at risk of LINE-LINE-mediated rearrangement [33]. However, based on our data, LINE pairs do not have a significant role in mediating genomic rearrangements at the *PLP1* locus.

Our results provide further evidence supporting the contention that RBMs play the predominant role in the generation of nonrecurrent structural variants. A collapsed DNA replication fork can result in a seDSB that upon further processing exposes a 3′ single-stranded DNA. The exposed single strand can then be utilized to prime synthesis on a template strand using either homology as provided by repetitive elements, e.g., *Alu* and LINE elements or microhomology at sites lacking long stretches of homology to reestablish a productive and processive replication fork (MMBIR) [22, 52]. Mutational signatures of replicative repair such as de novo SNVs and indels can be found flanking the breakpoint junctions and are features of RBM [3, 22, 23, 30]. MMBIR is proposed to be essential for the restarting of broken replication forks, but it appears to utilize DNA polymerases that are error prone [30, 52].

In our study, breakpoint junction complexities such as genomic insertions ranging from 1 to 959 bp were observed in several breakpoint junctions, including samples with array-based single duplications (Additional file 1: Figures S1-S4). These findings, in addition to the rearrangements being copy number gain events, are consistent with a replicative repair process where the polymerase acts with reduced processivity and hence undergoes one (small insertion) or multiple TS before forming a highly processive migrating replisome; establishment of this processive replisome perhaps signifies a switch to utilization of a different DNA polymerase. Therefore, both small (< 20 bp) and large insertions can result from multiple fork collapses and iterative strand invasions (Additional file 1: Figures S2 and S1–4 for individuals BAB8949 and BAB8950, respectively). Alternatively, small templated insertions can result from replication errors (Additional file 1: Figures S1–2 and S1–6, BAB8933 and BAB8966) and small non-templated insertions can arise potentially from MMEJ or NHEJ (random insertions; Additional file 1: Figures S1–3 to S1–6, BAB8946, BAB8951, BAB8963, and BAB8969).

Among 17 individuals with CGRs identified in this study, nine individuals showed interspersed duplications (Fig. 3a, and Additional file 1: Figures S6, S7 and S9). Three of these rearrangements could be either single duplications that occurred on the H2 haplotype or two duplications with one of two TSs involving reversal of the direction of replication between IRs LCRA1a and LCRA1b. Four rearrangements had directly oriented DUP-NML-DUP structures and two had DUP-NML-INV/DUP structures. We note a relatively large size interval for regions between duplications in individuals BAB8920, BAB8923, and BAB8934. Interestingly, one out of two breakpoint junctions in all three individuals appeared to be either LINE/LINE or *Alu*/*Alu* mediated. Highly identical SINE or LINE pairs at breakpoints can be mediating the underlying replicative mechanism by stimulating long-distance TS [33, 44]. The orientation of interspersed repeats appears as a determining factor for the overall rearrangement pattern observed wherein oppositely oriented LINEs or *Alu*s mediate a DUP-NML-INV/DUP rearrangement pattern while directly oriented *Alu*s mediate a DUP-NML-DUP pattern of rearrangement (Additional file 1: Figures S6–1 and S7) [37]. MMBIR is the most parsimonious mechanism to explain the presence of a second join-point within the same CGR event—reflecting iterative TS wherein the direction of replication is reversed when LINEs or *Alus* are oppositely oriented.

A rearrangement pattern consistent with DUP-TRP/INV-DUP was found in two individuals and suspected in a third (Fig. 3b and Additional file 1: Figure S10). This pattern of CGR was initially described at the *MECP2* locus in which unrelated individuals with complex duplication/triplication alterations indicated shared genomic architectural features [26]. Carvalho et al. also reported this pattern at the *PLP1* locus [26] and Beck et al. [31] reported it in 16 unrelated PMD individuals, providing further evidence that inverted LCRs facilitate the rearrangement formation. In our cohort, two out of three individuals with DUP-TRP/INV-DUP rearrangements share those genomic architectural features. Our results support the previously proposed two-step process in which the first TS occurs via BIR, mediated either by inverted LCRs or by inverted repetitive elements (such as *Alus*), reversing the direction of replication, and the second TS, which restores the original direction of replication, occurs via MMBIR [26, 37]. Exception was found in individual BAB8938 with a DUP-TRP/INV-DUP rearrangement who showed a unique architectural feature with no evidence for IRs being involved, at least from examining the haploid reference genome. Also, in this case, the triplicated segment is inverted. This finding supports previous observations that the involvement of inverted LCRs is perhaps not a fundamental requirement for the generation of DUP-TRP/INV-DUP rearrangement. Inverted LCRs are relevant to the majority of these events described thus far [31, 53]; alternatively, a repetitive or short repeat sequence may occur in that subjects’ personal genome that differs from the consensus haploid reference human genome build.

A very rare CGR involving a quadruplicated genomic segment distal to *PLP1* was observed in individual BAB8937 (DUP-QUAD-TRP) (Fig. 3c and Additional file 1: Figure S11). A CGR with the same pattern, but with a quadruplicated segment proximal to *PLP1*, has been previously reported [31]. In such CGRs, probably three breakpoints are present in which two breakpoints are identical [31]. MMBIR can most parsimoniously explain this copy number amplification event through a rolling-circle model [22, 31]. In higher-order amplification rearrangements, the clinical phenotype can be more severe if triplication or quadruplication includes the dosage-sensitive gene(s) [24, 26, 54]. 

In this cohort, we found three individuals with more than two duplications separated by CNRs (BAB8924, BAB8936, and BAB8959, Fig. 3c and Additional file 1: Figure S12). There are two possible explanations for the appearance of such CNVs. These CNRs can be deletion products in hotspot regions of the human genome. Genomic rearrangement with interchromosomal TS during oogenesis can potentially explain the presence of such genomic rearrangements in some cases, although a SNP array performed on BAB8959 did not support this hypothesis (Additional file 1: Figure S12–2). However, we could not exclude the presence of a copy number neutral absence of heterozygosity (AOH) region involving the CNV in BAB8959. Another possibility is the coincidence of three independent genomic rearrangement events including two deletions and one intrachromosomal duplication during gametogenesis or early embryogenesis. For BAB8936, we do not know if the two small CNRs are inherited or related to the formation of the CGR (Additional file 1: Figure S12–1b). However, based on the genomic position of the CNRs in UCSC Genome Browser (GRCh37/hg19), it is unlikely that they are due to rearrangements mediated by repeats or repetitive elements.

We found multiple breakpoint junction sequences showing microhomeology. The aggregate results of breakpoint junctions and surrounding genomic sequence suggest that not only a higher similarity at the junctions, represented by either a microhomology or microhomeology, is facilitative, but also a higher sequence complementarity of the surrounding regions could potentially contribute to the TS during the DNA replicative repair process. To gain insight into the frequencies and distribution of RBM mutational signatures at different rearrangement join-points, we performed a meta-analysis of all published breakpoint sequences from genomic rearrangements with *PLP1* gain events in association with PMD. We combined our data with six other studies, all but one of which used the same genomic assay: oligonucleotide array-based CGH (Fig. 5) [14, 20, 23, 31, 34, 35]. In total, from 134 individuals with PMD studied, single duplications were found in ~ 55% of individuals. Remarkably, among all CGR cases, triplication flanked by duplications is the most frequent CGR, ~ 20% of all PMD individuals, ~ 44% among all PMD individuals with CGRs. In total, ~ 15% of rearrangements showed two duplications separated by a CNR (Additional file 2: Table S3). Examination of the level of base pair similarity near breakpoints suggests that TS was mediated by microhomology/microhomeology in ~ 54% (Table 2), and repetitive sequences (*Alu* and LINE1) in ~ 9% of all cases. Interestingly, although we did not calculate microhomology and microhomeology in chimeric elements for this study, *Alu*-*Alu*-mediated rearrangements, when resulting in chimeric elements with substrate pairs between different family members, can potentially be microhomeology-mediated TS rather than NAHR [29, 45]. Of note, *Alu* elements are much shorter than LCRs and LINE elements, and different *Alu* families may not contain enough homology for NAHR [28, 45]. Here, for the first time, we provide robust experimental evidence for microhomeology as a mutational signature at breakpoint junctions at the *PLP1* locus. Moreover, our computational analyses of microhomology and microhomeology support the donor-acceptor hypothesis [29] wherein microhomology facilitates W-C base pair complementarity and strand annealing to prime DNA replication during TSs.

## Conclusions

This study extends our knowledge about the distribution of genomic rearrangements with copy number gains at the *PLP1* locus, their underlying molecular mechanisms, and potential mutational signatures accompanying structural variant mutagenesis. Importantly, CGRs occur in ~ 45% of all rearrangements involving this locus. We provide evidence for the role of microhomeology in genomic rearrangements at the *PLP1* locus, perhaps facilitating TS, and thus, it may be considered a mutational signature of MMBIR. This strongly supports the role of FoSTeS/MMBIR, as microhomology/microhomeology-mediated TS, as the driving mechanism leading to the generation of nonrecurrent rearrangements at the *PLP1* locus.

## Supplementary information

**Additional file 1: Figure S1.** (S1-1 to S1-6). aCGH and breakpoint junction sequencing results for 30 of the 33 PMD individuals with single duplications at the *PLP1* locus. **Figure S2.** Breakpoint junction sequencing in subject BAB8949 with a single duplication revealed insertions with multiple join-points at the breakpoint junction. **Figure S3.** Replication errors at the breakpoint junction and/or flanking regions in BAB8929. **Figure S4.** The aCGH result for BAB8921 showed a 666 Kb single duplication at the *PLP1* locus. **Figure S5.** The distal breakpoint junction points of genomic rearrangements in 28 PMD subjects are grouped within the LCR distal of *PLP1*. **Figure S6.** (S6-1 to S6-4). Breakpoint junction analysis indicates that three patients have a directly oriented DUP-NML-DUP pattern of rearrangement. **Figure S7.** (S7-1 and S7-2). Breakpoint junction analysis indicates that two patients have a DUP–NML–INV/DUP pattern of rearrangement. **Figure S8.** Three possible rearrangements for the generation of DUP–NML–INV/DUP structures satisfy the breakpoint junctions that we obtained on patients BAB8920 and BAB8934. **Figure S9.** Three individuals with a DUP-NML-DUP pattern on aCGH (BAB8940, BAB8955, and BAB8960) have the distal duplication and copy neutral region between the two duplications mapping within IRs LCRA1a to LCRA1b. **Figure S10.** (S10-1 to S10-3). CGRs with DUP-TRP-DUP pattern of rearrangement on aCGH. **Figure S11.** The most complex rearrangement in this study, DUP-TRP-QUAD, was observed in individual BAB8937. **Figure S12.** (S12-1 and S12-2). Samples with DUP-NML-DUP-NML-DUP pattern of rearrangement (based on aCGH). **Figure S13.** One individual, BAB8931, exhibited DUP-NML-DEL pattern of rearrangement. **Figure S14.** The sequence similarity comparison of reference sequences surrounding join-points. **Figure S15.** Similarity comparisons of reference sequences surrounding join-points were done after re-analyzing of break-point junction sequences by a retrospective study.

**Additional file 2: Table S1.** Samples with single duplications at the *PLP1* locus. **Table S2.** A summary of genomic rearrangements, coordinates and breakpoint junctions in the cohort of 50 PMD patients. **Table S3.** Original data from 7 studies on genomic rearrangements at the *PLP1* locus. **Table S4.** Microhomeologous sequences at the join-points found in this study. **Table S5.** Other features at the join-points found in this study. **Table S6.** Microhomeologous sequences at the join-points found by re-analyzing breakpoint sequences from previous studies.

## Data Availability

The aCGH data have been deposited in NCBI’s Gene Expression Omnibus [55] and are accessible through GEO Series accession number GSE138542 (https://www.ncbi.nlm.nih.gov/geo/query/acc.cgi?acc=GSE138542).

## Abbreviations

aCGH: Array comparative genomic hybridization
BIR: Break-induced replication
CGRs: Complex genomic rearrangements
CMA: Chromosomal microarray analysis
CNR: Copy neutral region
DGV: Database of genomic variants
FISH: Fluorescent in situ hybridization
FoSTeS: Fork Stalling and Template Switching
HR: Homologous recombination
IR: Inverted repeat
LCR: Low copy repeat
LINE: Long interspersed nuclear elements
MMBIR: Microhomology-mediated break-induced replication
MMEJ: Microhomology-mediated end joining
NAHR: Non-allelic homologous, recombination
NHEJ: Non-homologous end joining
PLP1: Proteolipid protein 1
PMD: Pelizaeus Merzbacher disease
RBMs: Replication-based mechanisms
SegDup: Single-ended, double-stranded DNA break
SNP: Single nucleotide polymorphism
SNV: Single nucleotide variants.
