# Supplementary material for: Distinct patterns of complex rearrangements and a mutational signature of microhomeology are frequently observed in PLP1 copy number gain structural variants
Source: Genome Med. 2019 Dec 9;11:80. doi: 10.1186/s13073-019-0676-0 (PMC6902434; doi:10.1186/s13073-019-0676-0)

BAB8922

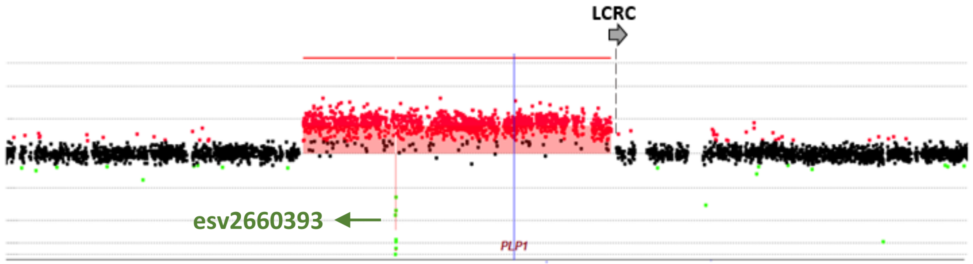

X (+) 103188753-794 TACTCTCCACCACCTTGAGATCAACTTTTTAGCTCTCCCAT

BAB8922 TACTCTCCACCACCTTGAGATCTTCCTAGCCTCAGTGGTCTT

X (+) 102713539-580 GCTCATTAGTTGATGCAGTTTCTTCCTAGCCTCAGTGGTCTT

BAB8925

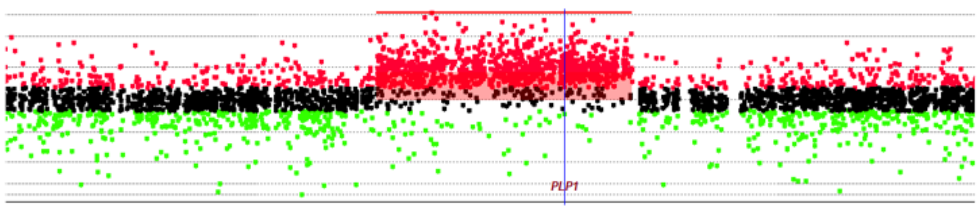

Not available

BAB8926

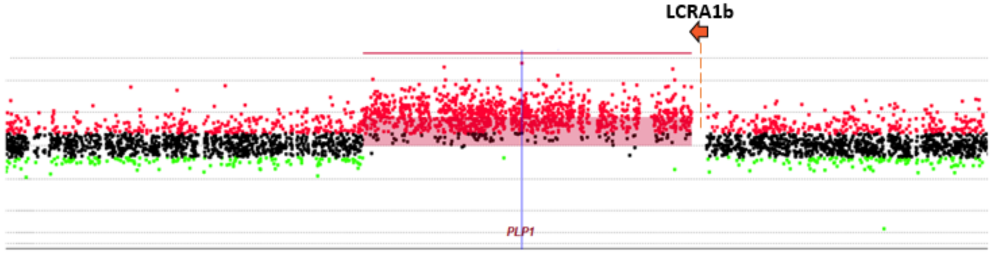

X (+) 103322048-089 GATAGAGATTTAGGTAACCCGACTTGGTTGGGTTACAGTATC

BAB8926 GATAGAGATTTAGGTAACCCGAGCTCAGATATCAATCACAGC

X (+) 102793954-995 TGTTTTTGAACTCACCTGGTGAGCTCAGATATCAATCACAGC

BAB8930

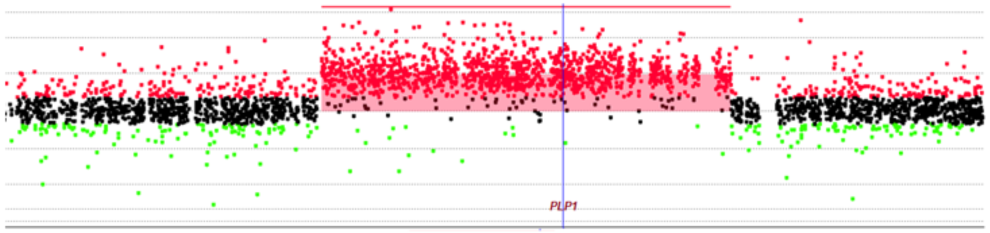

8/10 (80 %)

X (+) 103267038-087 AAACCTGGAAGTAACGAAGGTTTCTGTTTAAAAAGAAACGAAAAGATTAT

BAB8930 AAACCTGGAAGTAACGAAGGTTTCTGTTGATTGGAATAGTTTCGGAAGGA

X (+) 102711998-047 GTTAGGGAGGATTCTCTCTTTTTCTATTGATTGGAATAGTTTCGGAAGGA

BAB8932

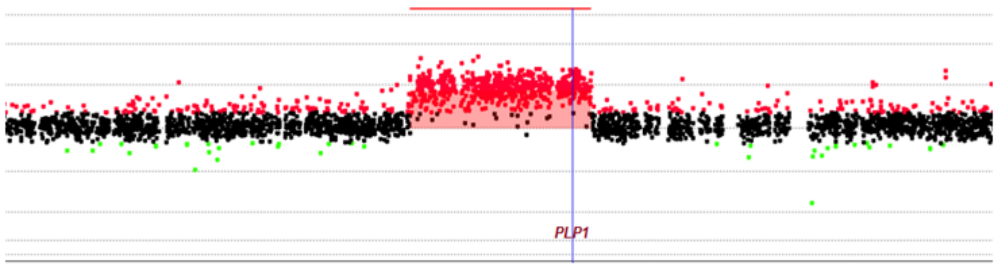

Not available

Fig S1-1

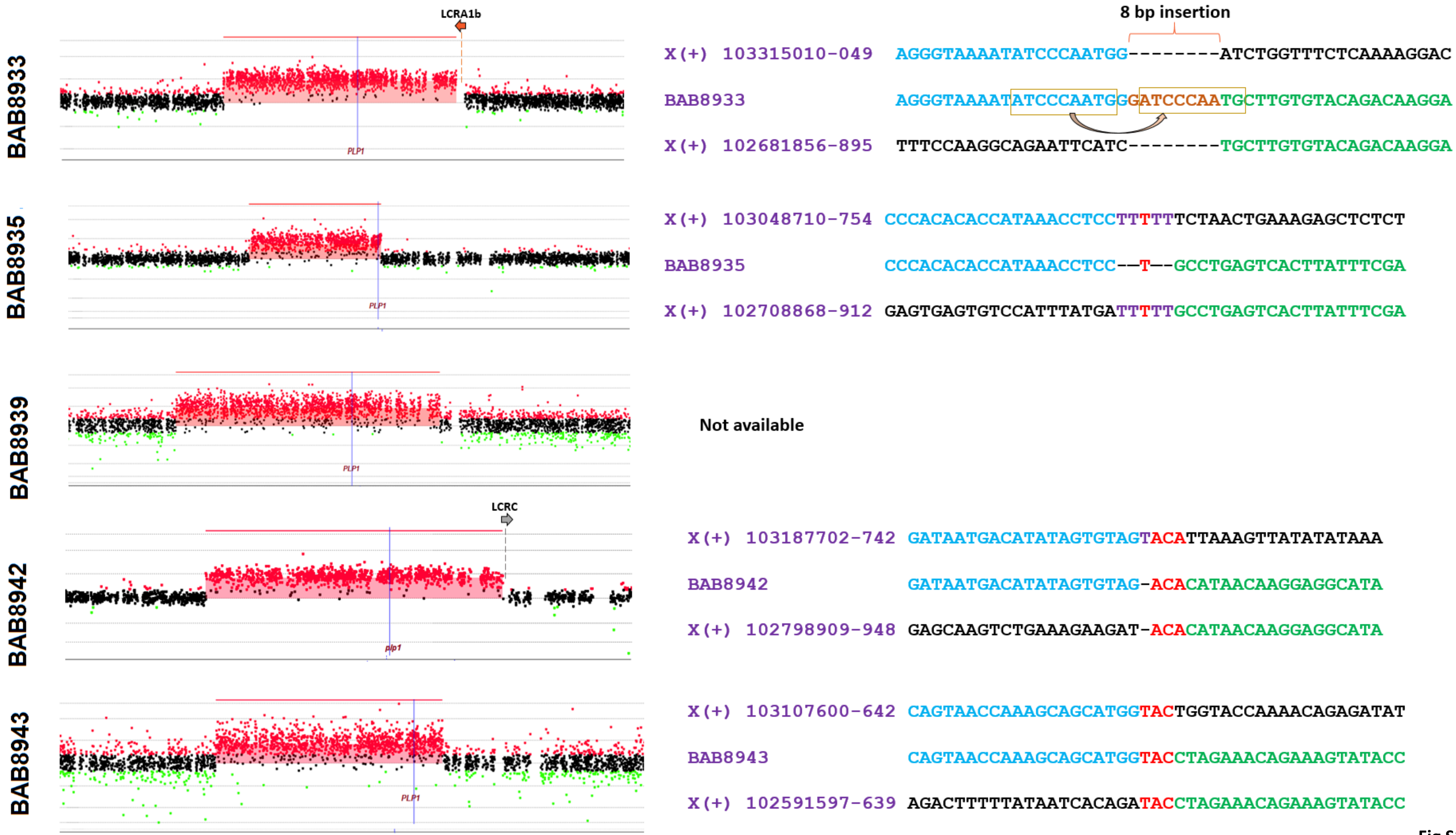
BAB8933
LCRA1b
PLP1
8 bp insertion
X(+) 103315010-049 AGGGTAAAATATCCCAATGG--------ATCTGGTTTCTCAAAAGGAC
BAB8933 AGGGTAAAATATCCCAATGGGATCCCAATGCTTGTGTACAGACAAGGA
X(+) 102681856-895 TTTCCAAGGCAGAATTCATC--------TGCTTGTGTACAGACAAGGA
BAB8935
PLP1
X(+) 103048710-754 CCCACACACCATAAACCTCCTTTTTTCTAACTGAAAGAGCTCTCT
BAB8935 CCCACACACCATAAACCTCC--T--GCCTGAGTCACTTATTTCGA
X(+) 102708868-912 GAGTGAGTGTCCATTTATGATTTTTGCCTGAGTCACTTATTTCGA
BAB8939
PLP1
Not available
BAB8942
LCRC
plp1
X(+) 103187702-742 GATAATGACATATAGTGTAGTACATTAAAGTTATATATAAA
BAB8942 GATAATGACATATAGTGTAG-ACACATAACAAGGAGGCATA
X(+) 102798909-948 GAGCAAGTCTGAAAGAAGAT-ACACATAACAAGGAGGCATA
BAB8943
PLP1
X(+) 103107600-642 CAGTAACCAAAGCAGCATGGTACTGGTACCAAAACAGAGATAT
BAB8943 CAGTAACCAAAGCAGCATGGTACCTAGAAACAGAAAGTATACC
X(+) 102591597-639 AGACTTTTTATAATCACAGATACCTAGAAACAGAAAGTATACC

Fig S1-2

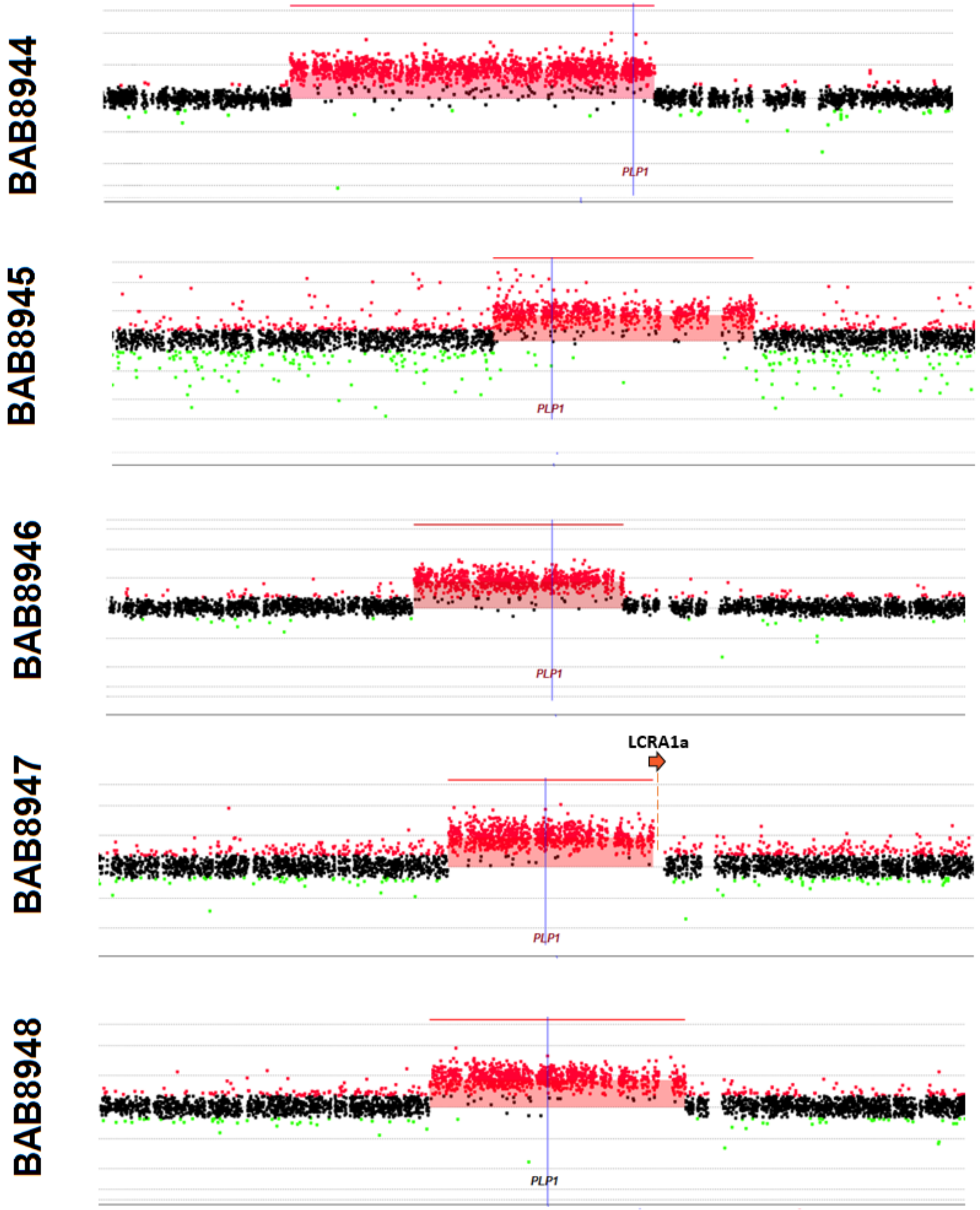

10/14 (71 %)

X(+) 103073616-657 ATATATATTAAGTAAAAAGATT-ACAGTGTGTGCAGTATCAA

BAB8944 ATATATATTAAGTAAAAAGATTGACATGCATAATTGTTATGA

X(+) 102511190-232 TGTGTCCCATTAAAAGAACTATTGACATGCATAATTGTTATGA

X(+) 103382317-359 CCCTGAACCAACCAATCAGAGCTCATCTTCCTCAGCCAATCAG

BAB8945 CCCTGAACCAACCAATCAGAGCTGTGTTTGCAAGTGAGGGTCG

X(+) 102942229-271 AAAGGCGCAGTGGGGTTCTGCTGTGTTTGCAAGTGAGGGTCG

3 bp insertion

X(+) 103163803-842 ATTAGCTCATTTCAAGAAAA---AAAACCCTAAAAATTAGTTT

BAB8946 ATTAGCTCATTTCAAGAAAATTGATGAATTCAGTAAAACTGTG

X(+) 102801929-968 AAAACTGTTGGAATTGATGA---ATGAATTCAGTAAAACTGTG

7/10 (70 %)

X(+) 103243066-110 AGAATTCTTGTGATTTAGGTCTTAAAAATATAGACATGTATTCCC

BAB8947 AGAATTCTTGTGATTTAGGTCTTAAGAACATGGTGCCAGCAGATT

X(+) 102875625-669 ACAGCTCTGGAGGCTGGAAGTTCAAGAACATGGTGCCAGCAGATT

Common SNP: Rs578953 (G>A)

X(+) 103268320-364 ACTGGATGGTCCCGTTGCGTGGCATCTCGTCAACCAATCACAGTG

BAB8948 ACTGGATGGTCCCATTGCGTGGCATAGAGTTGTTCATATTAACTT

X(+) 102845345-389 TCTATATTAACAAATTTGTAGGCATAGAGTTGTTCATATTAACTT

Fig S1-3

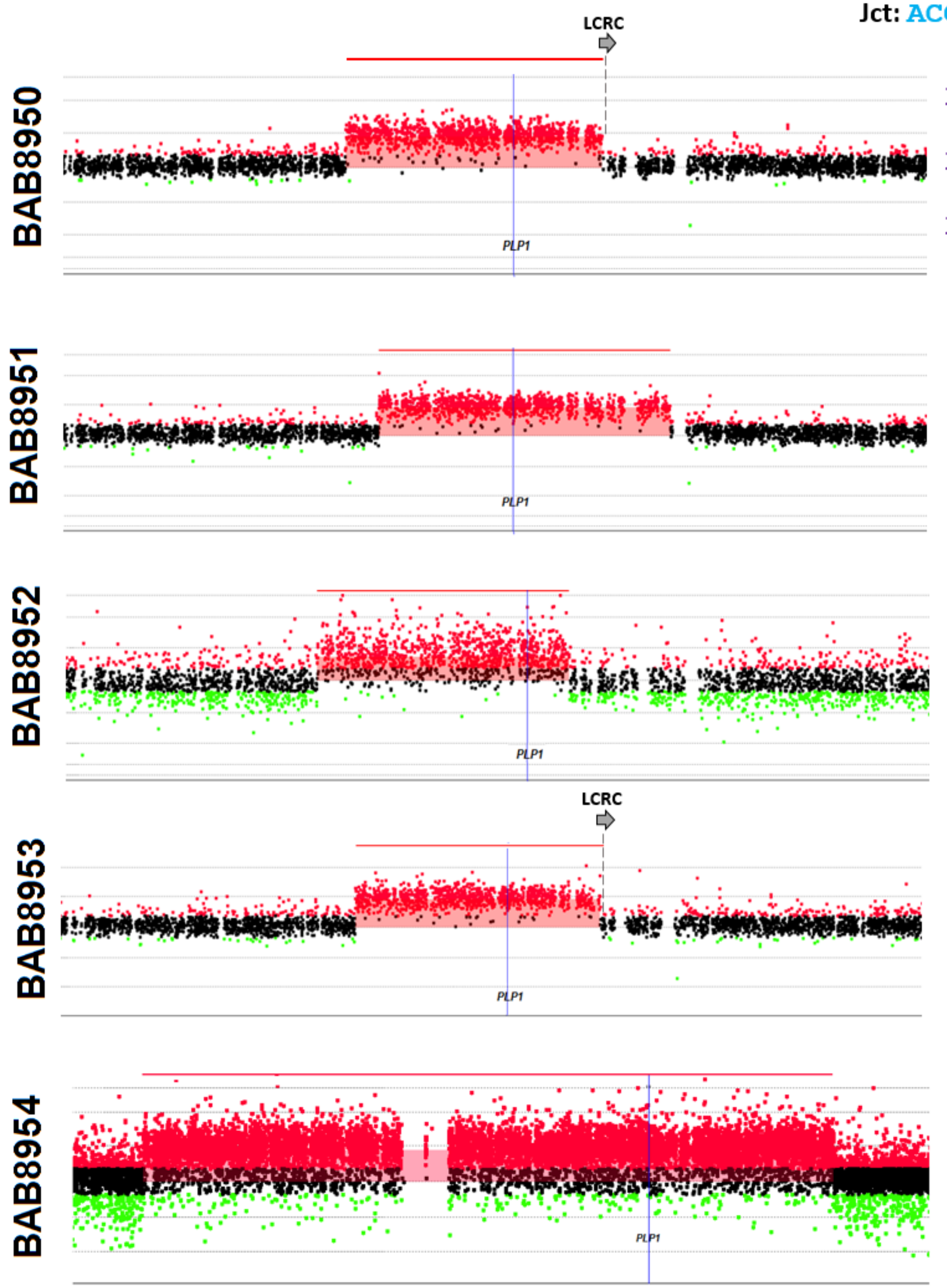

Jct: ACCTAGTCCTCAGCTTGTGGGATATATCCCCCCTCAGCCCTTTAAATGCTGAGACACAG

11 bp insertion resulted from TS: ATCCCCCCTCA

6/8 (75 %)

X (+) 103186844-886 CCTAGTCCTCAGCTTGTGGGAT-ATGACGCTATCTCCAGGTAGA

X (-) 103026459-517 TTGTAGGTAGCCAAATTGGGTTCATATCCCCCCTCAGCCTGGGACATAGCACAGTAAAT

X (+) 102757807-850 GCATGAAGTGGGACTTCAATGGCCCTTTAAATGCTGAGACACAG

2 bp insertion

X(+) 103301537-576 CACCTTGTGAAATCTATTTT--CCTCCTCTTTGGAGACACCT

BAB8951 CACCTTGTGAAATCTATTTTGTCTTTAGCAAATATATCAGGA

X(+) 102812934-973 TTTATTGGCTAATTGCATAG--CTTTAGCAAATATATCAGGA

Rare SNV, Rs765589952 (T>C)

X(+) 103109709-751 TAAAGGAAAGCATGTAAAGTCCCCGGTACATGAAAGGCACATA

BAB8952 TAAAGGAAAGCATGTAAAGCCCCTTCTATGGGGCTTCTAACTA

X(+) 102682357-399 ATCTGAGGTCAGGAGTCCTGCCCTTCTATGGGGCTTCTAACTA

10/13 (77 %)

X (+) 103199113-156 TGGAGGACATAGTAGCTGGGTTTGG-GAAGATAGTTTGGGACAAT

BAB8953 TGGAGGACATAGTAGCTGGGTTTGGAGAACCCTAGGGAAACAACT

X (+) 102773637-681 ATATCAACTCATTGCTTGCCTTTGGAGAACCCTAGGGAAACAACT

X(+) 104246617-658 GTGAACAGAAATCAAAATCTAGTTAGGTTGGTAATTGGGACC

BAB8954 GTGAACAGAAATCAAAATCTAGCTTCCGGAGTAGCTGGGACT

X(+) 99762660-701 TTACGCCATTCTCCTGCCTCAGCTTCCGGAGTAGCTGGGACT

Fig S1-4

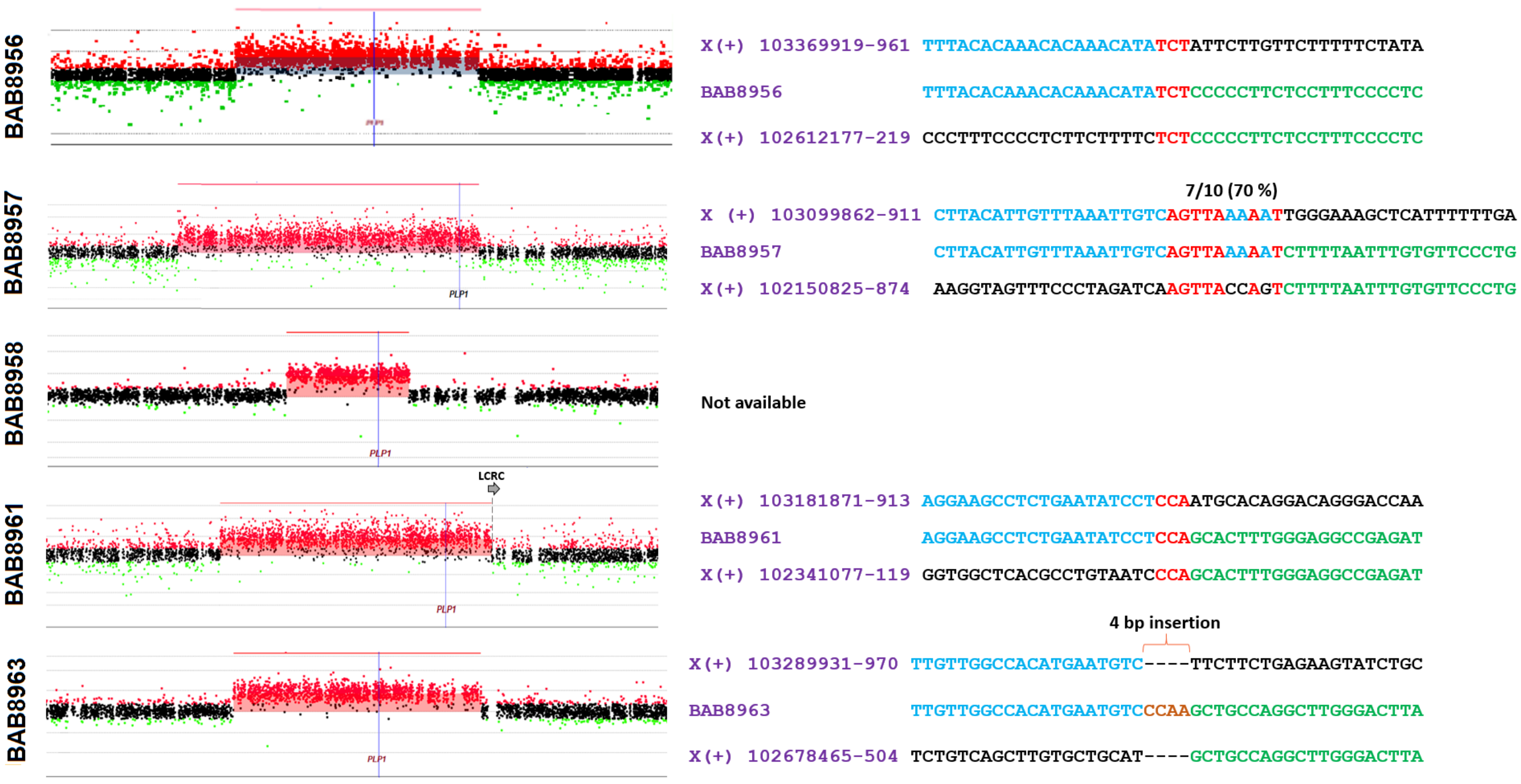
BAB8956
X(+) 103369919-961 TTTACACAAACACAAACATATCTATTCTTGTTCTTTTTCTATA
BAB8956 TTTACACAAACACAAACATATCTCCCCCTTCTCCTTTCCCCTC
X(+) 102612177-219 CCCTTTCCCCTCTTCTTTTCTCTCCCCCTTCTCCTTTCCCCTC
BAB8957
PLP1
7/10 (70 %)
X (+) 103099862-911 CTTACATTGTTTAAATTGTCAGTTAAAAATTGGGAAAGCTCATTTTTTGA
BAB8957 CTTACATTGTTTAAATTGTCAGTTAAAAATCTTTTAATTTGTGTTCCCTG
X(+) 102150825-874 AAGGTAGTTTCCCTAGATCAAGTTACCAGTCTTTTAATTTGTGTTCCCTG
BAB8958
PLP1
Not available
BAB8961
LCRC
PLP1
X(+) 103181871-913 AGGAAGCCTCTGAATATCCTCCAATGCACAGGACAGGGACCAA
BAB8961 AGGAAGCCTCTGAATATCCTCCAGCACTTTGGGAGGCCGAGAT
X(+) 102341077-119 GGTGGCTCACGCCTGTAATCCCAGCACTTTGGGAGGCCGAGAT
BAB8963
PLP1
4 bp insertion
X(+) 103289931-970 TTGTTGGCCACATGAATGTC----TTCTTCTGAGAAGTATCTGC
BAB8963 TTGTTGGCCACATGAATGTCCCAAGCTGCCAGGCTTGGGACTTA
X(+) 102678465-504 TCTGTCAGCTTGTGCTGCAT----GCTGCCAGGCTTGGGACTTA

Fig S1-5

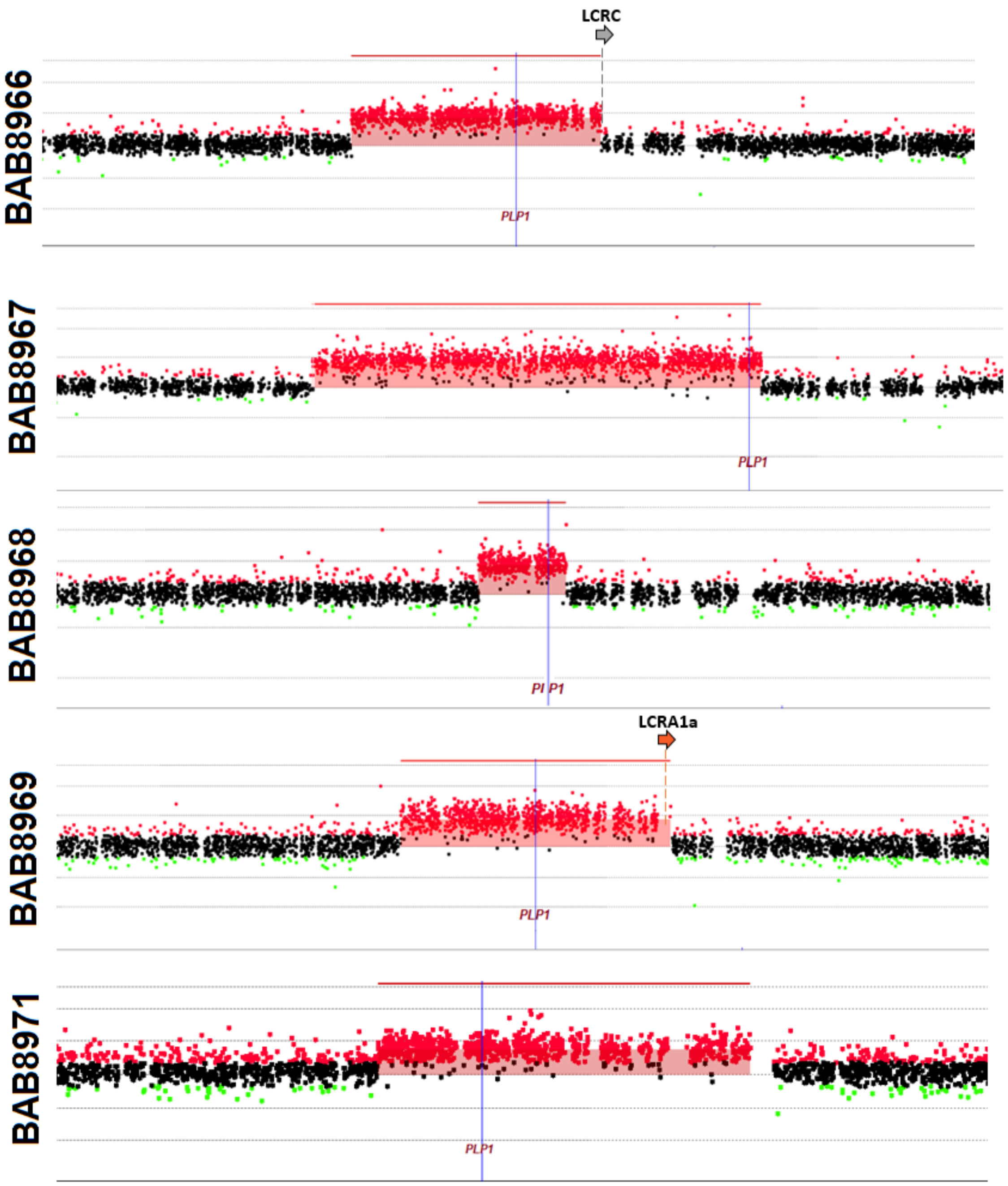

8 bp insertion

X(+) 103175437-476 CATGG-AAACAGCATGCAGGG--------CTGTCCAAAGTGAATGGCCA

BAB8966 CATGGAAAACAGCATGCAGGGGGGGACTGCTGGGGACAGGGCAATAGGA

X(+) 102778226-265 TGACA-AAGTATTGTGTTTGT--------CTGGGGACAGGGCAATAGGA

7/10 (70 %)

X (+) 103057046-087 CAATATTAAATGGATCGAAGTGGGTGCCTGAGGTGAGAGAGG

BAB8967 CAATATTAAATGGATCGAAGTGCTTTAAAGGCAAATAAAGA

X (+) 102364332-371 GTAGGTGACAAAGATC-ACATGCTTTAAAGGCAAATAAAGA

Not available

2 bp insertion

X(+) 103243760-799 ACACCAAATACGTGAATCCG--ACTCCTAACCTCTCATCTCT

BAB8969 ACACCAAATACGTGAATCCGTATGACGTGAGGAAGCTAGAGG

X(+) 102839709-748 ACGCTCTGATTGGATGTTGT--TGACGTGAGGAAGCTAGAGG

Not available

Fig S1-6

Fig S1. aCGH and breakpoint junction sequencing results for 30 of the 33 PMD individuals with single duplications at the *PLP1* locus. The breakpoint junctions for 24 of the 30 individuals shown here indicate that the duplications are tandem and head-to-tail. We were not able to resolve breakpoint junctions of the remaining six individuals. The three additional individuals with single duplications, BAB8949, BAB8929, and BAB8921, are shown in Figures S2, S3, and S4. The small deletion within the duplication in BAB8922 is a common polymorphism which has been previously reported in normal controls from African ancestry in the DGV database (esv2660393; 8/1151). Based on the genomic position of the deletion in UCSC Genome Browser (GRCh37/hg19), it is unlikely to be mediated by repeats or repetitive elements. Among 30 individuals shown here, replication errors including small insertions (BAB8933, BAB8946, BAB8951, BAB8963, BAB8966, and BAB8969), small deletions (BAB8935 and BAB8942), and a very rare single nucleotide variant (BAB8952; Rs765589952) with ~ 0.00005 allele frequency in the Genome Aggregation Database (gnomAD) were noted at breakpoint junctions and/or flanking regions. The G>A change (Rs578953) seen in BAB8948 is a common SNP with the allele frequency of ~0.17 in gnomAD). The spaces that are devoid of signal on the array of individual BAB8954 are due to a large LCR and consequent lack of uniquely mapping array probes used or interrogating copy number states. LCRA1a, LCRA1b, and LCRC are indicated on the arrays by arrows for individuals whose breakpoints map in these elements. Light brown rectangles and curved arrows in BAB8933 and BAB8966 show where insertions are possibly templated. Color code for nucleotides: blue for the distal end of duplication, green for the proximal end of duplication, red for microhomology or microhomeology, light brown for insertion, purple for single nucleotide variant (SNV) or deletion.

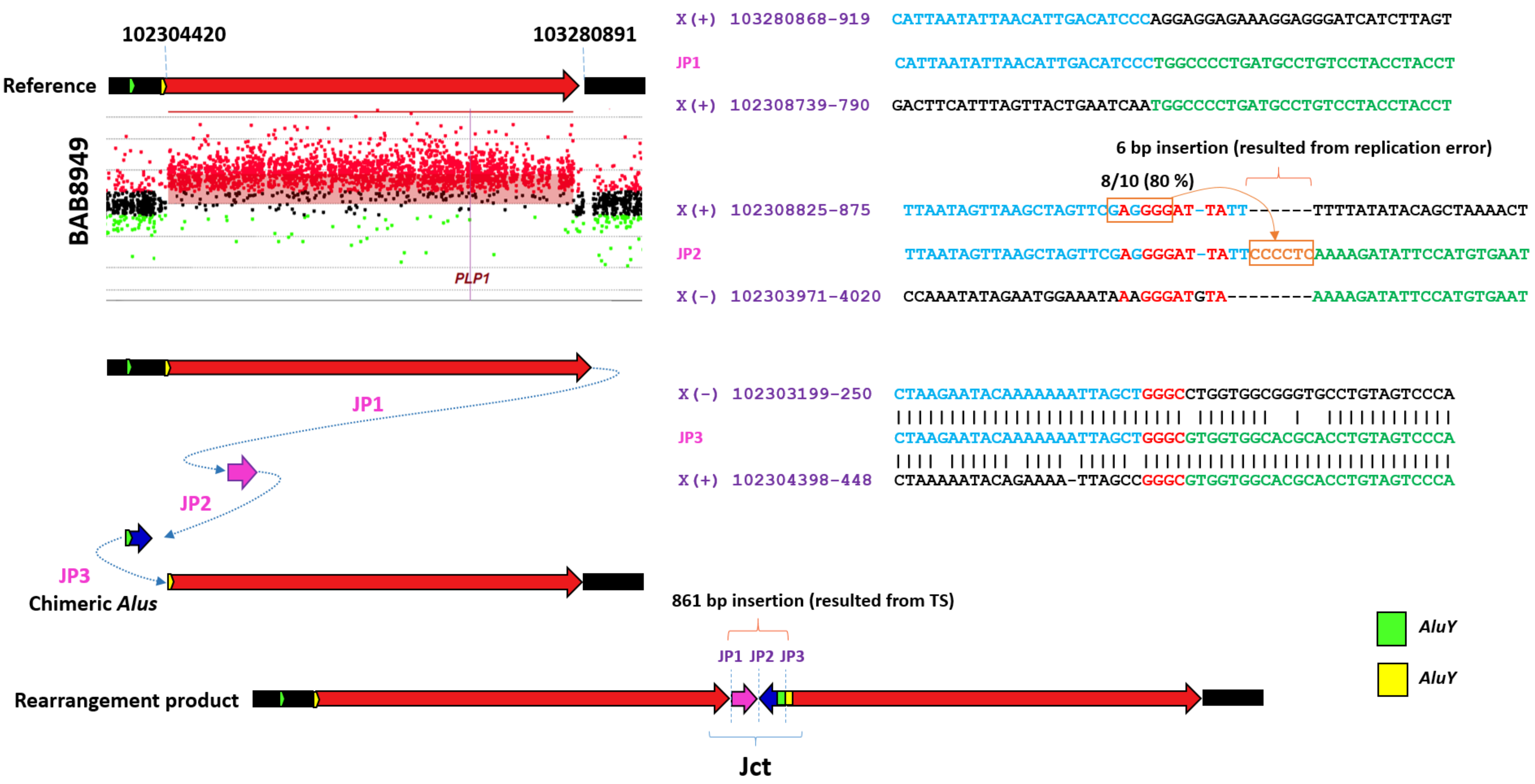

Fig S2. Breakpoint junction sequencing in subject BAB8949 with a single duplication revealed insertions with multiple join-points at the breakpoint junction. Among all samples with single duplications and sequenced breakpoint junctions, this was the only sample with a large insertion that resulted from three TSs. In this subject, TSs have occurred in which the second TS forming join-point 2 (JP2), is microhomeology-mediated, and the third TS (JP3) is *Alu-Alu* mediated, in which *AluY* (yellow) has been used as a substrate for an *Alu-Alu* (yellow-green)-mediated rearrangement forming a chimeric *AluY/AluY* (88% identity) at the join-point. Bases in light brown rectangles show where the insertion of sequence at JP2 is likely templated. Coordinates are based on breakpoint junction analysis. Color code for nucleotides: blue for the distal end of duplication, green for the proximal end of duplication, red for microhomology/microhomeology, light brown for insertion. Jct: Breakpoint Junction, JP: join-point.

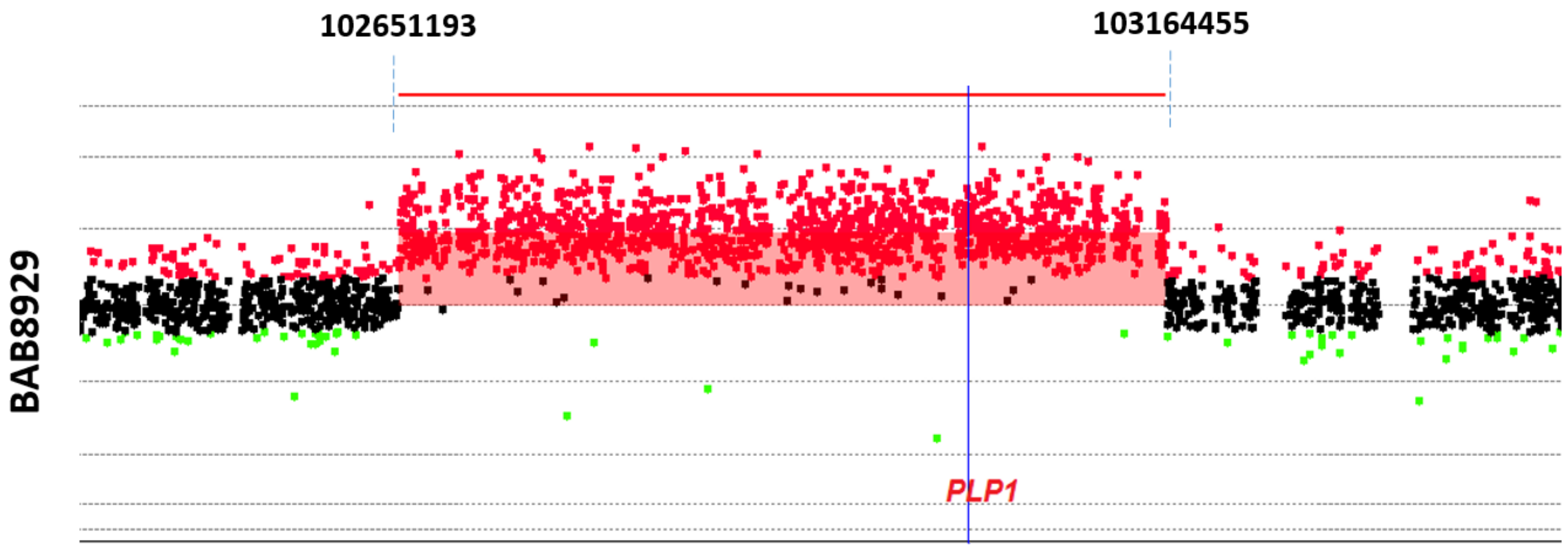

7 bp insertion

```
X (+): 103164435-536 CACTAGGTTTTCTGTATTCTG-------TGTACTCGTCATTCATGAATATTTTCATTTTTGACAACTAATTACTGAGAAAATTAATAGGGGGATTAAAATCATGACTCA
BAB8929              CACTAGGTTTTCTGTATTCTGGCATGGTAGGCAGGAAAGGGTGAA-------TGTGAATACATTTGAGGGGGTGGCATGATCTAA-GGGCATGTGGAGAGAAG-----A
X (+): 102651172-273 TTTCCTGGCTAGTAGTGGAGG-------AGGCAGGAAAGGGTGAACTACAAATGTGAATACATTTGAGGGGGTGGCATGATCTAAAGGGCATGTGGAGAGAAGAAAAAA
```

7 bp deletion 1 bp deletion 5 bp deletion

Fig S3. Replication errors at the breakpoint junction and/or flanking regions in BAB8929. In this case with a single tandem duplication, a small insertion at the breakpoint junction and three small deletions in the flanking region are likely the result of replication errors. Coordinates are based on breakpoint junction analysis. Color code for nucleotides: blue for the distal end of duplication, green for the proximal end of duplication, light brown for insertion, purple for deletion.

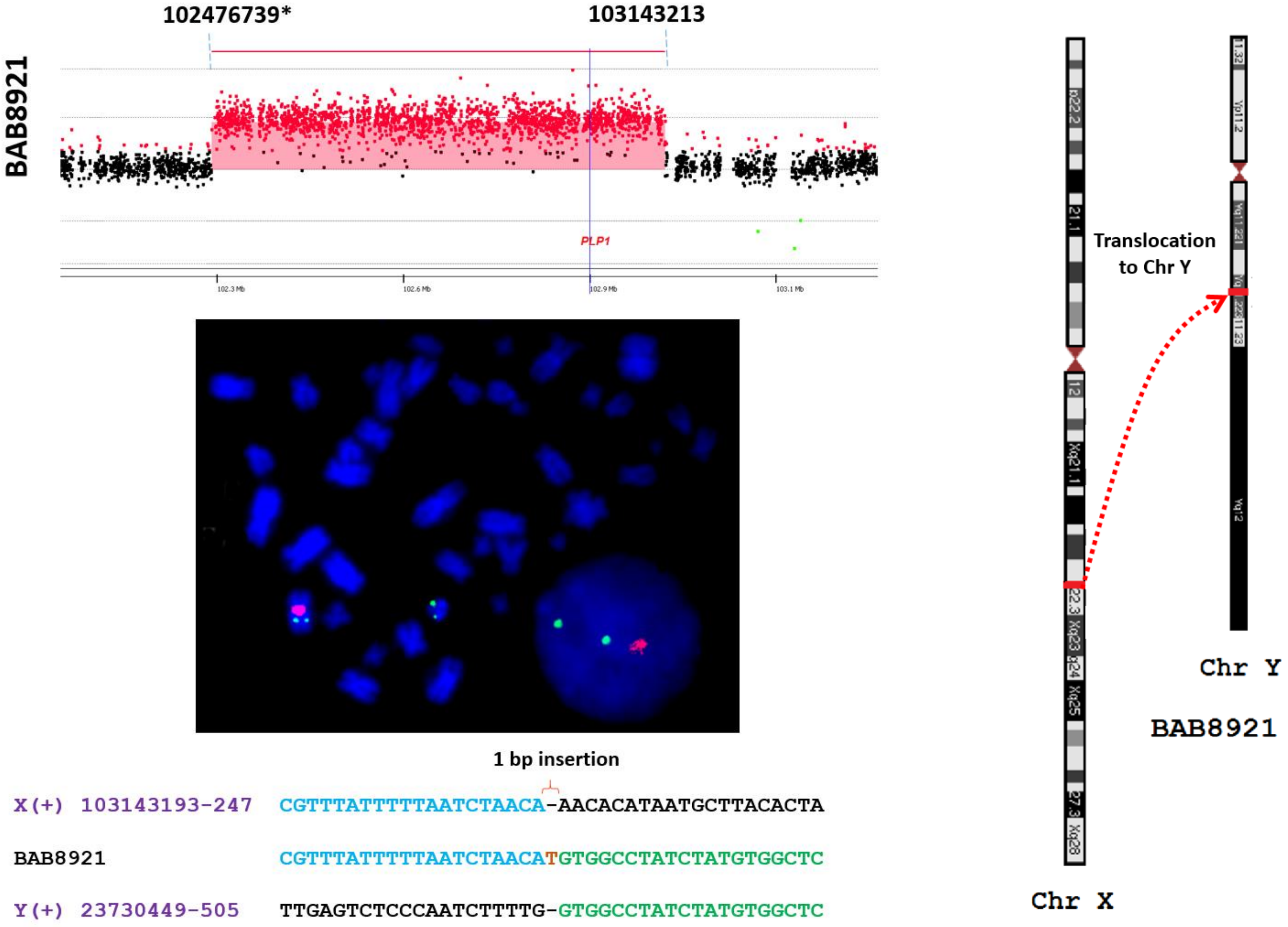

Fig S4. The aCGH result for BAB8921 showed a 666 Kb single duplication at the *PLP1* locus. Further study by interphase and metaphase FISH indicated an insertional translocation of the *PLP1* locus into Yq. A representative metaphase spread and interphase nucleus are shown. In the interphase nucleus, two green signals from the *PLP1* probe indicate duplication of *PLP1*, and a single red signal from the X centromeric probe indicates one X chromosome. Metaphase FISH shows the locations of the *PLP1* on chromosome Xq and Yq. Whole genome aCGH indicated that there was a duplication on Yq (coordinates: ~23,735,612-23,790,612 and 23,840,612-23,885,612; GRCh37/hg19). We were able to resolve one of the two breakpoint junctions for the insertional translocation, indicating that the distal end of the *PLP1* duplication was insertionally translocated to a position at the proximal end of the proximal duplication on chromosome Y. Coordinates are based on breakpoint junction analysis where possible. The one based on aCGH is marked by *. Color code for nucleotides: blue for the distal end of inserted segment, green for one end of break on ChrY, one base pair insertion at the join-point is shown with light brown.

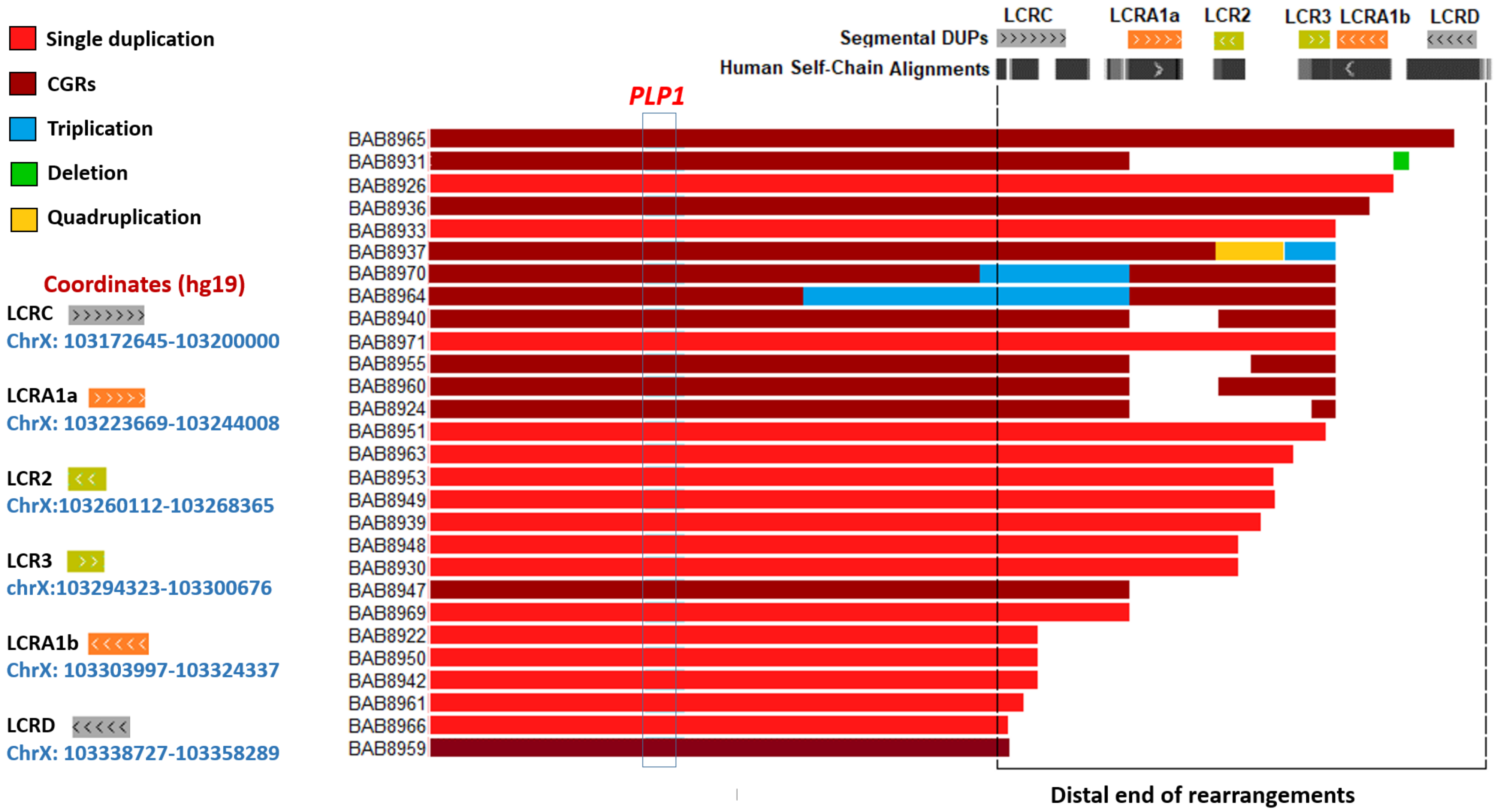

Fig S5. The distal breakpoint junction points of genomic rearrangements in 28 PMD subjects are grouped within the LCR distal of *PLP1*. LCR blocks and self-chain alignments are shown above the schematic diagram of the array results in the region on chromosome X from 103172645 to 103358289 (GRCh37/hg19). Approximate positions of breakpoints were determined by aCGH data and then the UCSC genome browser (GRCh37/hg19) was used to align genomic rearrangements. The position of the *PLP1* locus is highlighted with a light blue rectangle. Rearrangements are depicted as follows: duplicated genomic segments in red, triplications in blue, quadruplication in yellow, and deletion in green. LCRC and LCRD (gray) have 90 - 98% similarity and LCRA1a and LCRA1b (orange) have greater than 98% similarity. LCR2 and LCR3 (olive green) have 88% similarity with each other, LCR2 (olive green on left) has 87% similarity with LCRA1a and LCRA1b (orange), and LCR 3 (olive green on right) has 91% similarity with LCRA1a and LCRA1b (orange). Arrows on LCR blocks indicate the relative orientation of each LCR pair. In the UCSC genome browser (GRCh37/hg19), LCRs 2 and 3 can be found in the self-chain track but not segmental duplication track.

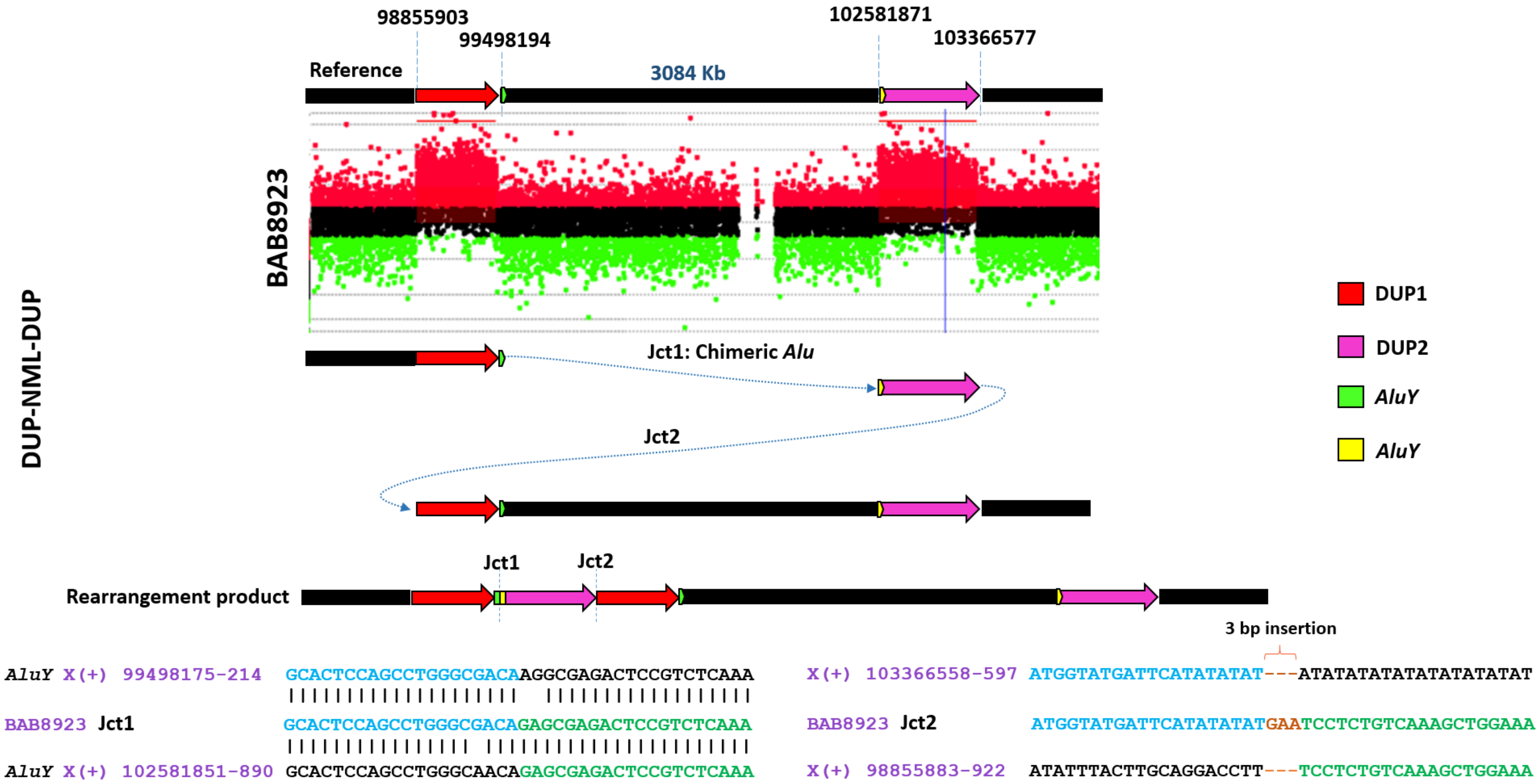

Fig S6. Breakpoint junction analysis indicates that three patients have a directly oriented DUP–NML–DUP pattern of rearrangement. S6-1. In individual BAB8923, duplications were separated by a 3,084 kb copy number neutral interval region. In this individual, the first TS is mediated by directly-oriented *Alus* with 90% identity. Vertical lines in Jct1 are used to show sequence similarities of *Alus* that created a chimeric element. A three bp insertion was found in Jct2. Coordinates are based on breakpoint junction analysis. Color code for nucleotides: blue for the distal end of join-point, green for the proximal end of join-point, and light brown for insertion.

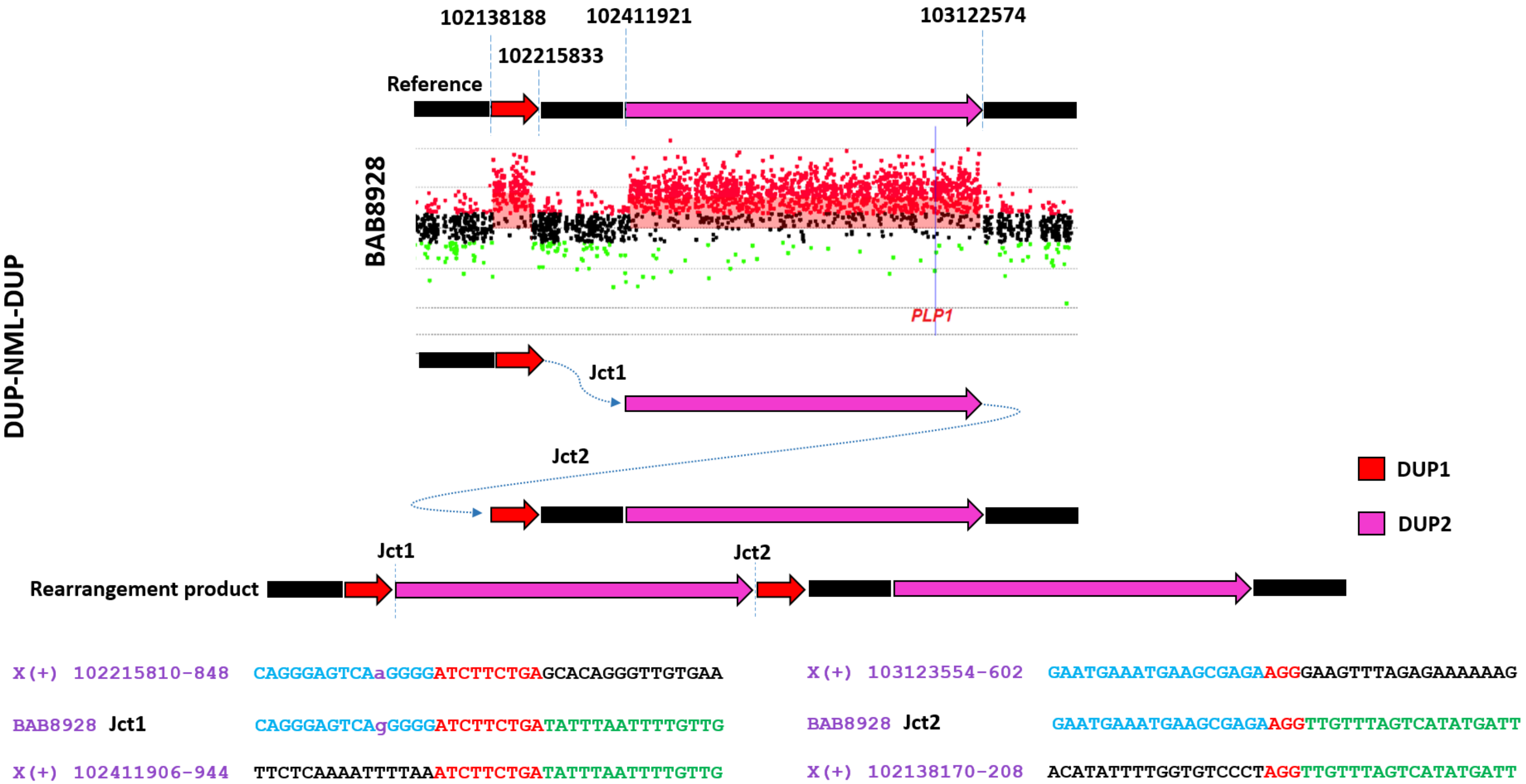

Fig S6-2. In individual BAB8928, junction sequencing revealed that both TSs are microhomology-mediated. Coordinates are based on breakpoint junction analysis. Color code for nucleotides: blue for the distal end of join-point, green for the proximal end of join-point, red for microhomology, and purple for SNV.

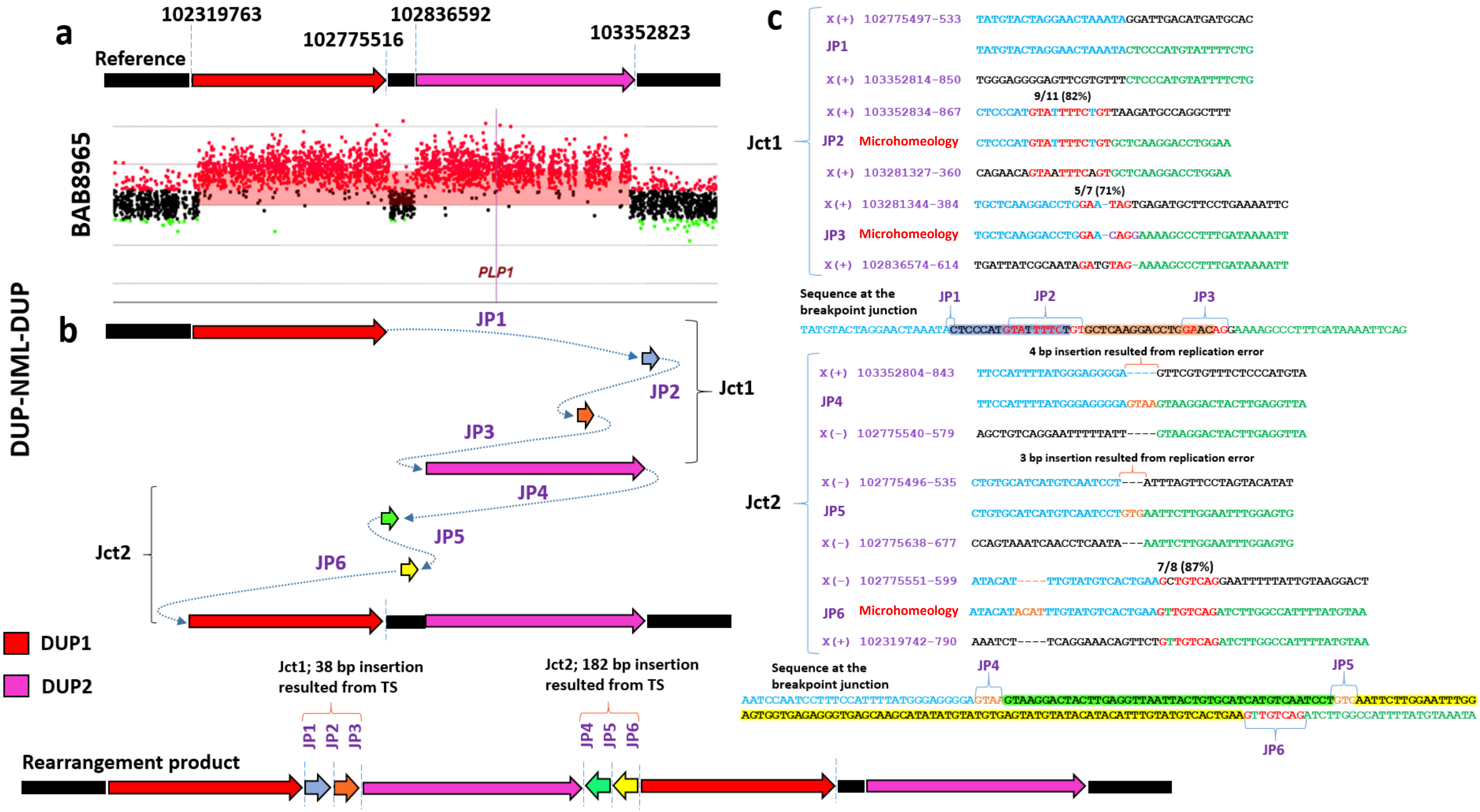

Fig S6-3. In individual BAB8965, the breakpoint junctions suggests at least 6 TS events. Microhomeology was found in three out of six join-points (2, 3, and 6). Two small insertions were observed at Jct2 in this subject. Coordinates are based on breakpoint junction analysis. Color code for nucleotides: blue for the distal end of join-point, green for the proximal end of join-point, red for microhomeology, and light brown, light green and yellow for insertions.

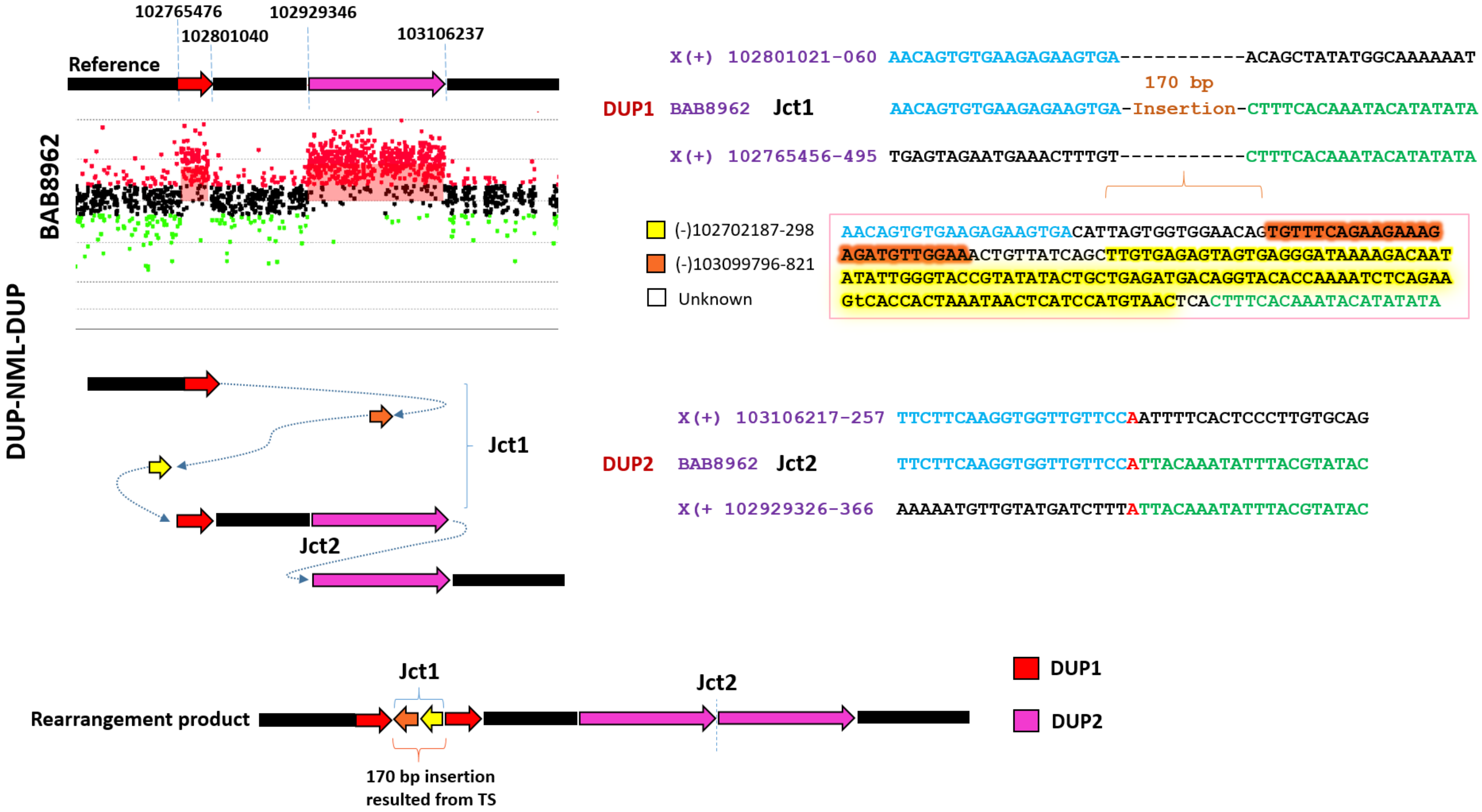

Fig S6-4. Two head-to-tail tandem duplications were detected by aCGH in subject BAB8962. Coordinates are based on breakpoint junction analysis. Color code for nucleotides: blue for the distal end of join-point, green for the proximal end of join-point, red for microhomology, and light brown and yellow for insertions.

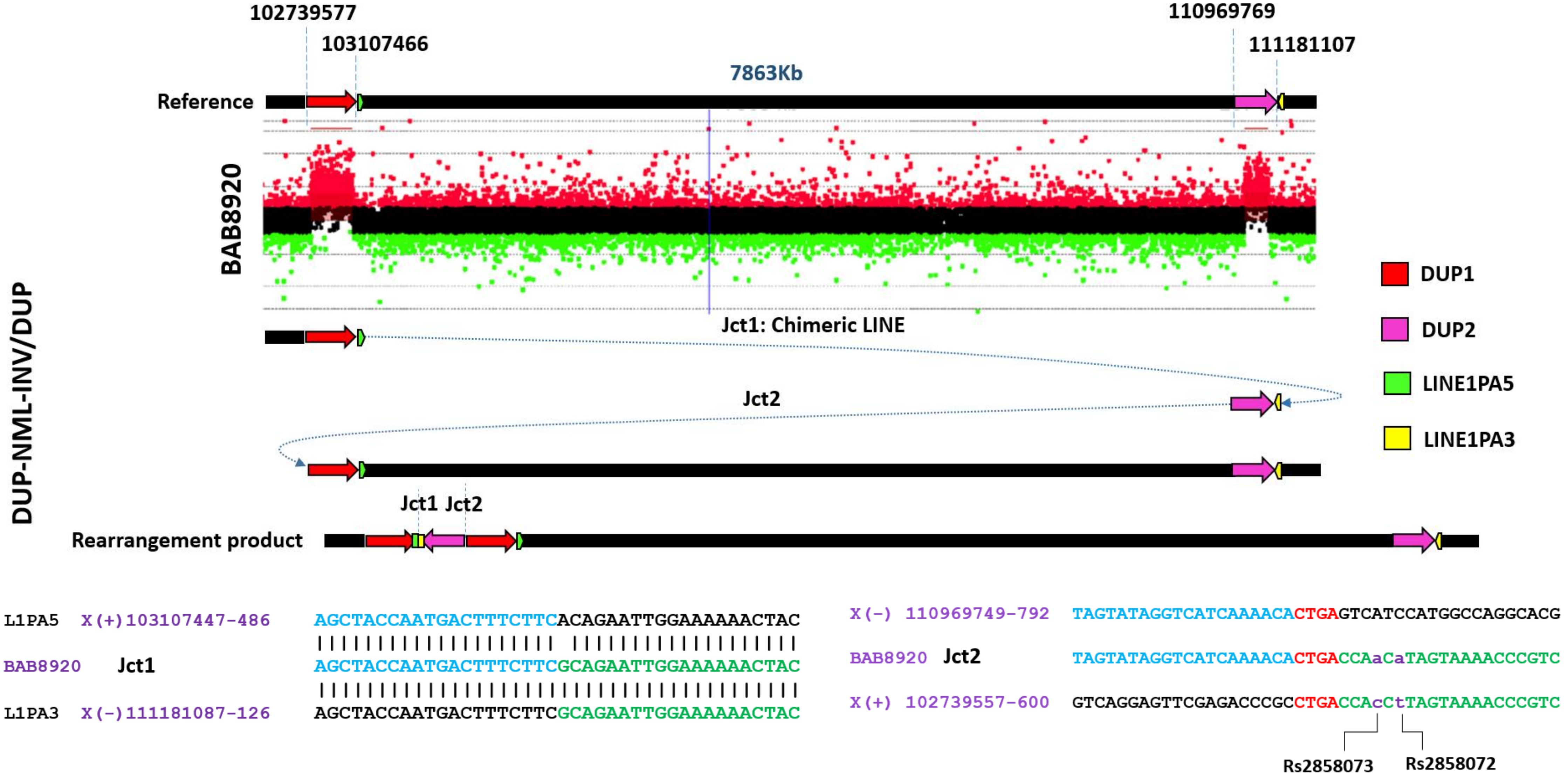

Fig S7. Breakpoint junction analysis indicates that two patients have a DUP–NML–INV/DUP pattern of rearrangement. LINE- or *Alu*-mediated events can be seen in both individuals. The orientation of the LINE and *Alu* interspersed repeats is a determining factor for the overall rearrangement patterns observed in these individuals. **S7-1.** In individual BAB8920, the proximal duplication (including *PLP1*) was separated by a 7,863 kb copy number neutral region from an inverted distal duplication. In this individual breakpoint junction analysis showed that opposite-oriented LINEs, L1PA5 and L1PA3, with 93% identity mediated the first TS, reversing the direction of replication. The second TS is microhomology-mediated restoring the direction of replication. Vertical lines at Jct1 is used to show the sequence similarities of repeats that created chimeric elements. Color code for nucleotides: blue for the proximal side of join-point, green for the distal side of join-point, red for microhomology, and purple for SNPs.

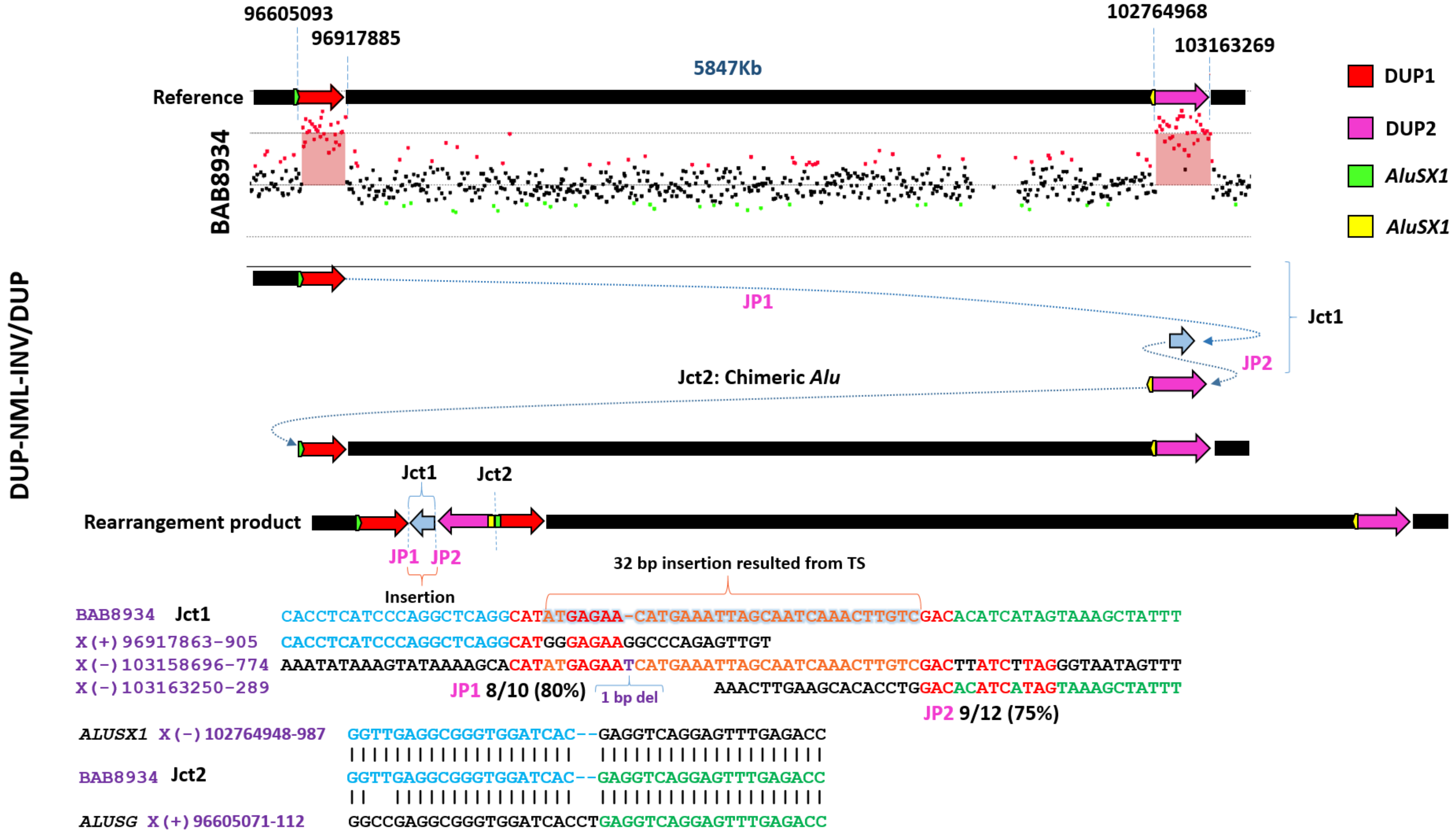

Fig S7-2. In subject BAB8934, we detected a proximal duplication separated by a 5,847 kb copy neutral region from a distal duplication. In this individual two TSs occurred by microhomeologies and a third one by opposite-oriented *Alu-Alu* (both from *AluSX1* family, 89% identity) mediated rearrangement. Vertical lines at Jct2 is used to show the sequence similarities of repeats that created chimeric elements. Color code for nucleotides: blue for the proximal side of join-point, green for the distal side of join-point, red for microhomeology, light brown for insertion, and purple for a single base pair deletion.

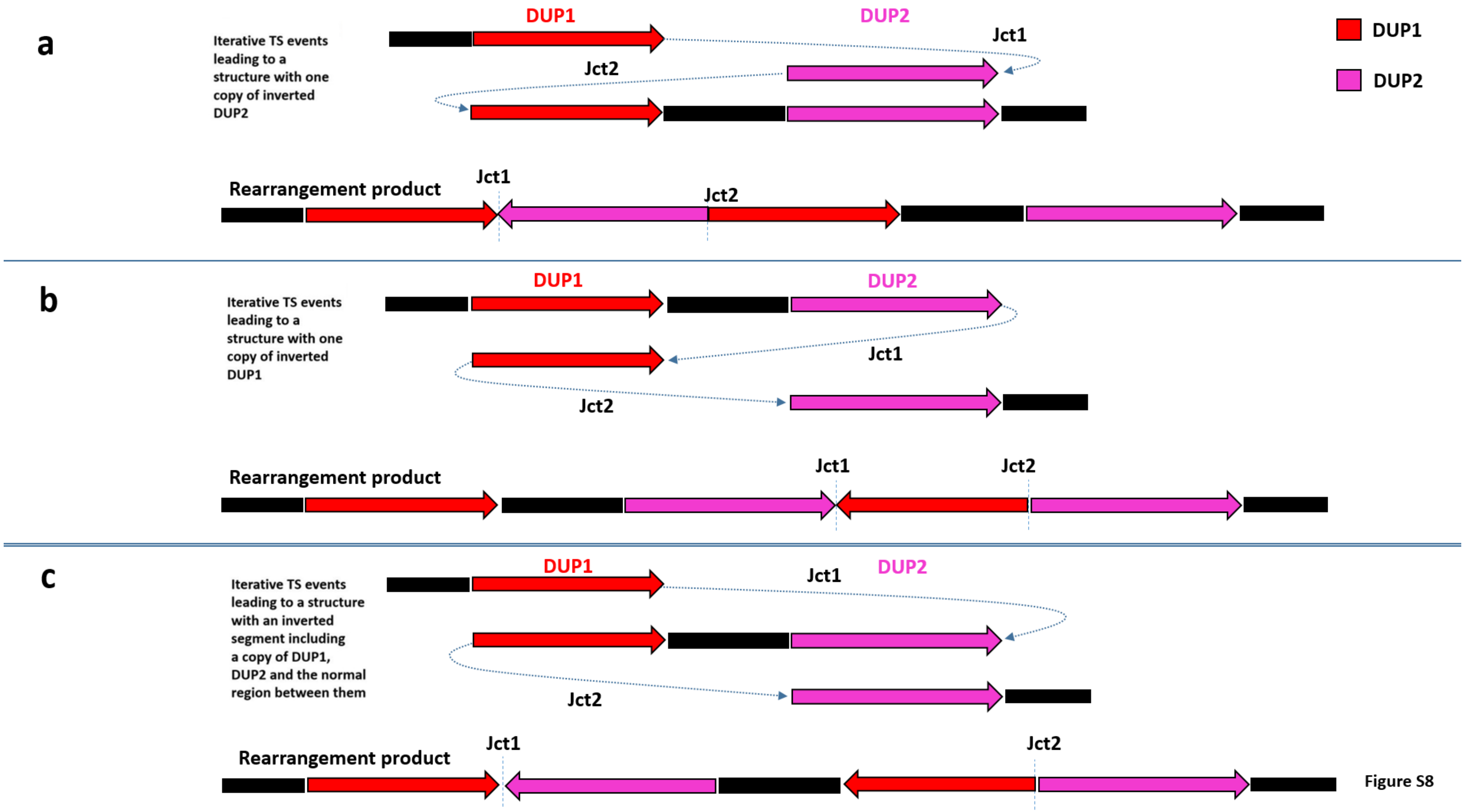

Fig S8. Three possible rearrangements for the generation of DUP–NML–INV/DUP structures satisfy the breakpoint junctions that we obtained on patients BAB8920 and BAB8934. a. As shown in Figure S8, TS from the distal end of DUP1 to distal end of DUP2 in the first TS and from proximal end of DUP2 to proximal end of DUP1 in the second TS would result in inversion of DUP2 and its insertion at the junction between the two copies of DUP1. **b.** A TS from the distal end of DUP2 to distal end of DUP1 in the first TS and proximal end of DUP1 to the proximal end of DUP2 in the second TS would result in inversion of DUP1 and its insertion at the junction between the two copies of DUP2. **c.** A TS from the distal end of DUP1 to the distal end of DUP2 in the first TS and from the proximal end of DUP1 to the proximal end of DUP2 in the second would result in inversion of both duplicated segments and the copy normal region between them. The large arrowheads on the end of genomic segments indicate the orientation of segments with respect to each other on the chromosome and the little arrowheads on blue dotted lines indicate the direction of replication.

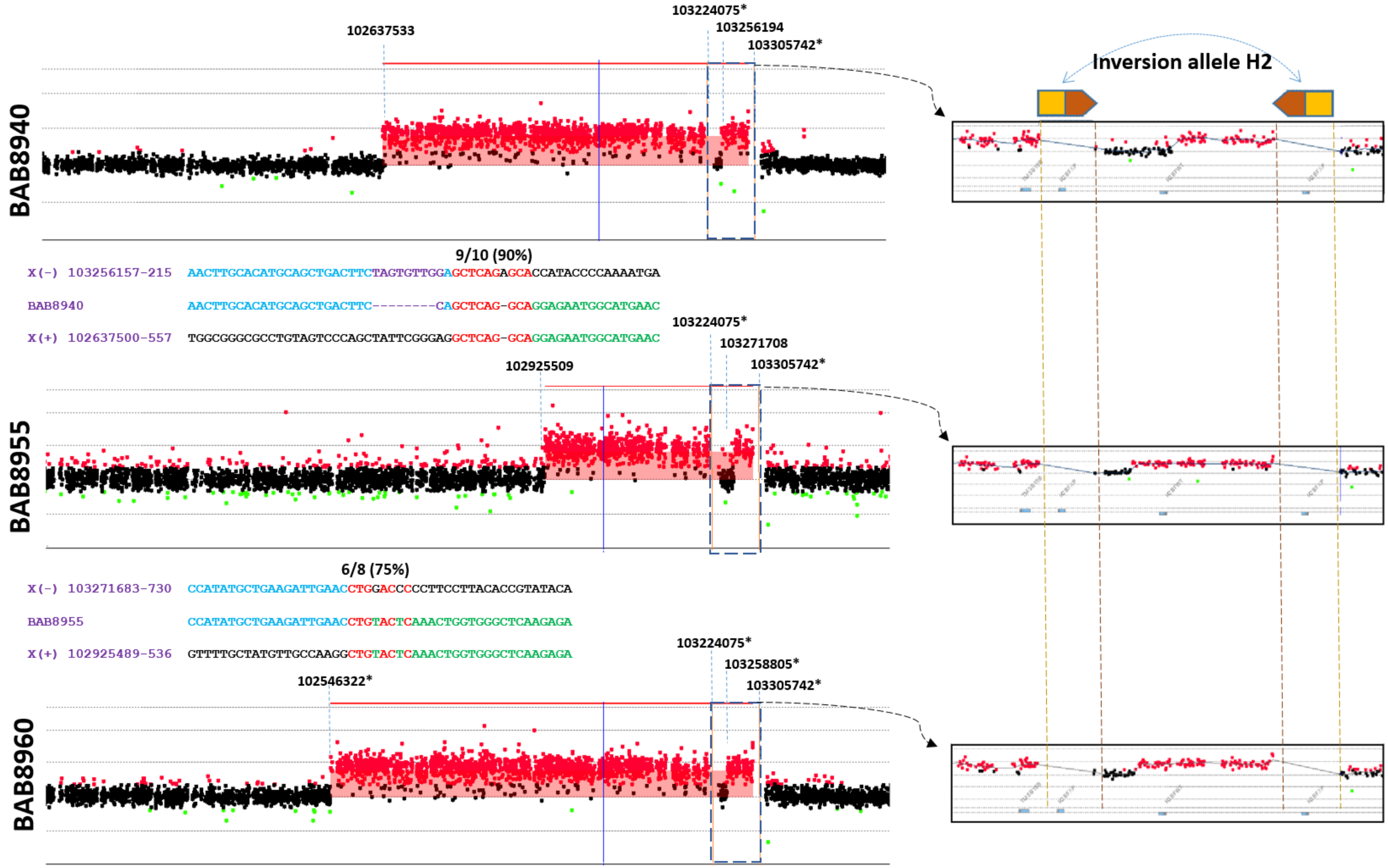

BAB8940
102637533
103224075*
103256194
103305742*
Inversion allele H2
9/10 (90%)
X(-) 103256157-215 AACTTGCACATGCAGCTGACTTCTAGTGTTGGAGCTCAGAGCACCATACCCCAAAATGA
BAB8940 AACTTGCACATGCAGCTGACTTC---------CAGCTCAG-GCAGGAGAATGGCATGAAC
X(+) 102637500-557 TGGCGGGCGCCTGTAGTCCCAGCTATTCGGGAGGCTCAG-GCAGGAGAATGGCATGAAC
BAB8955
102925509
103224075*
103271708
103305742*
6/8 (75%)
X(-) 103271683-730 CCATATGCTGAAGATTGAACCTGGACCCCTTCCTTACACCGTATACA
BAB8955 CCATATGCTGAAGATTGAACCTGTACTCAAACTGGTGGGCTCAAGAGA
X(+) 102925489-536 GTTTTGCTATGTTGCCAAGGCTGTACTCAAACTGGTGGGCTCAAGAGA
BAB8960
102546322*
103224075*
103258805*
103305742*

NA

Fig S9. Three individuals with a DUP-NML-DUP pattern on aCGH (BAB8940, BAB8955, and BAB8960) have the distal duplication and copy neutral region between the two duplications mapping within IRs LCRA1a to LCRA1b. We were able to sequence one breakpoint junction in two of them (BAB8940 and BAB8955) indicating an inversion had occurred. One possibility to explain the inversion is that these patients have a single duplication and their duplication occurred on a known inversion haplotype between LCRA1a and LCRA1b called H2. The blue dashed arrow above the LCRA1a and LCRA1b repeats (orange/yellow arrows) over the expanded portion of the arrays indicates where the inversion occurs. Note that there are no probes on the array in LCRA1a and LCRA1b because they would not be unique. If the duplication in these individuals occurred on the H2 haplotype, the pattern seen on the arrays below would be due to showing the data for H2 individuals on the array with probes positioned for a normal control H1 individual. If the arrays were to be inverted between the LCRs to simulate the H2 inversion haplotype, the duplicated region between the repeats would become part of the proximal duplication on the left and the CNR between the repeats would become part of the CNR distal of the duplication, indicating that these individuals would have a single duplication. Another possibility is that the inversion occurred as the result of a second TS between the inverted IRs LCRA1a and LCRA1b. In BAB8940, we found a 9 bp indel, flanking the breakpoint junction that was likely due to a replicative repair error. Coordinates are based on breakpoint junction analysis where possible. Those based on aCGH are marked by *. Color code for nucleotides: blue for the proximal end of join-point, green for the distal end of join-point, red for microhomeology, and purple for deletion.

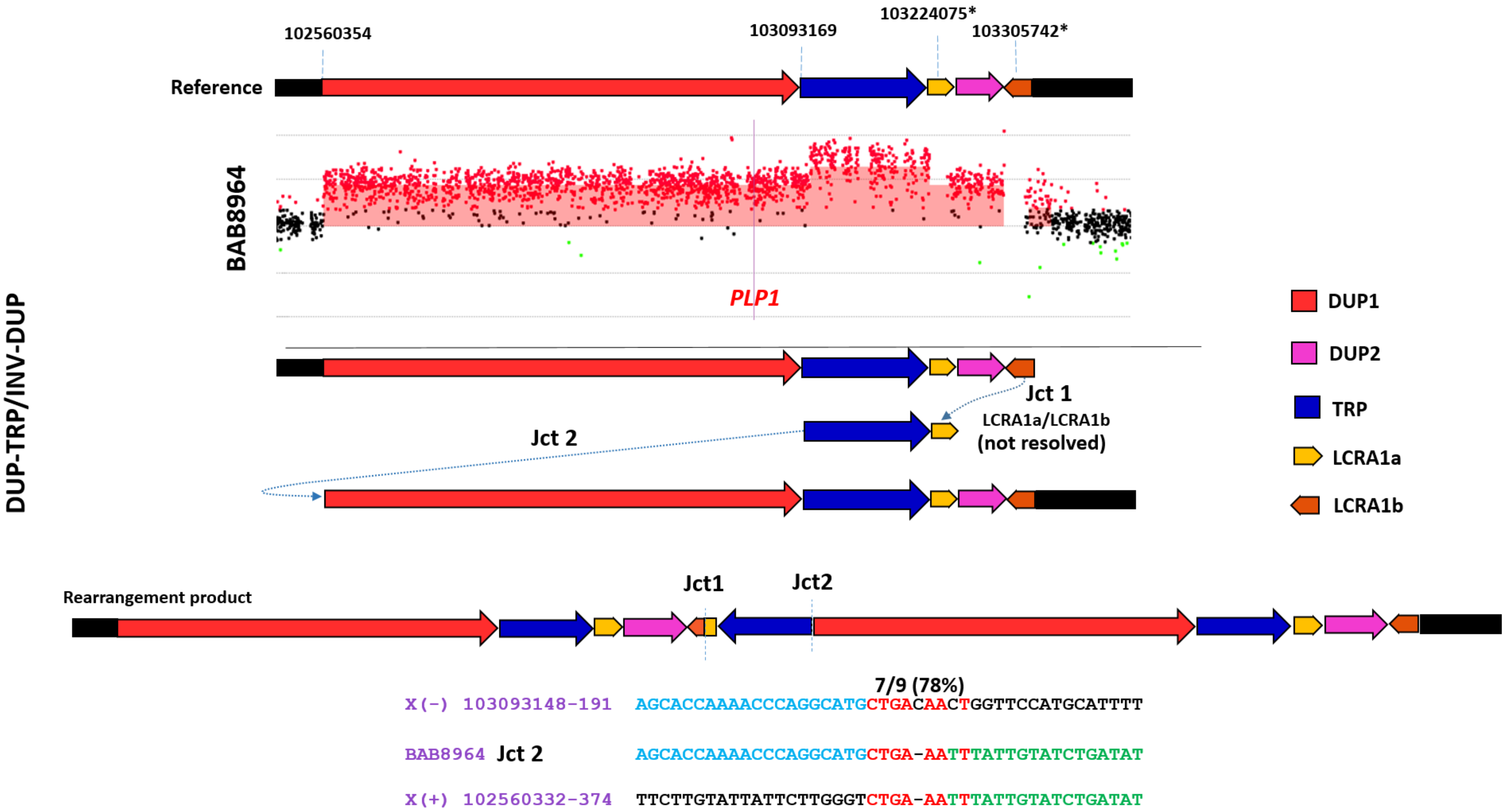

Fig S10. CGRs with DUP-TRP-DUP pattern of rearrangement on aCGH. In two out of three subjects BAB8964 (**S10-1**) and BAB8970 (S10-2), a pattern shift from triplication to duplication was seen to occur at LCRA1a on the array. **S10-1.** The breakpoint junction resolved in BAB8964 indicated that an inversion had occurred, a DUP-TRP/INV-DUP structure, as was the case in the previously published triplication rearrangements at the *PLP1* locus. The genomic rearrangement in BAB8964 probably resulted from two iterative TS events creating the inversion, the first strand TS mediated by LCRA1a and LCRA1b reversing the direction of replication, and the second strand TS mediated by a microhomeology reversing the direction of replication back to the original direction. Color code for nucleotides in BAB8964: blue for the proximal end of triplication, green for the proximal end of duplication, and red for microhomeology. Coordinates are based on breakpoint junction analysis where possible. Those based on aCGH are marked by *.

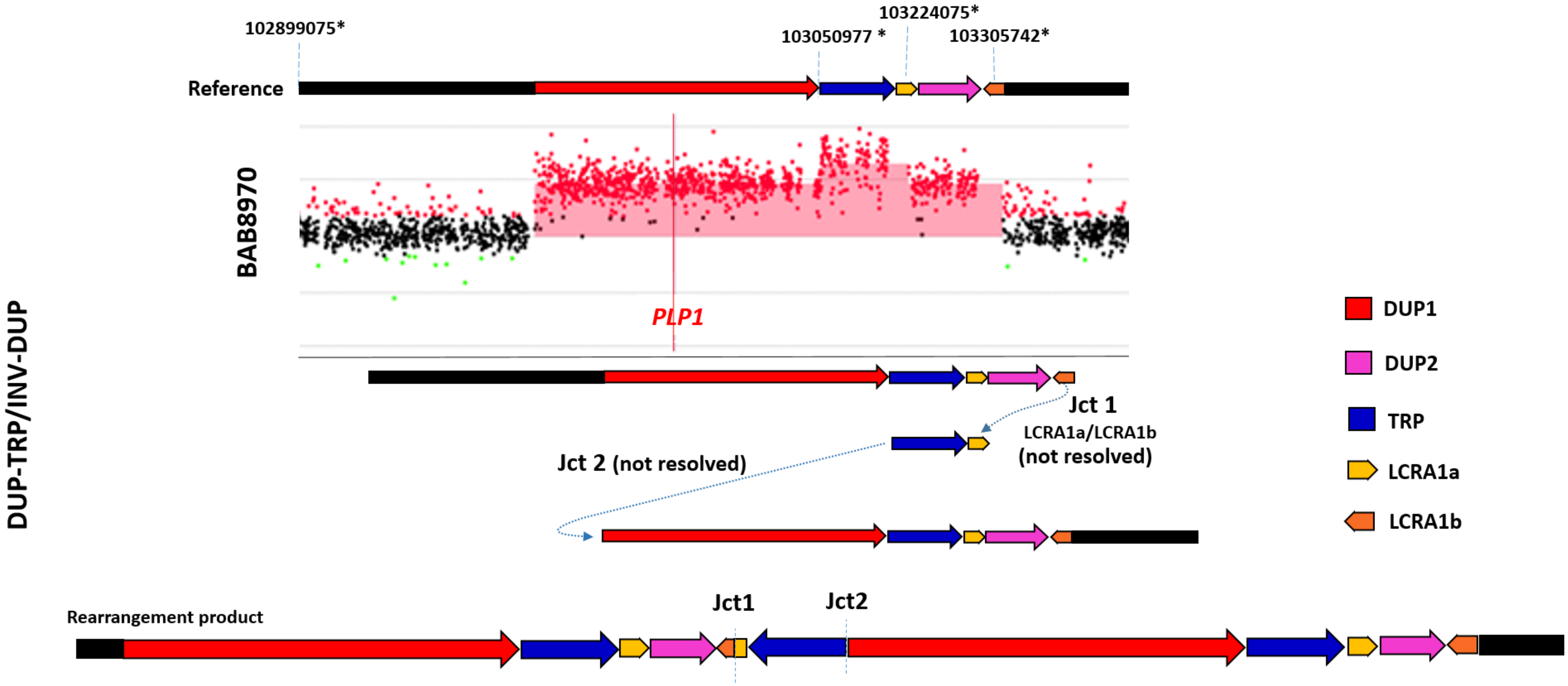

Fig S10-2. We were not able to resolve a junction in BAB8970, but it is likely to be the same DUP-TRP/INV-DUP structure. Coordinates based on aCGH and are marked by *.

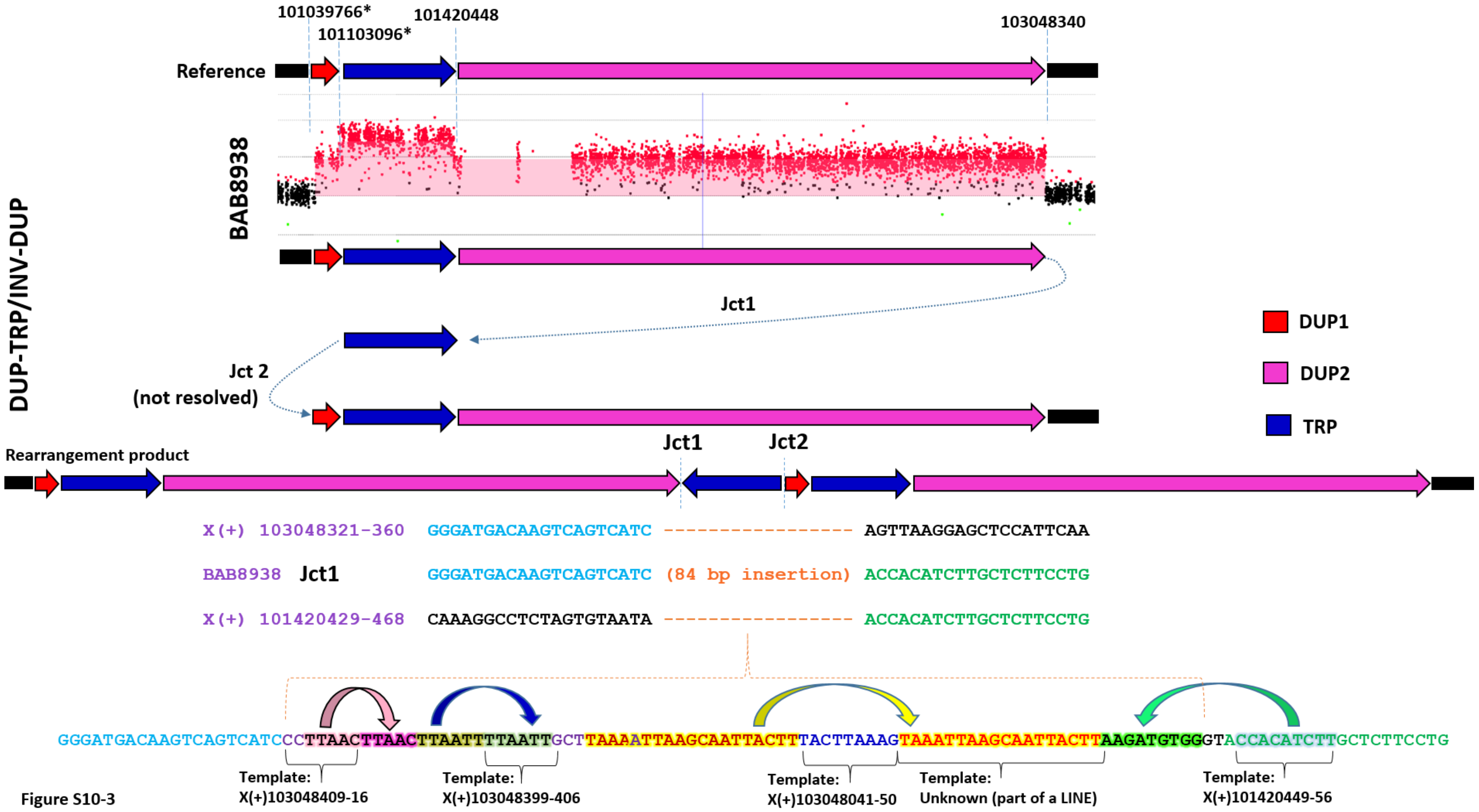

Fig S10-3. The breakpoint junction resolved in BAB8938 indicated that an inversion had occurred, a DUP-TRP/INV-DUP structure, as in BAB8964 and probably BAB8970, but the rearrangement was not mediated by LCRA1a and LCRA1b. There is an 84 bp insertion likely originating from replication of several distinct templates, most of which can be found nearby the junction. The sequence across this breakpoint junction has three direct repeats (indicated by pink, blue, and yellow curved arrows) and an inverted repeat of 10 bases (indicated by curved green arrow). Coordinates are based on breakpoint junction analysis where possible. Those based on aCGH are marked by *. Color code for nucleotides in BAB8938: blue for the distal end of duplication, green for the distal end of triplication, and light brown for insertion.

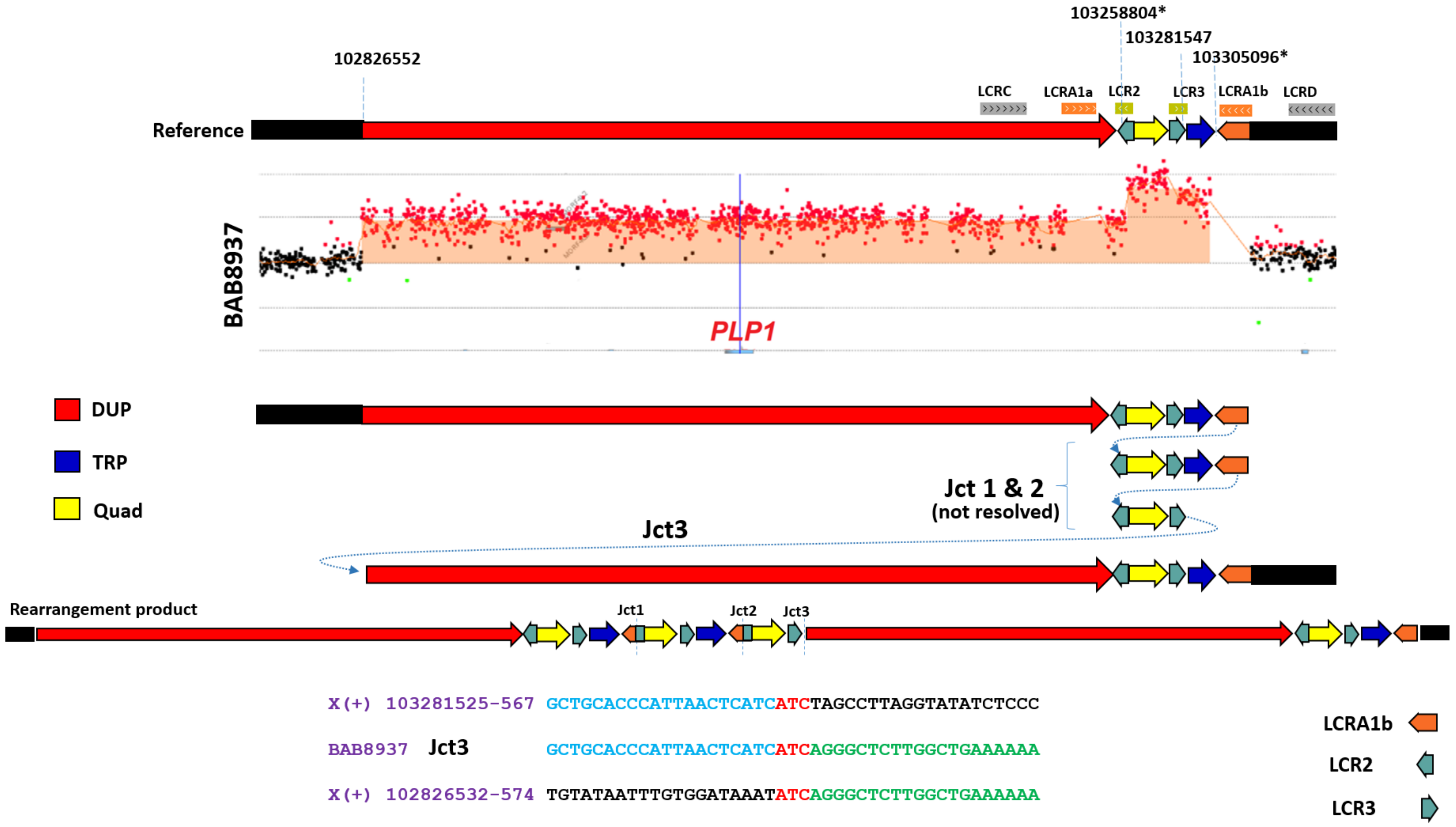

Fig S11. The most complex rearrangement in this study, DUP-TRP-QUAD, was observed in individual BAB8937. In the previously published case with this pattern of rearrangement, breakpoint analysis revealed the presence of three breakpoint junctions in which two out of three junctions (Jct1 & 2) were identical [31]. In this case also three Jcts are expected, and two of them (Jct1 & 2) are likely identical. The copy number changes in the distal part of this rearrangement were located in the homologous LCR2 and LCRA1b sequences, indicating a possible role for NAHR between these elements in the formation of CGRs with higher order amplifications. LCR2 (olive green) has 87% homology with LCRA1b (orange). Coordinates are based on breakpoint junction analysis where possible. Those based on aCGH are marked by *. Color code for nucleotides: blue for the distal end of quadruplicated segment, green for the proximal end of duplication, red for microhomology.

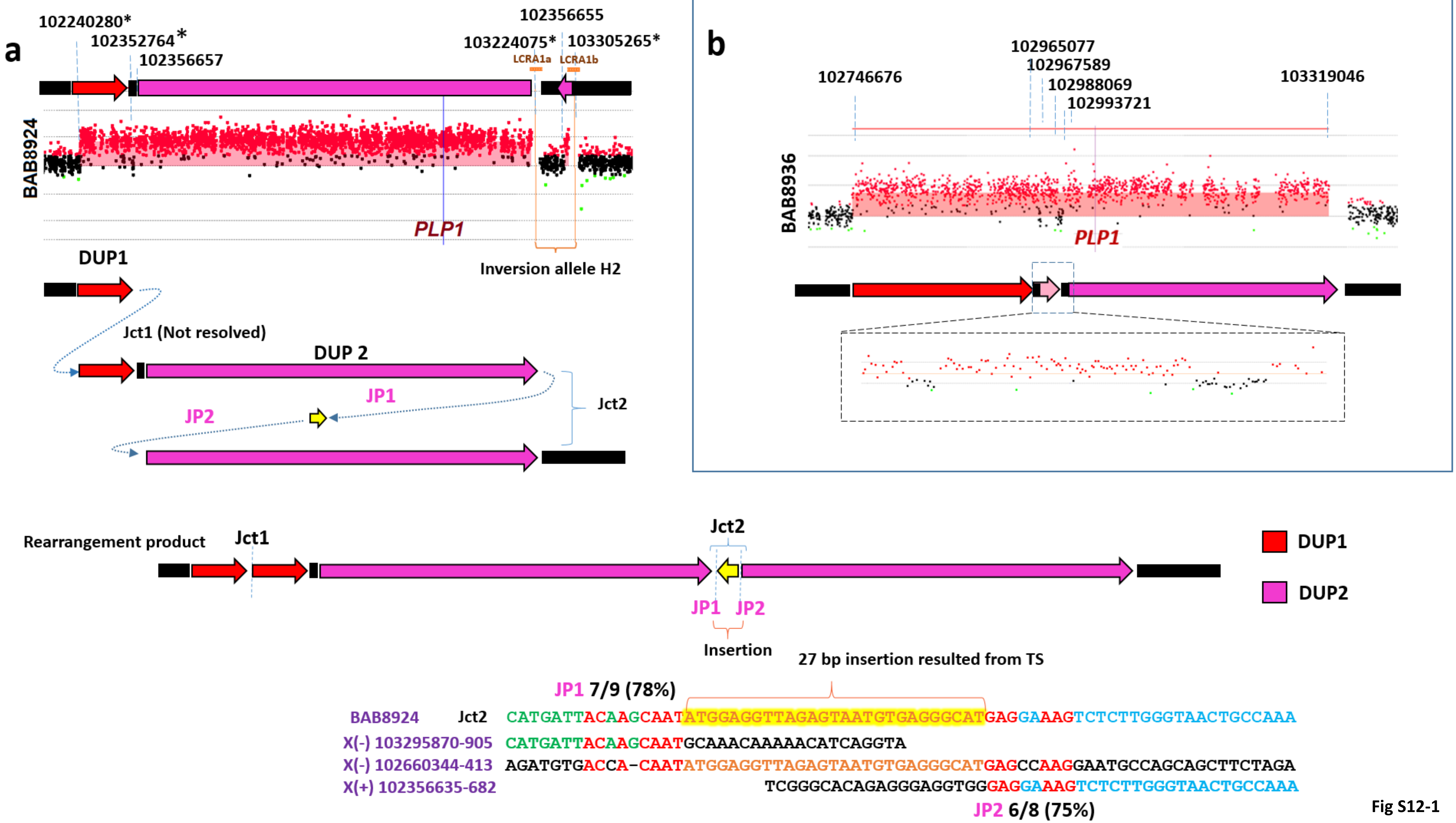
a
102240280*
102352764*
102356657
102356655
103224075*
103305265*
LCRA1a
LCRA1b
BAB8924
PLP1
Inversion allele H2
DUP1
Jct1 (Not resolved)
DUP 2
JP1
JP2
Jct2
b
102965077
102967589
102746676
102988069
103319046
102993721
BAB8936
PLP1
Rearrangement product
Jct1
Jct2
JP1
JP2
Insertion
DUP1
DUP2
27 bp insertion resulted from TS
JP1 7/9 (78%)
BAB8924 Jct2 CATGATTACAAGCAATATGGAGGTTAGAGTAATGTGAGGGCATGAGGAAAGTCTCTTGGGTAACTGCCAAA
X(-) 103295870-905 CATGATTACAAGCAATGCAAACAAAAACATCAGGTA
X(-) 102660344-413 AGATGTGACCA-CAATATGGAGGTTAGAGTAATGTGAGGGCATGAGCCAAGGAATGCCAGCAGCTTCTAGA
X(+) 102356635-682 TCGGGCACAGAGGGAGGTGGGAGGAAAGTCTCTTGGGTAACTGCCAAA
JP2 6/8 (75%)

Fig S12-1

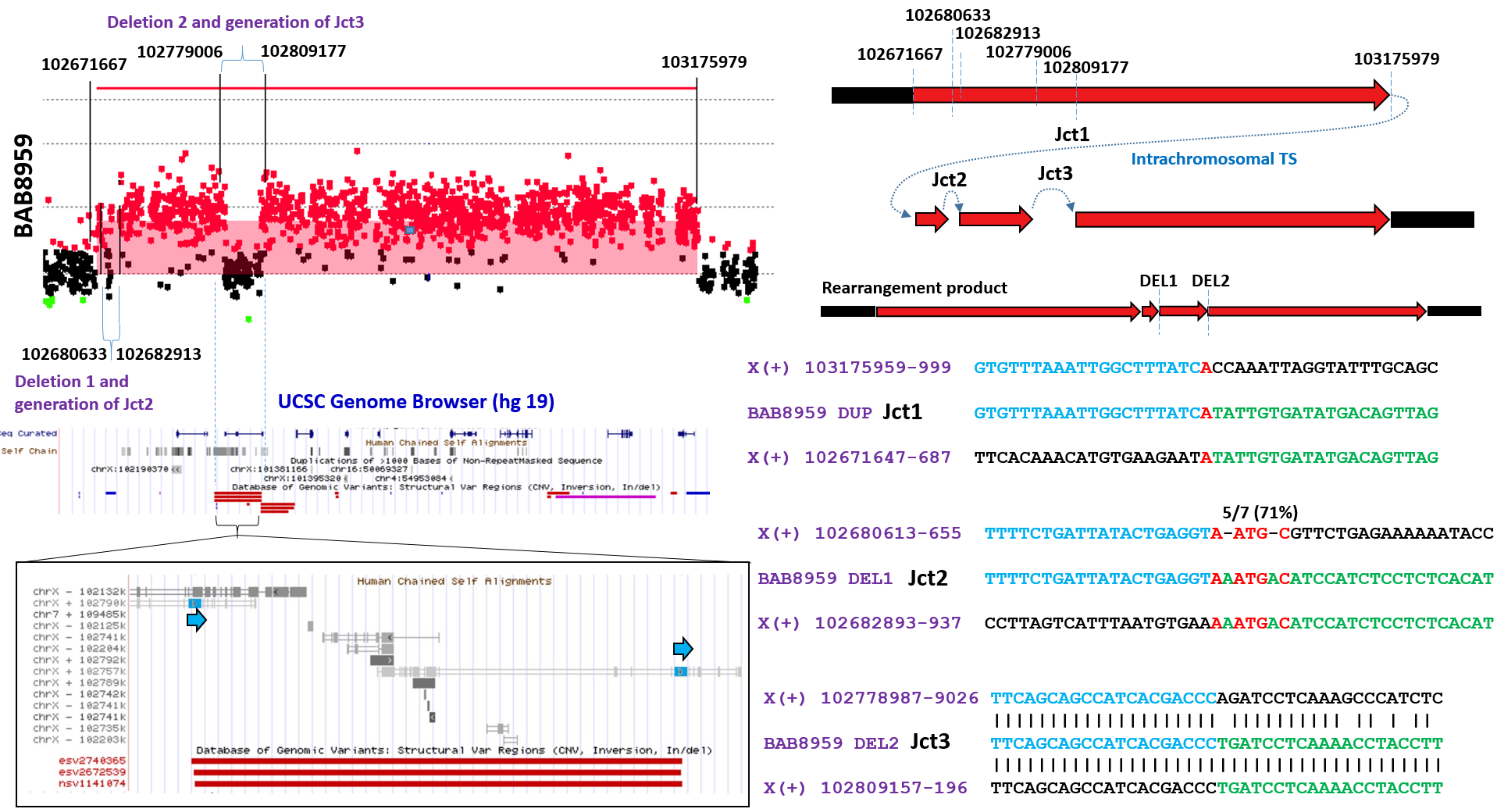

Deletion 2 and generation of Jct3
102671667
102779006
102809177
103175979
BAB8959
102680633
102682913
Deletion 1 and generation of Jct2
UCSC Genome Browser (hg 19)
Human Chained Self Alignments
Duplications of >1000 Bases of Non-RepeatMasked Sequence
Database of Genomic Variants: Structural Var Regions (CNV, Inversion, In/del)
esv2740365
esv2672539
nsv1141074
Jct1
Intrachromosomal TS
Jct2
Jct3
Rearrangement product
DEL1
DEL2
X(+) 103175959-999 GTGTTTAAATTGGCTTTATCACCAAATTAGGTATTTGCAGC
BAB8959 DUP Jct1 GTGTTTAAATTGGCTTTATCATATTGTGATATGACAGTTAG
X(+) 102671647-687 TTCACAAACATGTGAAGAATATATTGTGATATGACAGTTAG
5/7 (71%)
X(+) 102680613-655 TTTTCTGATTATACTGAGGTA-ATG-CGTTCTGAGAAAAAATACC
BAB8959 DEL1 Jct2 TTTTCTGATTATACTGAGGTAAATGACATCCATCTCCTCTCACAT
X(+) 102682893-937 CCTTAGTCATTTAATGTGAAAAATGACATCCATCTCCTCTCACAT
X(+) 102778987-9026 TTCAGCAGCCATCACGACCCAGATCCTCAAAGCCCATCTC
BAB8959 DEL2 Jct3 TTCAGCAGCCATCACGACCCTGATCCTCAAAACCTACCTT
X(+) 102809157-196 TTCAGCAGCCATCACGACCCTGATCCTCAAAACCTACCTT

Fig S12-2

Fig S12. Samples with DUP-NML-DUP-NML-DUP pattern of rearrangement (based on aCGH).

S12-1a. In BAB8924, the breakpoint junction sequence of Jct2 indicates that an inversion has occurred and the distal copy number neutral region seen on aCGH maps within IRs LCRA1a to LCRA1b, so this may have occurred as a result of displaying the H2 haplotype of the individual on H1 haplotype aCGH, as was the case with BAB8940, BAB8955, and BAB8960. This individual may have two separate tandem head to tail duplications on the H2 haplotype, or may have three duplications, with one of the TSs occurring between IRs LCRA1a and LCRA1b, shifting the direction of replication. **S12-1b.** In BAB8936, we were not able to resolve breakpoint junctions and we do not know if two small copy number neutral regions are products of an unrelated cell division event or events.

S12-2. Breakpoint junction sequencing for the subject BAB8959 revealed deletion junctions corresponding to the two copy neutral regions and a duplication breakpoint junction for the region encompassing all three duplications. We hypothesize that the deletions may have been present on one of the ancestral chromosomes, and an interchromosomal TS during oogenesis with a chromosome not harboring the deletions could explain the genomic rearrangement in this individual. In fact, the coordinates of the larger deletion match closely to three polymorphic deletions, esv2740365, esv2672593, and nsv1141074, in the database of genomic variants (DGV). Homologous self-chain blocks (direct repeats shown by blue rectangles) flank these deletions and could have mediated their formation. In order to test our hypothesis, we performed a genome-wide SNP array on this sample but we did not detect any dimorphic SNPs across the CNV and therefore our results did not support this hypothesis. Therefore, the duplication and deletions seen in this individual likely occurred as part of an intrachromosomal event. Vertical lines (Jct3 in BAB8959) are used to show the sequence similarities of the chimeric element with reference sequences. Coordinates are based on breakpoint junction analysis where possible. Those based on aCGH are marked by *. Color code for nucleotides: blue for the proximal side of join-point, green for the distal side of join-point, and red for microhomology/microhomeology.

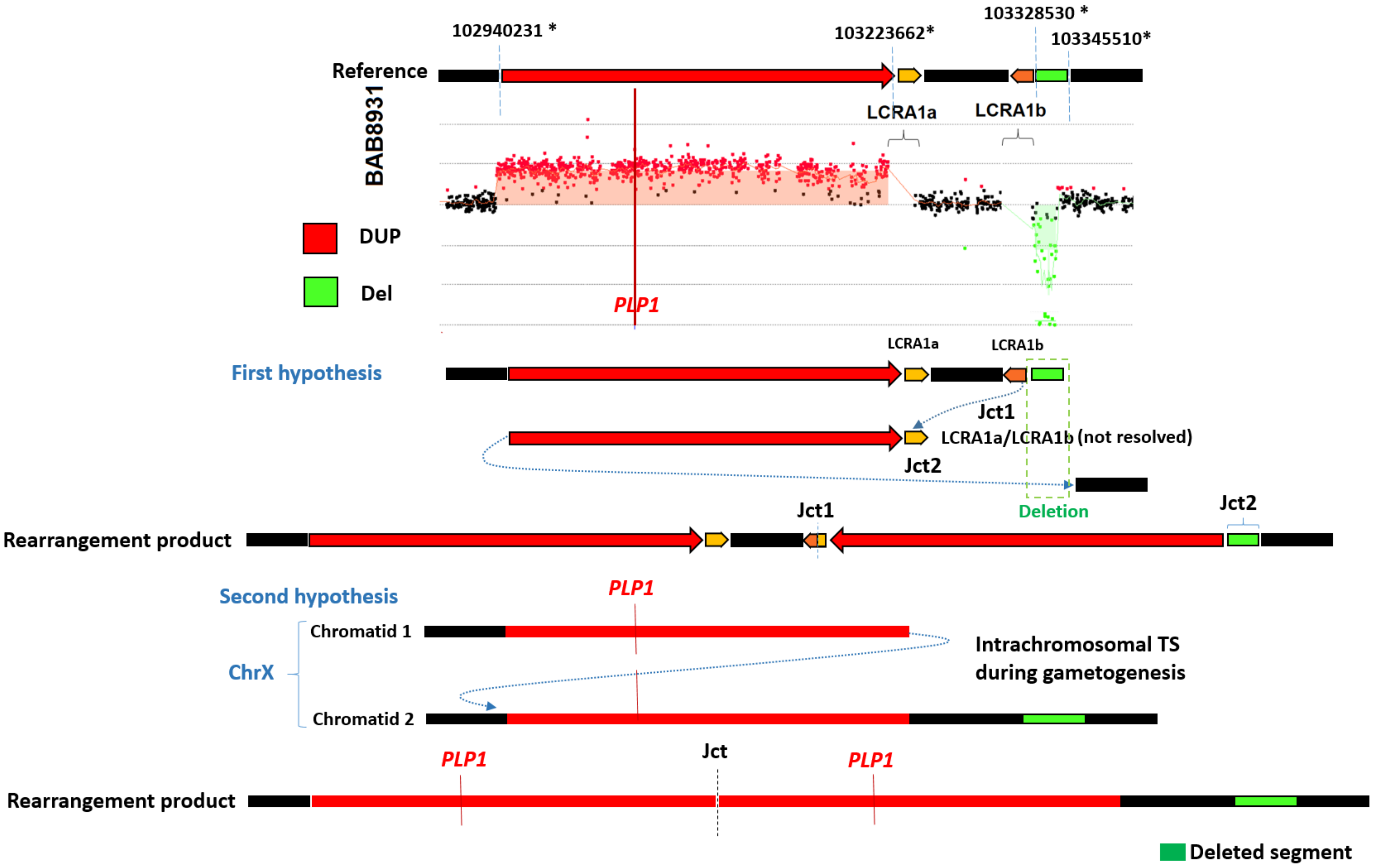

Fig S13. One individual, BAB8931, exhibited DUP-NML-DEL pattern of rearrangement. We detected a ~283 kb duplication (breakpoint junction in LCRA1a), followed by ~106 kb of copy number neutral region and then a ~16kb deletion (breakpoint junction in LCRA1b) in this individual. We present two hypotheses for the formation of this rearrangement. In the first, the rearrangement could be a product of two TS events, the first between IRs LCRA1a and LCRA1b leading to an inverted duplication, and the second from the proximal end of the duplicated region to the distal end of deletion leading to the copy number loss distal of LCRA1b. In the second hypothesis, the rearrangement could be due to an intrachromosomal TS during gametogenesis occurring on a chromosome with an inherited benign deletion. In this case, the de novo duplication would be unrelated to the inherited deletion. No breakpoint junctions were identified in this individual. Coordinates based aCGH are marked by *.

Fig S14. The sequence similarity comparison of reference sequences surrounding join-points. For each of the join-points having a microhomology (2bp or more match; flanked by solid vertical lines) and/or a microhomeology (dotted vertical lines), the 5′ reference sequence of 300 bp in size was plotted as a rectangle on the top while the 3′ was on the bottom. We further group the nucleotide-level join-points resolved in this project into three groups:

S14a. 15 join-points where only a microhomology is present. The heat map shading indicates the sequence similarity level of a 20 bp moving window: orange-high similarity, blue-low similarity, and white-gap.

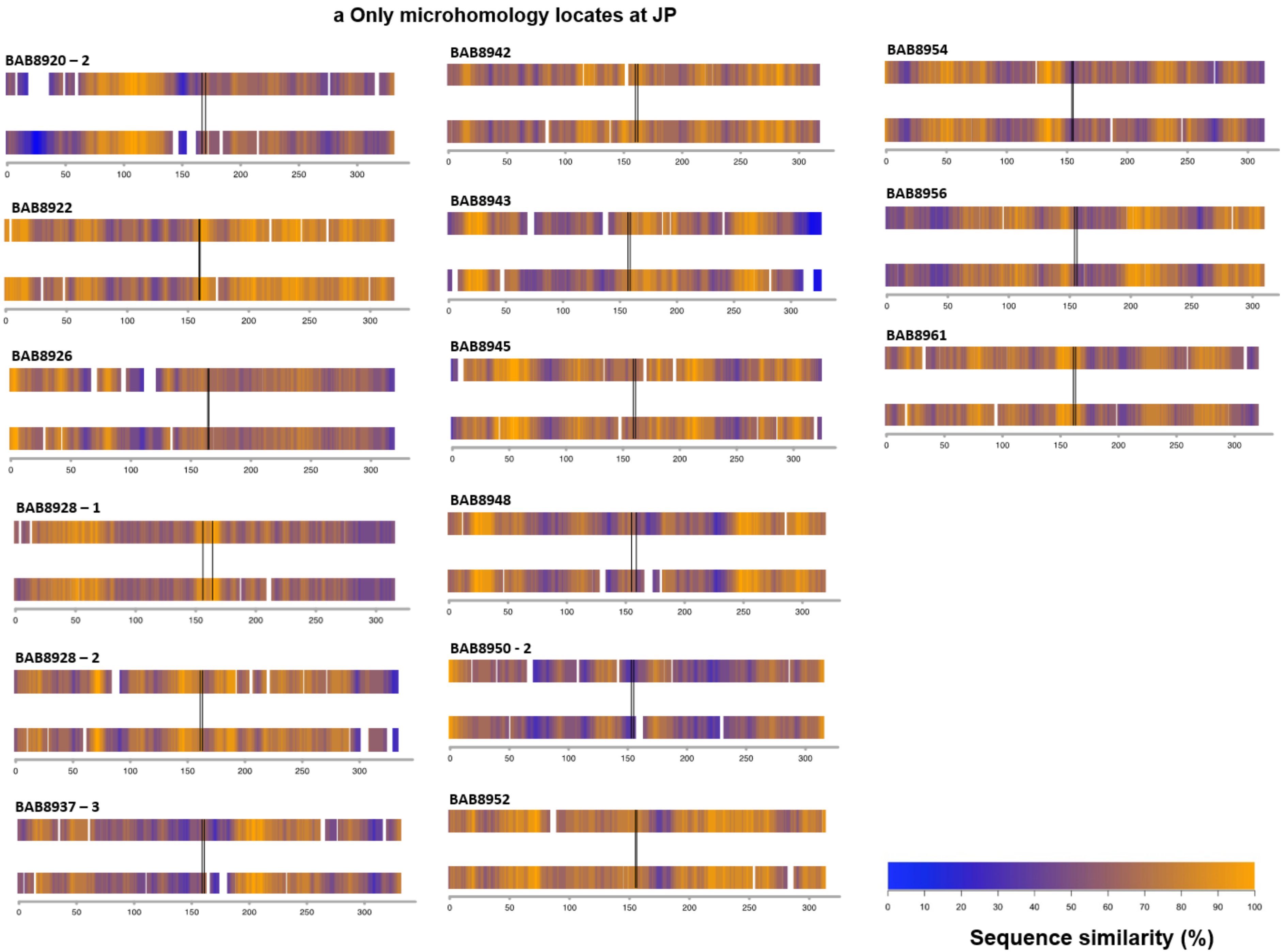

S14b. 16 join-points where both a microhomology and a microhomeology occur. In 12 cases the microhomology is located to one end of the microhomeology, supporting the donor-acceptor hypothesis. The end of the breakpoint with perfect sequence match to the junction (where the microhomology is located) is shown as the priming strand and the end with imperfect matches shown as the target annealing strand. In four cases with microhomology located in the middle of the microhomeology, BAB8944, BAB8947, BAB8953 and BAB8964, we were not able to define the target annealing and priming strands because replication could have occurred in either direction.

S14c. 3 join-points at which only a microhomeology was observed.

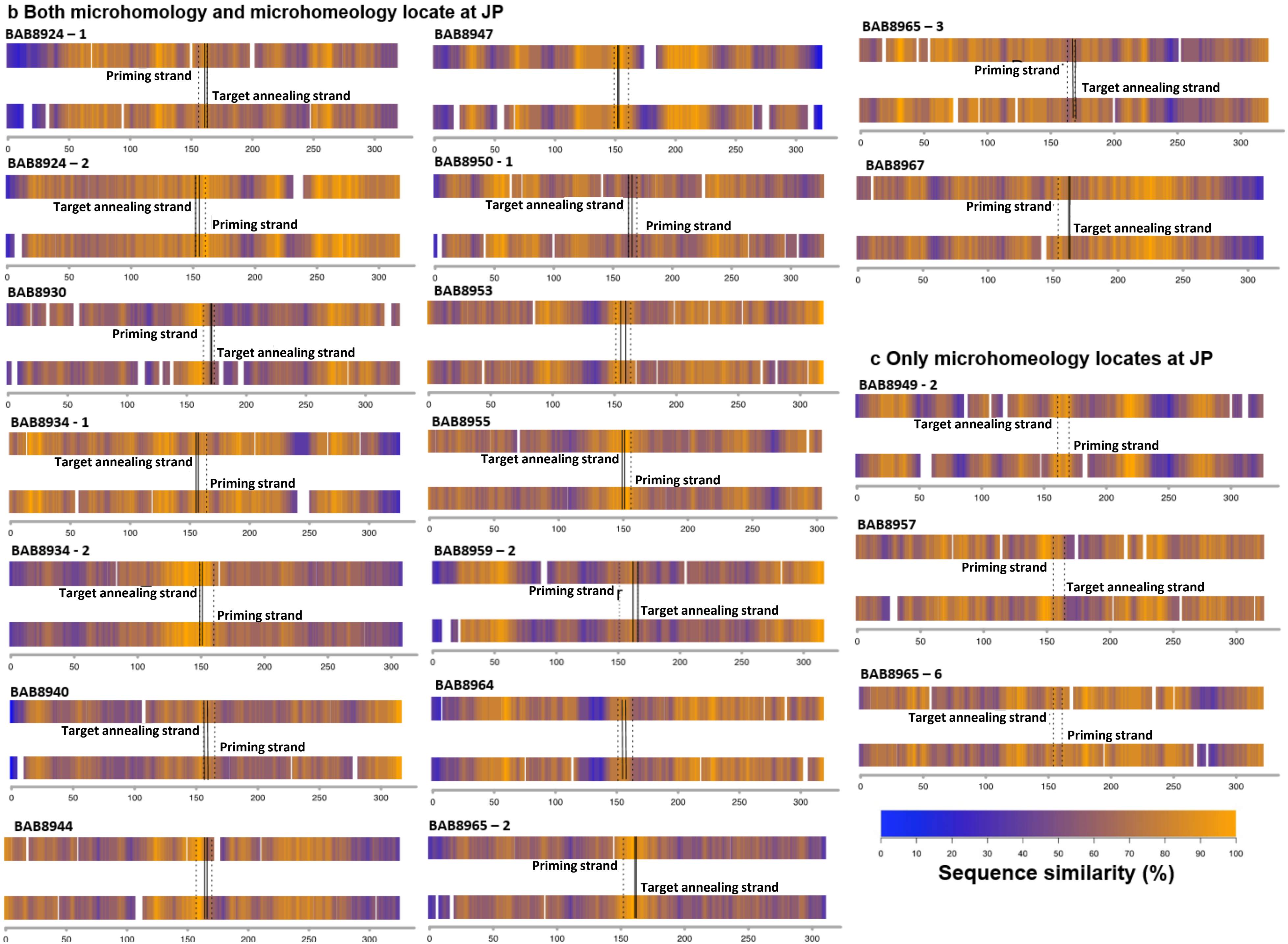

Fig S15. Similarity comparisons of reference sequences surrounding join-points were done after re-analyzing of break-point junction sequences by a retrospective study. For each of the join-points having a microhomology (solid vertical lines) and/or a microhomeology (dotted vertical lines), the 5′ reference sequence of 300 bp in size was plotted as a rectangle on the top while the 3′ was on the bottom.

S15a. 20 join-points where only a microhomology is present (2bp or more match). The heat map shading indicates the sequence similarity level of a 20 bp moving window: orange-high similarity, blue-low similarity, and white-gap.

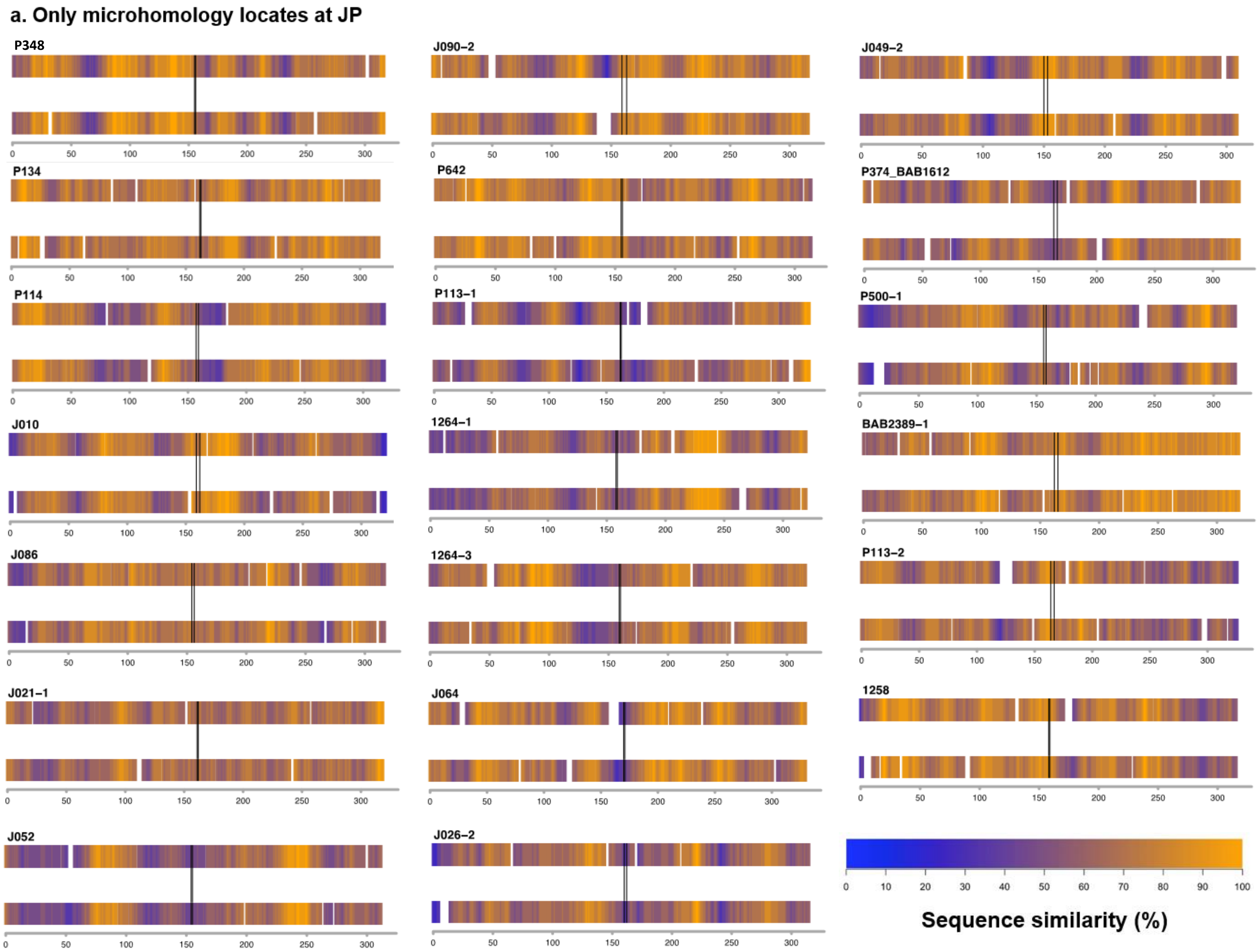

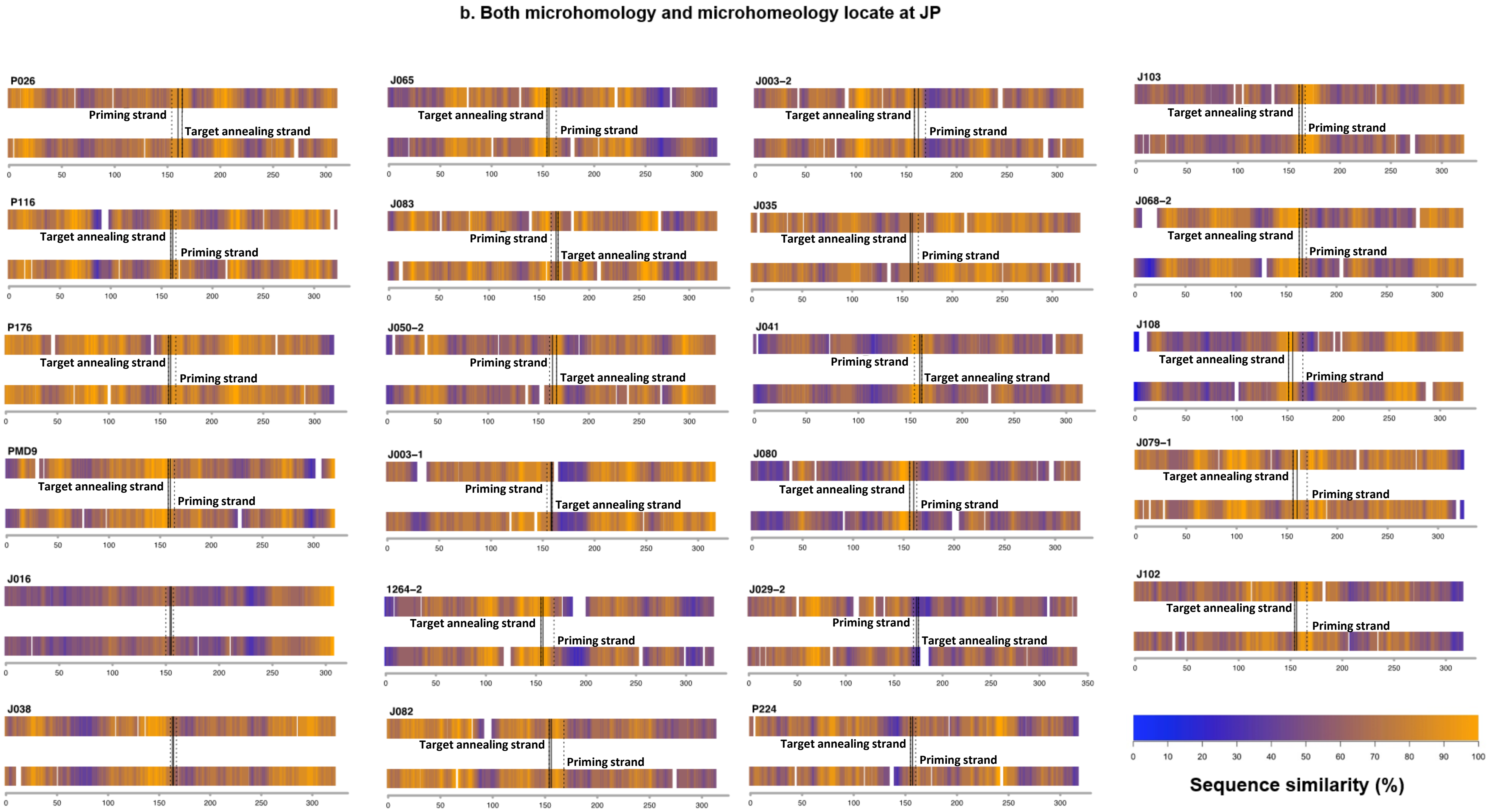
b. Both microhomology and microhomeology locate at JP
P026
Priming strand
Target annealing strand
J065
Target annealing strand
Priming strand
J003–2
Target annealing strand
Priming strand
J103
Target annealing strand
Priming strand
P116
Target annealing strand
Priming strand
J083
Priming strand
Target annealing strand
J035
Target annealing strand
Priming strand
J068–2
Target annealing strand
Priming strand
P176
Target annealing strand
Priming strand
J050–2
Priming strand
Target annealing strand
J041
Priming strand
Target annealing strand
J108
Target annealing strand
Priming strand
PMD9
Target annealing strand
Priming strand
J003–1
Priming strand
Target annealing strand
J080
Target annealing strand
Priming strand
J079–1
Target annealing strand
Priming strand
J016
1264–2
Target annealing strand
Priming strand
J029–2
Priming strand
Target annealing strand
J102
Target annealing strand
Priming strand
J038
J082
Target annealing strand
Priming strand
P224
Target annealing strand
Priming strand
0 10 20 30 40 50 60 70 80 90 100
Sequence similarity (%)

Fig S15-2

S15b. 29 join-points where both a microhomology and a microhomeology occur. In 25 cases the microhomology is located to one end of the microhomeology and defined as the priming strand and the end with imperfect matches is defined as the target annealing strand. In four cases with microhomology located in the middle of the microhomeology, we were not able to define the target annealing and priming strands.

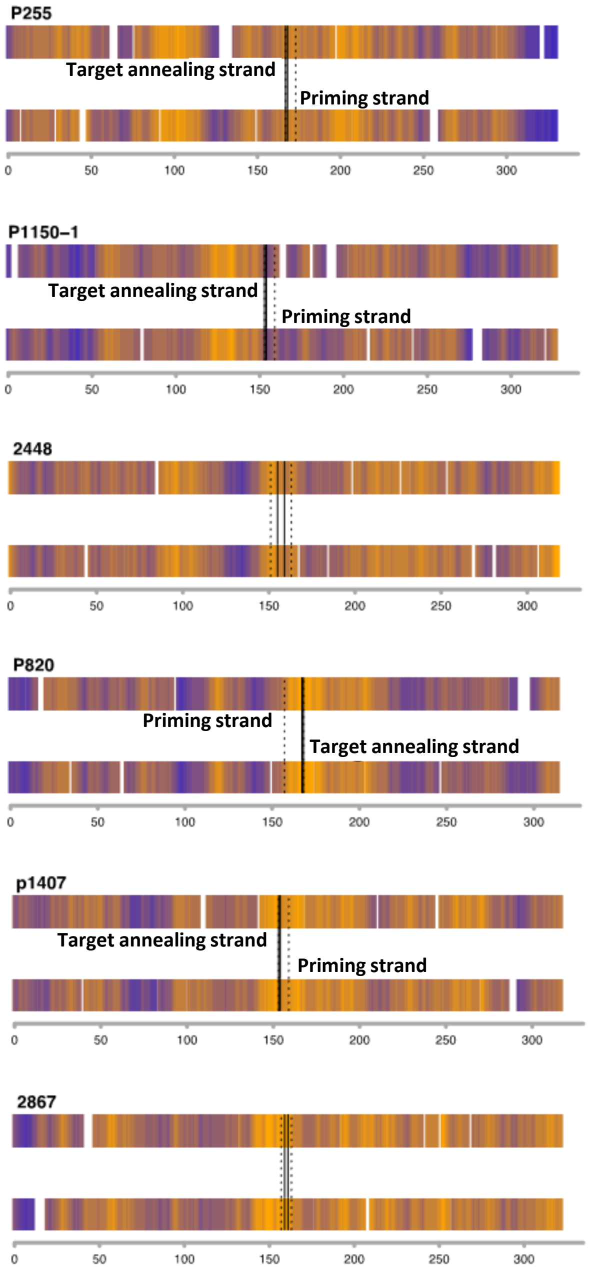

S15c. 3 join-points at which only a microhomeology was observed.

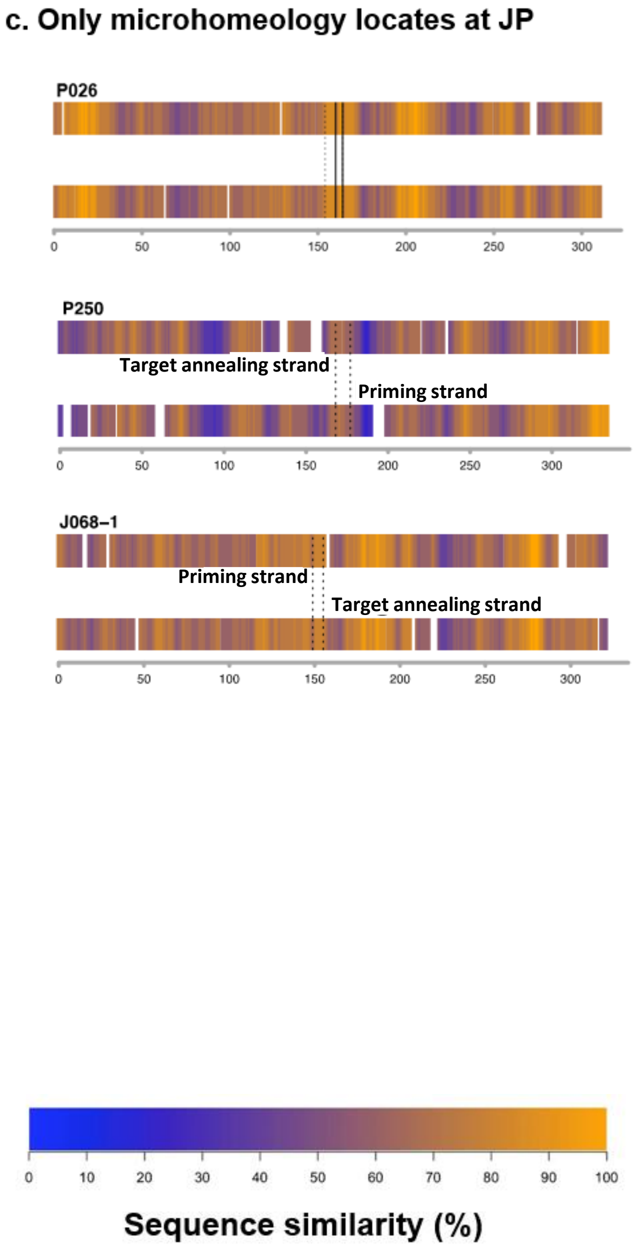

Supplement: Supplementary file 1 — Additional file 1: Figure S1. (S1-1 to S1-6). aCGH and breakpoint junction sequencing results for 30 of the 33 PMD individuals with single duplications at the PLP1 locus. Figure S2. Breakpoint junction sequencing in subject BAB8949 with a single duplication revealed insertions with multiple join-points at the breakpoint junction. Figure S3. Replication errors at the breakpoint junction and/or flanking regions in BAB8929. Figure S4. The aCGH result for BAB8921 showed a 666 Kb single duplication at the PLP1 locus. Figure S5. The distal breakpoint junction points of genomic rearrangements in 28 PMD subjects are grouped within the LCR distal of PLP1. Figure S6. (S6-1 to S6-4). Breakpoint junction analysis indicates that three patients have a directly oriented DUP-NML-DUP pattern of rearrangement. Figure S7. (S7-1 and S7-2). Breakpoint junction analysis indicates that two patients have a DUP–NML–INV/DUP pattern of rearrangement. Figure S8. Three possible rearrangements for the generation of DUP–NML–INV/DUP structures satisfy the breakpoint junctions that we obtained on patients BAB8920 and BAB8934. Figure S9. Three individuals with a DUP-NML-DUP pattern on aCGH (BAB8940, BAB8955, and BAB8960) have the distal duplication and copy neutral region between the two duplications mapping within IRs LCRA1a to LCRA1b. Figure S10. (S10-1 to S10-3). CGRs with DUP-TRP-DUP pattern of rearrangement on aCGH. Figure S11. The most complex rearrangement in this study, DUP-TRP-QUAD, was observed in individual BAB8937. Figure S12. (S12-1 and S12-2). Samples with DUP-NML-DUP-NML-DUP pattern of rearrangement (based on aCGH). Figure S13. One individual, BAB8931, exhibited DUP-NML-DEL pattern of rearrangement. Figure S14. The sequence similarity comparison of reference sequences surrounding join-points. Figure S15. Similarity comparisons of reference sequences surrounding join-points were done after re-analyzing of break-point junction sequences by a retrospective study. [file 13073_2019_676_MOESM1_ESM.pdf]
