# Supplementary material for: Distinct patterns of complex rearrangements and a mutational signature of microhomeology are frequently observed in PLP1 copy number gain structural variants
Source: Genome Med. 2019 Dec 9;11:80. doi: 10.1186/s13073-019-0676-0 (PMC6902434; doi:10.1186/s13073-019-0676-0)
Supplement: Supplementary file 2 — Additional file 2: Table S1. Samples with single duplications at the PLP1 locus. Table S2. A summary of genomic rearrangements, coordinates and breakpoint junctions in the cohort of 50 PMD patients. Table S3. Original data from 7 studies on genomic rearrangements at the PLP1 locus. Table S4. Microhomeologous sequences at the join-points found in this study. Table S5. Other features at the join-points found in this study. Table S6. Microhomeologous sequences at the join-points found by re-analyzing breakpoint sequences from previous studies. [file 13073_2019_676_MOESM2_ESM.pdf]

Table S1. Samples with single duplications at the *PLP1* locus.

Single duplication		
No	Individual	Size of duplication (kb)
1	BAB8921	666*
2	BAB8922	475
3	BAB8925	429*
4	BAB8926	534
5	BAB8929	513
6	BAB8930	555
7	BAB8932	221*
8	BAB8933	551
9	BAB8935	340
10	BAB8939	705*
11	BAB8942	389
12	BAB8943	516
13	BAB8944	562
14	BAB8945	440
15	BAB8946	362
16	BAB8947	367
17	BAB8948	423
18	BAB8949	998
19	BAB8950	429
20	BAB8951	488
21	BAB8952	427
22	BAB8953	425
23	BAB8954	4,484
24	BAB8956	758
25	BAB8957	949
26	BAB8958	257*
27	BAB8961	841
28	BAB8963	611
29	BAB8966	397
30	BAB8967	683
31	BAB8968	122*
32	BAB8969	404
33	BAB8971	366*

*Estimated size based on aCGH

Table S2. A summary of genomic rearrangements, coordinates and breakpoint junctions in the cohort of 50 PMD patients

Individual	Array pattern	Segment size (kb)	Coordinates based on sequences junction, if not achieve they are estimated based aCGH and marked by *	Rearrangement pattern based on Jct sequencing	Coordinates Based on the sequenced Jct (if achieved)	Features of JPs (nucleotides with microhomology /microhomeology are shown as upper case letters and two surrounding Nts by lower case). Gaps in microhomeologies are shown by dash	Inserted sequence () Deleted sequence <> Point mutation [] at join-points	Chimeric element	Junction in repetitive elements/LCRs
8920	DUP1	368	102739577<br>103107466	DUP<br>NML<br>INV/DUP	(+)103107466<br>(-)111181107	74bp Nt identity at Jct	No	Yes (93% identity)	L1PA5/L1PA3
	DUP2	211	110969769<br>111181107		(-)102739577<br>(+)110969769	caCTGAcc	No	No	*Alu*Sq2/-
8921	DUP	666	*102476739<br>103143213	DUP; Insertional translocation	Y(+)23730469<br>X(+)103143213	No	ca(T)gt	No	-/ LCR Y
8922	DUP	475	102713559<br>103188774	Tandem DUP	(+)102713559<br>(+)103188774	gaTCtt	No	No	L1PA4/LCRC
8923	DUP1	642	98855903<br>99498194	DUP<br>NML<br>DUP	(+)98855903<br>(+)103366577	No	at(GAA)tc	No	L1MC4/(TA)n
	DUP2	785	102581871<br>103366577		(+)99498194<br>(+)102581871	cgACAga	No	Yes (90% identity)	*Alu*Y/*Alu*Y
8924	DUP1	112	*102240280<br>*102352764	DUP<br>NML<br>DUP<br>NML<br>DUP<br>or<br>DUP<br>NML<br>DUP<br>on H2 background	NA	NA	NA	NA	NA
	DUP2	866	102356657<br>*103224075		JP1<br>(-)103295890<br>(-)102660402<br><br>JP2<br>(-)102660369<br>(+)102356657	ttACaAgCAATat<br><br>atGAGgaAGtc	at(ATGGAGGTTAGAGTAATGTGAGGGCAT)ga<br><br>The insertion is the result of two microhomeology-mediated TS (JP1 & JP2)	No	LTR (ERV1)/LTR (ERVL-MaLR)/Chain (ID: 1065)
	DUP3	9	102356655<br>*103305265						
8925	DUP	429	*102716349<br>*103145619	Tandem DUP	NA	NA	NA	NA	NA
8926	DUP	534	102793976<br>103322069	Tandem DUP	(+)102793974<br>(+)103322069	ccGAgc	No	No	Chain (ID: 84669)/LCRA1b
8928	DUP	78	102138188<br>102215833	DUP<br>NML<br>DUP	(+)102215833<br>(+)102411921	ggATCTTCTGAta	No	No	-/L1PA15
	DUP	712	102411921<br>103123574		(+)102138188<br>(+)103123574	gaAGGtt	No	No	Chain (ID: 17282) /L1PA3
8929	DUP	513	102651193<br>103164455	Tandem DUP	(+)102651193<br>(+)103164455	No	ctG(gcatggt)ag<br>Rep errors in Jct flanking regions:<br>(-)102651263-74<br>ag<AAAAA>ac<br>(-)102651247-54<br>aa<A>gg<br>(-)102651206-19<br>ga<CTACAAA>tg	No	Chain (ID:95091)/ Chain (ID: 231647)

**8930**	**DUP**	**555**	**102712023<br>103267065**	**Tandem DUP**	**(+)102712024<br>(+)103267065**	**ggTTTCTgTTgAtt**	**No**	**No**	**L1PA4/Chain (ID: 1065)**
**8931**	**DUP**	**283**	***102940231<br>*103223662**	**DUP<br>NML<br>DEL**	**NA**	**NA**	**NA**	**NA**	**Probable LCRA1a/LCRA1b chimera**
	**DEL**	**17**	***103328530<br>*103345510**		**NA**	**NA**	**NA**	**NA**	**NA**
**8932**	**DUP**	**221**	***102842768<br>*103064114**	**NA**	**NA**	**NA**	**NA**	**NA**	**NA**
**8933**	**DUP**	**551**	**102681876<br>103315029**	**Tandem DUP**	**(+)102681876<br>(+)103315029**	**No**	**gg(GATCCCAA)tg**	**No**	**LTR37A/LCRA1b**
**8934**	**DUP1**	**313**	**96605093<br>96917885**	**DUP<br>NML<br>INV/DUP**	**JP1<br>(+)96917885<br>(-)103158716<br><br>JP2<br>(-)103158754<br>(-)103163269**	**ggCATatGAGAAca<br><br>tcGACacATCaTAGta**	**at(ATGAGAATCATGAAATTAGCAATCAAACTTGTC)ga<br><br>The insertion is the result of microhomeology-mediated TSs at JP1 & JP2**	**No<br><br>No**	**-/ NA<br><br>NA/ L1Meg**
	**DUP2**	**398**	**102764968<br>103163269**		**(-)96605093<br>(+)102764968**	**56bp Nt identity at Jct**	**No**	**Yes (89% identity)**	***Alu*Sx1/*Alu*Sg**
**8935**	**DUP**	**340**	**102708892<br>103048730**	**Tandem DUP**	**(+)102708892<br>(+)103048730**	**ccTgc**	**ccT<TTTT>gc**	**No**	**L1MC4/-**
**8936**	**DUP1**	**218**	***102746676<br>*102965077**	**NA**	**NA**	**NA**	**NA**	**NA**	**NA**
	**DUP2**	**20**	***102967589<br>*102988069**						
	**DUP3**	**325**	***102993721<br>*103319046**						
**8937**	**DUP**	**341**	**102826552<br>*103258804**	**DUP<br>QUAD<br>TRP**	**(+)102826552<br>(+)103281547**	**tcATCag**	**No**	**No**	**No**
	**QUAD**	**22**	***103258804<br>103281547**		**NA**	**NA**	**NA**	**NA**	**Presumed LCR2/LCRA1b chimera**
	**TRP**	**17**	**103281547<br>*103305096**		**NA**	**NA**	**NA**	**NA**	**Presumed LCR2/LCRA1b chimera**
**8938**	**DUP1**	**63**	***101039766<br>*101103096**	**DUP<br>TRP/INV<br>DUP**	**(-)103048340<br>(+)101420448**	**No**	**84bp insertion from different templates**	**No**	**LTR (HERV16-int)/-**
	**TRP**	**317**	***101103096<br>101420448**		**NA**	**NA**	**NA**	**NA**	**NA**
	**DUP2**	**1,628**	**101420448<br>103048340**						
**8939**	**DUP**	**705**	***102570985<br>*103275726**	**NA**	**NA**	**NA**	**NA**	**NA**	**NA**

**8940**	**DUP1**<br>**&**<br>**DUP2**	**602**<br><br>**70**	**102637533**<br>***103224075**<br><br>**103256194**<br>***103305742**	**DUP**<br>**NML**<br>**INV/DUP**<br>**or**<br>**a tandem DUP**<br>**on H2**<br>**background**	**(+)102637533**<br>**(-)103256177**	**caGCTCAG-GCAgg**	**tc<TAGTGTTG>[c]a**	**No**	***Alu*Y/LTR (ERV1)**
**8942**	**DUP**	**389**	**102798929**<br>**103187725**	**Tandem DUP**	**(+)102798929**<br>**(+)103187725**	**agACAca**	**ag<t>ACAca**	**No**	**-/LCRC**
**8943**	**DUP**	**516**	**102591617**<br>**103107622**	**Tandem DUP**	**(+)102591617**<br>**(+)103107622**	**ggTACct**	**No**	**No**	**MER74A/L1PA5**
**8944**	**DUP**	**562**	**102511210**<br>**103073638**	**Tandem DUP**	**(+)102511210**<br>**(+)103073638**	**gtAAaAAagATTgACAt**<br>**g**	**No**	**No**	**-/L1ME4a**
**8945**	**DUP**	**440**	**102942249**<br>**103382339**	**Tandem DUP**	**(+)102942249**<br>**(+)103382339**	**gaGCTgt**	**No**	**No**	**-/LTR (MER101)**
**8946**	**DUP**	**362**	**102801949**<br>**103163822**	**Tandem DUP**	**(+)102801949**<br>**(+)103163822**	**No**	**aa(TTG)at**	**No**	**L1MA9/-**
**8947**	**DUP**	**367**	**102875648**<br>**103243088**	**Tandem DUP**	**(+)102875648**<br>**(+)103243088**	**ttAAgAAcATgGtg**	**No**	**No**	**(LTR)**<br>**MLT1A0/LCRA1a**
**8948**	**DUP**	**423**	**102845365**<br>**103268344**	**Tandem DUP**	**(+)102845365**<br>**(+)103268344**	**gtGGCATag**	**No**	**No**	**L1MC3/Chain (ID:**<br>**1065)**
**8949**	**DUP**	**998**	**102304420**<br>**103280891**	**Tandem DUP**<br>**with insertions**	**JP1**<br>**(+)103280891**<br>**(+)102308763**<br><br>**JP2**<br>**(+)102308855**<br>**(-)102303992**<br><br>**JP3**<br>**(-)102303224**<br>**(+)102304420**	**No**<br><br><br><br>**cgAgGGGAT-TAtt**<br><br><br><br>**ctGGGCgt**	**No**<br><br><br><br>**tt(CCCCTC)aa**<br><br><br><br>**No**	**No**<br><br><br><br>**No**<br><br><br><br>**Yes (88%**<br>**identity)**	**-/LTR (MLT1L)**<br><br><br><br>**LTR (MLT1L)/ L1MA3**<br><br><br><br>***Alu*Y/*Alu*Y**
**8950**	**DUP**	**429**	**102757828**<br>**103186867**	**Tandem DUP**<br>**with an**<br>**insertion**	**(+)103186867**<br>**(-)103026494**<br><br>**(-)103026479**<br>**(+)102757828**	**gtGGGaT-ATat**<br><br><br>**caGCCct**	**at(ATCCCCCCTCA)g**<br>**c is the result of TS**	**No**<br><br><br>**No**	**LCR (Item:**<br>**chrX:102190370)/LCRC**<br><br>**No**
**8951**	**DUP**	**488**	**102812954**<br>**103301556**	**Tandem DUP**	**(+)102812954**<br>**(+)103301556**	**No**	**tt(GT)ct**	**No**	**-/LTR49-int & Chain**<br>**(ID: 7704)**
**8952**	**DUP**	**427**	**102682377**<br>**103109731**	**Tandem DUP**	**(+)102682377**<br>**(+)103109731**	**gcCCCtt**	**g[c]CCCtt ; T>C**	**No**	**-/SINE (MIRb)**
**8953**	**DUP**	**425**	**102773657**<br>**103199137**	**Tandem DUP**	**(+)102773657**<br>**(+)103199137**	**gcTGggTTTGGaGAAcc**	**No**	**No**	**L1MC5/LCRC**
**8954**	**DUP**	**4,484**	**99762680**<br>**104246638**	**Tandem DUP**	**(+)99762680**<br>**(+)104246638**	**ctAGct**	**No**	**No**	***Alu*Y/-**

**8955**	**DUP1<br>&<br>DUP2**	**314<br><br>55**	**102925509<br>*103224075<br><br>103271708<br>*103305742**	**DUP<br>NM<br>INV/DUP<br>or<br>a tandem DUP<br>on H2<br>background**	**(-)103271708<br>(+)102925509**	**acCTGtACtCaa**	**No**	**No**	***Alu*/L1PA12**
**8956**	**DUP**	**758**	**102612197<br>103369941**	**Tandem DUP**	**(+)102612197<br>(+)103369941**	**taTCTcc**	**No**	**No**	**Low complexity (C-rich)/NA**
**8957**	**DUP**	**949**	**102150854<br>103099891**	**Tandem DUP**	**(+)102150854<br>(+)103099891**	**tcAGTTAaaAaTct**	**No**	**No**	**-/DNA (MER102a)**
**8958**	**DUP**	**257**	***102846600<br>*103104194**	**NA**	**NA**	**NA**	**NA**	**NA**	**NA**
**8959**	**DUP1**	**9**	**102671667<br>102680633**	**DUP<br>NML<br>DUP<br>NML<br>DUP<br>Or<br>a tandem DUP<br>with inherited<br>deletions**	**(+)102680633<br>(+)102682913**	**gtAaATGaCat**	**No**	**No**	**LTR(MLT1F2)/L1MD3**
	**DUP2**	**96**	**102682913<br>102779006**		**(+)102779006<br>(+)102809177**	**29bp sequence identity at JP**	**No**	**No**	**Chain (ID: 17283)/<br>Chain (ID: 84669)**
	**DUP3**	**546**	**102809177<br>103175979**		**(+)102671667<br>(+)103175979**	**tcAta**	**No**	**No**	**DNA(Tigger3b)/LCRC**
**8960**	**DUP1<br>&<br>DUP2**	**694<br><br>68**	***102546322<br>*103224075<br><br>*103258805<br>*103305742**	**DUP<br>NML<br>INV/DUP<br>or<br>a tandem DUP<br>on H2<br>background**	**NA**	**NA**	**NA**	**NA**	**NA**
**8961**	**DUP**	**841**	**102341097<br>103181893**	**Tandem DUP**	**(+)102341097<br>(+)103181893**	**ctCCAgc**	**No**	**No**	***Alu*Y/LCRC**
**8962**	**DUP**	**63**	**102765476<br>102801040**	**Two Tandem<br>DUPs**	**(+)102801040<br>(+)102765476**	**No**	**170bp insertion from two known region and small insertions of unknown origin**	**No**	**-/L1PB2/-/ L1MA9**
	**DUP**	**177**	**102929346<br>103106237**		**(+)103106237<br>(+)102929346**	**ccAtt**	**No**	**No**	**-/L1ME3A**
**8963**	**DUP**	**611**	**102678485<br>103289950**	**Tandem DUP**	**(+)102678485<br>(+)103289950**	**No**	**tc(CCAA)gc**	**No**	**L1M1/L1PA8 and Chain (ID: 118438)**
**8964**	**DUP**	**533**	**102560352<br>103093169**	**DUP<br>TRP/INV<br>DUP**	**(+)102560352<br>(-)103093169**	**tgCTGA-AAtTta**	**No**	**No**	**L1MEc/SINE(MIRc)**
	**TRP**	**130**	**103093169<br>*103224075**						
	**DUP**	**82**	***103224075<br>*103305742**			**NA**	**NA**	**NA**	**Presumed LCRA1a/LCRA1b chimera**

**8965**	**DUP1**	**456**	**102319763<br>102775516**	**DUP<br>NML<br>DUP<br>With two insertions at each junction**	**JP1<br>(+)102775516<br>(+)103352834**	**No**	**No**	**Yes**	**Chain (ID: 17283)/ LCRD**
					**JP2<br>(+)103352851<br>(+)103281343**	**atGTAtTTTCtGTgc**	**No**	**No**	**LCRD/-**
					**JP3<br>(+)103281363<br>(+)102836592**	**tgGAa-cAGga**	**GAa[c]AG(g)aa; T>C<br><br>38 Nt insertion is the result of two TS (JP1, JP2, JP3)**	**No**	**-/L1PA11**
	**DUP2**	**557**	**102836592<br>103352823**		**JP4<br>(+)103352823<br>(-)102775559**	**No**	**ga(GTAA)gt**	**No**	**-/Chain (ID: 17283)**
					**JP5<br>(-)102775516<br>(-)102775657**	**No**	**ct(GTG)aa**	**No**	**Chain (ID:17283)/Chain (ID: 17283)**
					**JP6<br>(-)102775578<br>(+)102319763**	**aaGtTGTCAGat**	**at(ACAT)tt<br><br>182 Nt insertion is the result of two TS (JP4, JP5, JP6)**	**No**	**Chain (ID: 17283)/LCRD**
**8966**	**DUP**	**397**	**103175456<br>102778246**	**Tandem DUP**	**(+)103175456<br>(+)102778246**	**NA**	**gg(GGGGACTG)ct**	**No**	**L1ME1/LCRC**
**8967**	**DUP**	**683**	**102364351<br>103057067**	**Tandem DUP**	**(+)102364351<br>(+)103057067**	**tgGATCgAagTGct**	**No**	**No**	**LTR(MER11A)/-**
**8968**	**DUP**	**122**	***102943944<br>*103066669**	**NA**	**NA**	**NA**	**NA**	**NA**	**NA**
**8969**	**DUP**	**404**	**102839729<br>103243779**	**Tandem DUP**	**(+)102839729<br>(+)103243779**	**No**	**cg(TA)tg**	**No**	**LTR (MER89)/LCRA1a**
**8970**	**DUP**	**93**	***102899075<br>*103050977**	**DUP<br>TRP/INV<br>DUP**	**NA**	**NA**	**NA**	**NA**	**NA**
	**TRP**	**59**	***103050977<br>*103224075**						
	**DUP**	**82**	***103224075<br>*103305742**			**NA**	**NA**	**NA**	**Presumed LCRA1a/LCRA1b chimera**
**8971**	**DUP**	**366**	***102938912<br>*103305265**	**NA**	**NA**	**NA**	**NA**	**NA**	**(Based on aCGH) NA/LCRA1a**

JP: Join-point, Nt: Nucleotide, Jct: Junction, NA: Not available, join-points are not resolved by Sanger sequencing, Dash (-): No repeat or repetitive element, Rep: replication.

Table S3. Original data from 7 studies on genomic rearrangements at the *PLP1* locus.

									Meta-analysis without re-interpretation of published data			This study only	
		Woodward *et al.* 2005 [a]	Lee *et al.* 2007 [b]	Carvalho *et al.* 2012	Bilir *et al.* 2013	Beck *et al.* 2015	Zhang *et al.* 2017	This study 2017	Total	% D [c]	%	% D	%
**A) Nature and pattern of CNV events based on array results [d]**													
**Single DUP events**		NA	7	-	4	-	29	33	73	134	54.5	50	66.0
**Complex genomic rearrangement (CGR) event**	**Total DUP-NML-DUP (disregarding directionality)**	NA	3	1	-	-	7	9	20	134	14.9	50	18.0
	**Total DUP-TRP-DUP (disregarding directionality)**	NA	1	-	-	15	8	3	27	134	20.1	50	6.0
	**Other complexities**	NA	3	-	-	1	5	5	14	134	10.4	50	10.0
	**Total CGR events**	NA	7	1	-	16	20	17	61	134	45.5	50	34.0
**Total unique CNV events**		NA	14	1	4	16	49	50	134	134	**100.0**	50	**100.0**
**B) Features of sequenced junctions [e]**													
**Microhomology (>1 bp)**		9	4	-	NA	11	27	15	60	159	37.7	57	26.3
**One bp sequence match**		1	-	-	NA	3	5	3	12	159	7.5	57	5.3
**Microhomeology**		NA	NA	NA	NA	NA	NA	19	25	NE	NE	57	33.3
**All insertions (>= 1bp)**		1	2	1	NA	4	23	13	44	159	27.7	57	22.8
**Blunt end**		2	-	-	NA	2	2	2	6	159	3.8	57	3.5
**Chimeric *Alu* [f]**		1	NA	-	NA	3	5	3	12	142	8.5	57	5.3
**Chimeric LINE [f]**		0	NA	-	NA	-	2	1	3	142	2.1	57	1.8
**NAHR [g]**		0	NA	NA	NA	NA	-	1	1	NE	NE	57	
**Total number of sequenced junctions**		12	5	2	0	21	62	57	159	Vari-able	100.0	57	100.0

									Meta-analysis without re-interpretation of published data			This study only	
		Woodward *et al.* 2005 [b]	Lee *et al.* 2007[a]	Carvalho *et al.* 2012	Bilir *et al.* 2013	Beck *et al.* 2015	Zhang *et al.* 2017	This study 2017	Total	% D [c]	%	% D	%

C) Genomic architectures overlapping sequenced join-points [d]

PMD-LCR	**LCRA1a**	1	-	-	NA	-		2	3	308	1.0	114	1.8
	**LCRA1b**	-	-	-	NA	-	-	2	2	308	0.6	114	1.8
	**LCRC**	-	1	-	NA	-	-	7	8	308	2.6	114	6.1
	**LCRD**	-	-	-	NA	-	-	3	3	308	1.0	114	2.6
	**LCR2**	-	-	-	NA	-	-	3	3	308	1.0	114	2.6
	**LCR3**	-	-	-	NA	-	-	1	1	300	0.3	114	0.9
	**Total PMD-LCR**	1	1	0	NA	0	0	18	20	308	6.5	114	15.8
**Segmental Duplications**		1	-	-	NA	1	2	2	6	300	2.0	114	1.8
**SelfChain [h]**		-	NA	2	NA	NA	NA	8	2	116	1.7	114	7.9
Repetitive elements	LINE	7	NA	1	NA	19	4	22	53	282	18.8	114	19.3
	Alu	7	NA	-	NA	7	8	8	30	282	10.6	114	7.0
	LTR	3	NA	1	NA	1	NA	13	18	158	11.4	114	11.4
**Observed/Examined architectures:** LCR, SelfChain, LINE, *Alu*, LTR, Purine/Pyrimidine tracts, Palindromes, Topoisomerase Consensus Cleavage sites, GAG seqs		8/8	1/1	3/5	NA	4/4	3/3	5/5	-	-	-	-	-
**None**		5	12	1	NA	14	110	25	167	308	54.2	114	21.9
**Total number of sequenced breakpoint junctions**		12	14	2	0	42	124	114	308	310	**100**	114	**100.0**

D) PMD-LCRs overlapping breakpoints at the level of aCGH for which sequencing was not possible

PMD-LCR	**LCRA1a/ LCRA1b**	NA	NA	-	NA	17	12	8	37

*Denominator used for calculating percentages.

[a] Number of unique samples. Samples that were studied further in more recent publications were excluded from the original publication count.

[b] Only samples with *PLP1* copy number gain were examined.

[c] Variable numbers due to variability of features examined in each of the published. A feature percentage was calculated based on the total number of samples obtained from studies that examined the corresponding feature. Studies that did not examine the corresponding feature were excluded from the total number.

[d] As observed at the level of aCGH or other high resolution CNV detection method

[e] As reported by authors for previously published cases (Without re-interpreting the data according to proposed parameters, some samples are reported in more than one study)

[f] Chimeric element: DNA rearrangement that occurs through homologous recombination between two *Alu* or LINE elements and generates a single chimeric, *Alu* or LINE element.

[g] NAHR: Homologous recombination between two highly similar non-allelic DNA sequences mediated by a pair of LCRs

[h] Alignments of the human genome with itself found in self-chain track on UCSC

NA: HD-aCGH data not available or specific parameter not evaluated by original authors

NE: Not examined in the majority of previous publications

Dash (-): none

Table S4. Microhomeologous sequences at the join-points found in this study.

<table>
<tr><th>No</th><th>Sample</th><th>Total No. of Nucleotides</th><th>No. of matching Nucleotides</th><th>Identity Matching (%)</th><th>Putative priming sequence (Top) &
Putative target annealing sequence (Bottom)
NA indicates no putative priming/target annealing available</th></tr>
<tr><td>1</td><td>BAB8924-1</td><td>9</td><td>7</td><td>78%</td><td>ACAAGCAAT
|| | ||||
ACCA-CAAT</td></tr>
<tr><td>2</td><td>BAB8924-2</td><td>8</td><td>6</td><td>75%</td><td>GAGGAAAG
||| |||
GAGCCAAG</td></tr>
<tr><td>3</td><td>BAB8930</td><td>10</td><td>8</td><td>80%</td><td>TTTCTGTTTA
||||| || |
TTTCTATTGA</td></tr>
<tr><td>4</td><td>BAB8934-1</td><td>10</td><td>8</td><td>80%</td><td>CATATGAGAA
||| |||||
CATGGGAGAA</td></tr>
<tr><td>5</td><td>BAB8934-2</td><td>12</td><td>9</td><td>75%</td><td>GACACATCATAG
||| ||| |||
GACTTATCTTAG</td></tr>
<tr><td>6</td><td>BAB8940</td><td>10</td><td>9</td><td>90%</td><td>GCTCAG-GCA
|||||| |||
GCTCAGAGCA</td></tr>
<tr><td>7</td><td>BAB8944</td><td>14</td><td>10</td><td>71%</td><td>AAAAAAGATT-ACA
|| || ||| ||| NA
AAGAACTATTGACA</td></tr>
<tr><td>8</td><td>BAB8947</td><td>10</td><td>7</td><td>70%</td><td>AAGAACATGG
|| || || | NA
AAAAATATAG</td></tr>
<tr><td>9</td><td>BAB8949-2</td><td>10</td><td>8</td><td>80%</td><td>AGGGGAT-TA
| ||||| ||
AAGGGATGTA</td></tr>
<tr><td>10</td><td>BAB8950-1</td><td>8</td><td>6</td><td>75%</td><td>GGGAT-AT
||| | ||
GGGTTCAT</td></tr>
<tr><td>11</td><td>BAB8953</td><td>13</td><td>10</td><td>77%</td><td>TGGGTTTGG-GAA
|| ||||| ||| NA
TGCCTTTGGAGAA</td></tr>
<tr><td>12</td><td>BAB8955</td><td>8</td><td>6</td><td>75%</td><td>CTGTACTC
||| || |
CTGGACCC</td></tr>
<tr><td>13</td><td>BAB8957</td><td>10</td><td>7</td><td>70%</td><td>AGTTAAAAAT
||||| | |
AGTTACCAGT</td></tr>
<tr><td>14</td><td>BAB8959</td><td>7</td><td>5</td><td>71%</td><td>A-ATG-C
| ||| |
AAATGAC</td></tr>
<tr><td>15</td><td>BAB8964</td><td>9</td><td>7</td><td>78%</td><td>CTGA-AATT
|||| || | NA
CTGACAACT</td></tr>
</table>

<table>
<tr><th></th><th></th><th></th><th></th><th></th><th></th></tr>
<tr><td>16</td><td>BAB8965-2</td><td>11</td><td>9</td><td>82%</td><td>GTATTTTCTGT
||| |||| ||
GTAATTTCAGT</td></tr>
<tr><td>17</td><td>BAB8965-3</td><td>7</td><td>5</td><td>71%</td><td>GAA-TAG
|| |||
GATGTAG</td></tr>
<tr><td>18</td><td>BAB8965-6</td><td>8</td><td>7</td><td>87%</td><td>GTTGTCAG
| ||||||
GCTGTCAG</td></tr>
<tr><td>19</td><td>BAB8967</td><td>10</td><td>7</td><td>70%</td><td>GATCGAAGTG
|||| | ||
GATC-ACATG</td></tr>
</table>

Table S5. Other features at the join-points found in this study.

No	Join-points with microhomology	Microhomology
1	BAB8920-2	CTGA
2	BAB8922	TC
3	BAB8926	GA
4	BAB8928-1	ATCTTCTGA
5	BAB8928-2	AGG
6	BAB8937	ATC
7	BAB8942	ACA
8	BAB8943	TAC
9	BAB8945	GCT
10	BAB8948	GGCAT
11	BAB8950-2	GCC
12	BAB8952	CCC
13	BAB8954	AG
14	BAB8956	TCT
15	BAB8961	CCA

No	Join-points with Insertions
1	BAB8921
2	BAB8923
3	BAB8929
4	BAB8933
5	BAB8938
6	BAB8946
7	BAB8951
8	BAB8962-1
9	BAB8963
10	BAB8965-4
11	BAB8965-5
12	BAB8966
13	BAB8969

No	Join-points with chimeric *Alus*, LINEs, Self-Chains
1	BAB8923-1 (*Alu*)
2	BAB8934-3 (*Alu*)
3	BAB8942-3 (*Alu*)
4	BAB8920-1 (LINE)
5	BAB8959-3 (Self-Chain)

No	Join-points with blunt end
1	BAB8949-1
2	BAB8965-1

No	Join-points with 1 bp sequence match
1	BAB8935
2	BAB8959-1
3	BAB8962-2

Table S6. Microhomeologous sequences at the join-points found by re-analyzing breakpoint sequences from previous studies.

<table>
<tr><th>No</th><th>Sample</th><th>Total No. of Nucleotides</th><th>No. of matching Nucleotides</th><th>Identity Matching (%)</th><th>Putative priming sequence (Top) & Putative target annealing sequence (Bottom)
NA indicates no putative priming/target annealing available</th><th>Previously reported microhomology</th><th>Reference</th></tr>
<tr><td>1</td><td>P026</td><td>11</td><td>8</td><td>73%</td><td>CTGACACCTCA
|| | |||||
CTCAGTCCTCA</td><td>CCTCA
|||||
CCTCA</td><td>1</td></tr>
<tr><td>2</td><td>P083</td><td>5</td><td>4</td><td>80%</td><td>ACA-A
||| |
ACATA</td><td>ACA
|||
ACA</td><td>1</td></tr>
<tr><td>3</td><td>P116</td><td>6</td><td>5</td><td>83%</td><td>CAATGA
|| |||
CAGTGA</td><td>TGA
|||
TGA</td><td>1</td></tr>
<tr><td>4</td><td>P176 (PMD7)</td><td>8</td><td>7</td><td>87%</td><td>ATAA-TAT
|||| |||
ATAACTAT</td><td>TAT
|||
TAT</td><td>1</td></tr>
<tr><td>5</td><td>P224</td><td>6</td><td>5</td><td>83%</td><td>AAGGTC
|| |||
AA-GTC</td><td>GTC
|||
GTC</td><td>1</td></tr>
<tr><td>6</td><td>PMD9</td><td>7</td><td>5</td><td>71%</td><td>CTGCCTG
|| |||
CT--CTG</td><td>CTG
|||
CTG</td><td>1</td></tr>
<tr><td>7</td><td>BAB2448</td><td>13</td><td>10</td><td>77%</td><td>TGGGTTTGG-GAA
|| ||||| ||| NA
TGCCTTTGGAGAA</td><td>TTTGG
|||||
TTTGG</td><td>2</td></tr>
<tr><td>8</td><td>1264-2</td><td>14</td><td>11</td><td>79%</td><td>AGTCCAGTTAGCCC
||| |||| ||||
AGTTCAGTG-GCCC</td><td>AGT
|||
AGT</td><td>2</td></tr>
<tr><td>9</td><td>BAB2867</td><td>7</td><td>5</td><td>71%</td><td>TTCATCA
| ||| |
TCCATGA</td><td>CAT
|||
CAT</td><td>3</td></tr>
<tr><td>10</td><td>P250</td><td>10</td><td>8</td><td>80%</td><td>TTGACTGGCC
|||| ||||
TTGA--GGCC</td><td>-</td><td>4</td></tr>
<tr><td>11</td><td>P255</td><td>7</td><td>5</td><td>71%</td><td>AATGCTC
|| || |
AAAGCCC</td><td>AA
||
AA</td><td>4</td></tr>
<tr><td>12</td><td>P820</td><td>12</td><td>9</td><td>75%</td><td>ATT-AGGAGAAA
||| | ||| ||
ATTGATGAGCAA</td><td>AA
||
AA</td><td>4</td></tr>
<tr><td>13</td><td>P1150-1</td><td>7</td><td>5</td><td>71%</td><td>AGG-AAG
|| |||
AGTCAAG</td><td>AG
||
AG</td><td>4</td></tr>
<tr><td>14</td><td>P1407</td><td>7</td><td>5</td><td>71%</td><td>TGCTCAT
|| || |
TG-TCCT</td><td>TG
||
TG</td><td>4</td></tr>
<tr><td>15</td><td>J003-1</td><td>6</td><td>5</td><td>83%</td><td>AGAGCC
||| ||
AGATCC</td><td>CC
||
CC</td><td>5</td></tr>
<tr><td>16</td><td>J003-2</td><td>13</td><td>10</td><td>77%</td><td>AGAAATGCAG-CA
||||| | || ||
AGAAAAG-AGACA</td><td>AGAAA
|||||
AGAAA</td><td>5</td></tr>
</table>

<table>
<tr><td>17</td><td>J016</td><td>10</td><td>7</td><td>70%</td><td>TTTTGATACA
||| || | | NA
TTTAGAAAAA</td><td>GA
||
GA</td><td>5</td></tr>
<tr><td>18</td><td>J029-2</td><td>6</td><td>5</td><td>83%</td><td>GGTATG
|| |||
GGGATG</td><td>ATG
|||
ATG</td><td>5</td></tr>
<tr><td>19</td><td>J035</td><td>9</td><td>7</td><td>78%</td><td>GCTAGAGTA
||| ||| |
GCT-GAGGA</td><td>GCT
|||
GCT</td><td>5</td></tr>
<tr><td>20</td><td>J038</td><td>7</td><td>5</td><td>71%</td><td>GTTAAAC
| || || NA
GCTATAC</td><td>TA
||
TA</td><td>5</td></tr>
<tr><td>21</td><td>J041</td><td>8</td><td>6</td><td>75%</td><td>ATCCTCCA
||| |||
ATC—-CCA</td><td>CCA
|||
CCA</td><td>5</td></tr>
<tr><td>22</td><td>J050-2</td><td>8</td><td>7</td><td>87%</td><td>AATACTGT
|| |||||
AAGACTGT</td><td>ACTGT
|||||
ACTGT</td><td>5</td></tr>
<tr><td>23</td><td>J065</td><td>10</td><td>7</td><td>70%</td><td>ATTGCATCAC
||| || ||
ATTA-ATAAC</td><td>ATT
|||
ATT</td><td>5</td></tr>
<tr><td>24</td><td>J068-1</td><td>7</td><td>5</td><td>71%</td><td>CAAGAAC
| ||| |
C-AGACC</td><td>C
|
C</td><td>5</td></tr>
<tr><td>25</td><td>J068-2</td><td>8</td><td>6</td><td>75%</td><td>TTCTCCGT
|||| ||
TTCT--GT</td><td>TTCT
||||
TTCT</td><td>5</td></tr>
<tr><td>26</td><td>J079-1</td><td>15</td><td>11</td><td>73%</td><td>ATGAATGAAATTGTA
||||| || ||||
ATGAA--AA--TGTA</td><td>ATGAA
|||||
ATGAA</td><td>5</td></tr>
<tr><td>27</td><td>J080</td><td>8</td><td>7</td><td>87%</td><td>CAGGCCCA
||||| ||
CAGGCACA</td><td>CAGGC
|||||
CAGGC</td><td>5</td></tr>
<tr><td>28</td><td>J082</td><td>15</td><td>12</td><td>80%</td><td>TGC-CCAGGCTGGAC
||| |||||| |||
TGCACCAGGCA—GAC</td><td>TGC
|||
TGC</td><td>5</td></tr>
<tr><td>29</td><td>J083</td><td>8</td><td>6</td><td>75%</td><td>CTTC-ACT
|| | |||
CTGCAACT</td><td>ACT
|||
ACT</td><td>5</td></tr>
<tr><td>30</td><td>J102</td><td>14</td><td>11</td><td>79%</td><td>TTG--CTATACAAG
||| | |||||||
TTGAGC-AGACAAG</td><td>TTG
|||
TTG</td><td>5</td></tr>
<tr><td>31</td><td>J103</td><td>7</td><td>6</td><td>86%</td><td>ATATGTC
|||| ||
ATATCTC</td><td>ATAT
||||
ATAT</td><td>5</td></tr>
<tr><td>32</td><td>J108</td><td>15</td><td>11</td><td>73%</td><td>AAAATTATGAGATTT
||||| | ||| ||
AAAATC-TCAGACTT</td><td>AAAAT
|||||
AAAAT</td><td>5</td></tr>
</table>

References

1. Woodward KJ, Cundall M, Sperle K, Sistermans EA, Ross M, Howell G, et al. Heterogeneous duplications in patients with Pelizaeus-Merzbacher disease suggest a mechanism of coupled homologous and nonhomologous recombination. Am J Hum Genet. 2005;77:966–87.

2. Lee JA, Carvalho CMB, Lupski JR. A DNA Replication Mechanism for Generating Nonrecurrent Rearrangements Associated with Genomic Disorders. Cell. 2007;131:1235–47.

3. Carvalho CMB, Bartnik M, Pehlivan D, Fang P, Shen J, Lupski JR. Evidence for disease penetrance relating to CNV size: Pelizaeus-Merzbacher disease and manifesting carriers with a familial 11 Mb duplication at Xq22. Clin Genet. 2012;81:532–41.

4. Beck CR, Carvalho CMB, Banser L, Gambin T, Stubbolo D, Yuan B, et al. Complex Genomic Rearrangements at the *PLP1* Locus Include Triplication and Quadruplication. PLoS Genet. 2015;11:1–26.

5. Zhang L, Wang J, Zhang C, Li D, Carvalho CMB, Ji H, et al. Efficient CNV breakpoint analysis reveals unexpected structural complexity and correlation of dosage-sensitive genes with clinical severity in genomic disorders. Hum Mol Genet. 2017;26:1927–41.
